# Decoding Octopus Skin Mucus: Impact of Aquarium-Maintenance and Senescence on the Proteome Profile of the Common Octopus (*Octopus vulgaris*)

**DOI:** 10.3390/ijms25189953

**Published:** 2024-09-15

**Authors:** Sara Pérez-Polo, Alejandro Rivero Mena, Lorena Barros, Paula Borrajo, Manuel Pazos, Mónica Carrera, Camino Gestal

**Affiliations:** Instituto de Investigaciones Marinas (IIM-CSIC), Spanish National Research Council (CSIC), Eduardo Cabello 6, 36208 Vigo, Spain; sperez@iim.csic.es (S.P.-P.); arime@mail.ucv.es (A.R.M.); lorenab@iim.csic.es (L.B.); pborrajo@iim.csic.es (P.B.); mpazos@iim.csic.es (M.P.)

**Keywords:** *Octopus vulgaris*, skin mucus, proteomics, mass spectrometry, bioactive peptides, food, welfare, non-invasive methods, aquarium-maintained, senescent, wild

## Abstract

The common octopus (*Octopus vulgaris)* is an excellent candidate for aquaculture diversification, due to its biological traits and high market demand. To ensure a high-quality product while maintaining welfare in captive environments, it is crucial to develop non-invasive methods for testing health biomarkers. Proteins found in skin mucus offer a non-invasive approach to monitoring octopus welfare. This study compares the protein profiles in the skin mucus of wild, aquarium-maintained, and senescent specimens to identify welfare biomarkers. A tandem mass tag (TMT) coupled with an Orbitrap Eclipse Tribrid mass spectrometer was used to create a reference dataset from octopus skin mucus, identifying 1496 non-redundant protein groups. Although similar profiles were observed, differences in relative abundances led to the identification of potential biomarkers, including caspase-3-like, protocadherin 4, deleted in malignant brain tumors, thioredoxin, papilin, annexin, cofilin and mucin-4 proteins. Some of these proteins also revealed potential as bioactive peptides. This investigation provides the most extensive analysis of the skin mucus proteome in the common octopus and is the first to explore how aquarium maintenance and senescence alter the mucus proteome. This research highlights the potential of skin mucus protein/peptides as non-invasive monitoring biomarkers in cultured animals.

## 1. Introduction

The common octopus holds significant global relevance among cephalopod species. Economically, it stands out as the species with the longest history of human exploitation through important fisheries [1]. Additionally, its unique characteristics make it a fascinating subject for scientific research [2]. This cephalopod is characterized by rapid growth, a short life cycle, and sophisticated sense organs with learning and memory capabilities. This is associated with its ability to adapt quickly to environmental challenges, making them a resilient group, which may benefit from a changing oceanic environment [3].

Octopus demand and fishery catches have risen over the past 30 years from 179,000 to 350,000 tons [4]. However, in species that were already in high demand, this increase has resulted in over-exploitation, which has generated instability and loss of natural stocks [5]. This is the case of the common octopus (*Octopus vulgaris*), for which a decline in catches has been observed in recent years [6]. Decreasing catches and some biological characteristics of this species make it as an excellent candidate for European aquaculture diversification. The establishment of common octopus farming might help to protect natural stocks from overfishing, enabling sustainable management of the resource, while simultaneously satisfying the increasing market demand for this species.

In aquaculture, optimal conditions must be ensured to guarantee animal welfare. Criteria for assessing welfare conditions should focus primarily on diet, appropriate housing conditions, and living environment, as well as behavior and health. These measures should ensure the welfare of the animals while at the same time aiming to achieve a higher quality product, safer for consumption and nutritionally equivalent to wild organisms. Under culture conditions, animals are exposed to environmental stimuli and restrictions that differ from those under natural conditions, resulting in different stress factors that may have varying effects on individuals. Identification of welfare biomarkers in culture conditions, and their implementation in health and welfare monitoring plans, would help to guarantee good and sustainable aquaculture practices in the future.

The skin of cephalopods, as with fish and other mollusks, is covered by mucus, which is mainly formed by mucins (glycoproteins and proteoglycans), water, electrolytes, epithelial and blood cells, and a wide range of bioactive molecules such as proteolytic enzymes, lysozymes, antioxidants, and antimicrobial peptides [7,8,9]. These components play a critical role in animal defense, acting as a barrier against a wide variety of chemical, physical, and biological stressors. The mucus also serves as a repository of numerous innate immune components, including molecules with antimicrobial activity [7,10,11,12].

The recent global proteomic characterization of *O. vulgaris* skin mucus opens new possibilities for the identification of welfare biomarkers in this species [11]. The study of mucosal immunity of aquatic species and the analysis of several immune factors or enzymes in skin mucus samples have been regarded as low-invasive approaches for the identification of health-related biomarkers in fish and mollusks subjected to stressful conditions [7,12,13]. Therefore, the study of protein profiles in the skin mucus of the common octopus under different conditions could be a powerful tool for obtaining welfare biomarkers by a non-invasive, easy, and effective method.

Multiple studies demonstrate that captive conditions can modify the protein expression profiles and abundances of different molecules in various tissues in fish [14,15,16,17,18,19], as well as in other marine organisms, where differences in bioactive molecules and haemolymph have been demonstrated [20,21]. These differences could probably be observed in the same way in cephalopods, although no in-depth studies have yet explored this possibility.

Additionally, the identification of the different life stages of cephalopods is a very relevant issue for aquaculture purposes, as it involves major physiological, immunological, and behavioral changes. One of the most relevant biological conditions to identify in aquaculture systems is the senescent stage, which occurs at the end of sexual maturity, after a single reproductive bout, and often occurs just before death. Changes observed at this stage include loss of appetite, retraction of the skin around the eyes, loss of coordination, and the appearance of non-healing skin lesions. In females, senescence begins while the female is rearing eggs or shortly after hatching, while in males it usually begins after mating [22,23,24,25,26]. Senescence is probably part of a hormonally regulated adaptive program, which is difficult to identify in the wild because senescent animals are usually predated. In cultured or aquarium-maintained animals, the situation is different, and it is important to identify the senescence stage early and distinguish it from potential breeding or disease issues. Some non-invasive hormonal studies [27,28] have demonstrated an increase in stress-related hormones in senescent octopuses. Similarly, senescence may cause changes in the protein/peptide profiles of cephalopods [22,23,24].

Proteomics allows the detection of variations in protein abundance in similar genetic contexts, making it a very powerful tool for detecting alterations and differences between wild and aquarium organisms [29], as well as for understanding the physiological and pathological mechanisms underlying various biological processes, such as senescence. For this reason, the main objective of this study is to compare the different protein profiles associated with different developmental and maintenance conditions (aquarium, wild, and senescent) in the common octopus. This comparison seeks to identify differences associated with these conditions and to enable the early identification of senescent octopuses. The proposed tool can be validated as a non-invasive method for the identification of skin mucus proteins/peptides that could be used as biomarkers of health and welfare in cultured animals. Additionally, this study includes a preliminary analysis of sex-related differences in protein profiles in senescent octopuses, and it concludes with an investigation into potential bioactive peptides of pharmacological interest in the skin mucus of this species.

## 2. Results

### 2.1. Protein Profiles by SDS-PAGE

**Figure 1:**
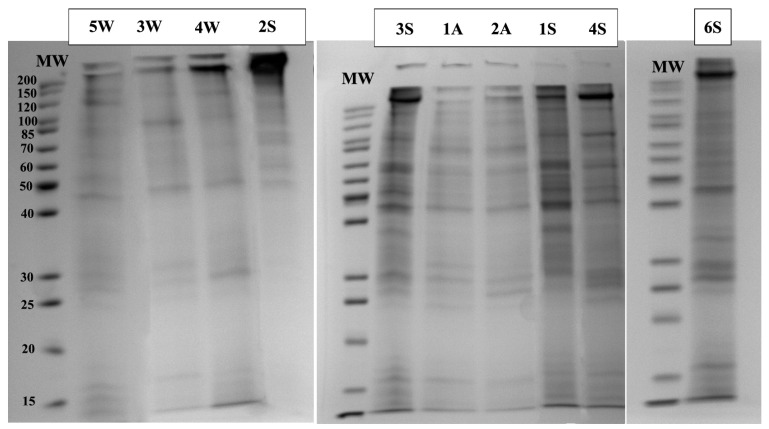
SDS-PAGE 12% profiles of the skin mucus protein for ten samples (1A, 1S, 2A, 2S, 3W, 3S, 4W, 4S, 5W, 6S); A: aquarium-maintained; W: wild; S: senescent. MW (molecular weight in KDa).

Proteins extracted from the skin mucus were separated based on their molecular weight using SDS-PAGE 12% to display the corresponding electrophoretic profile of each sample (Figure 1). Profiles of different samples showed slight variability in the separation of the proteins and their relative concentration.

### 2.2. Mass Spectrometry (MS)

As a result of LC-MS/MS analysis, a total of 1496 non-redundant protein groups were identified (Supplementary Data S1). The proteome data were obtained by merging a total of 15,629 identified spectra (Peptide Spectrum Matches, PSMs) from 7480 different peptides (Supplementary Data S2). Raw data and analysis outputs are publicly available in the ProteomeXchange database (https://www.proteomexchange.org/, accessed on 7 June 2024), via the PRIDE repository, and have been assigned to the identifier PXD052915.

The TMT-based quantitative proteomics experiments were used to create a proteome dataset and protein list for *O. vulgaris* skin mucus under 3 different conditions: wild-type or natural population (3W, 4W, 5W), aquarium-maintained (1A, 2A) and senescent (1S, 2S, 3S, 4S, 6S). Of these 1496 non-redundant protein groups, 1462 non-redundant protein groups were identified in the wild group, 1460 non-redundant protein groups in the aquarium-maintained group, and 1476 in the senescent group. Supplementary Data S1 summarizes the total non-redundant proteins, wild group proteins, aquarium-maintained group proteins, and proteins from the senescent group of common octopus skin mucus. The final global dataset of the 3 different conditions of *O. vulgaris* skin mucus proteome was investigated by quantitative analysis and protein-based bioinformatics, including gene ontologies, pathways, network analyses, and prediction of potential bioactive peptides. Additionally, specific samples corresponding to the same specimen under different conditions or life stages have been analyzed under different conditions (wild vs. senescent or maintained in aquarium vs. senescent).

### 2.3. TMT Based Quantitative Analysis of MS Data

#### 2.3.1. Analysis by Condition

**Figure 2:**
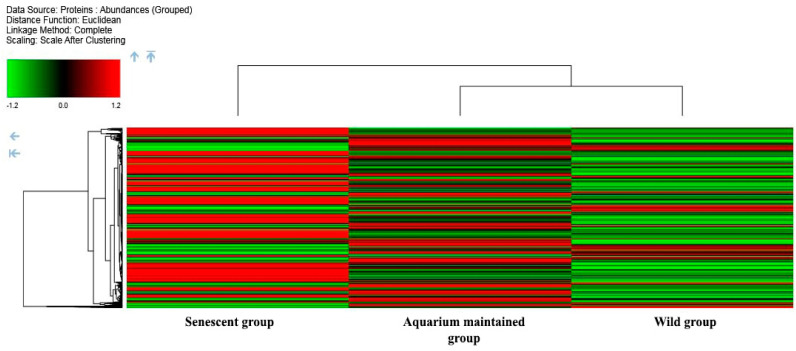
Heatmap from the proteomics analysis of the skin mucus samples of octopus under the three different conditions: wild group (3W, 4W, 5W), aquarium-maintained group (1A, 2A), and senescent group (1S, 2S, 3S, 4S, 6S). Bars correspond to the up-regulation (red) or down-regulation (green) of particular proteins. Euclidean hierarchical distances were sorted for all samples.

The distribution of all the proteins across the 3 condition samples analyzed by TMT is presented in a heatmap diagram in Figure 2. Euclidean hierarchical distance was used to differentiate the two main clusters: Cluster A (senescent group) and Cluster B (aquarium-maintained and wild group). Protein abundance differences among the three condition groups are shown.

**Figure 3:**
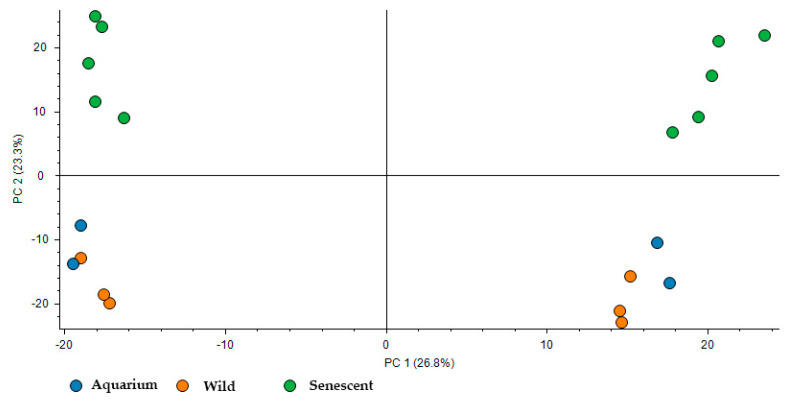
PCA analysis of the samples grouped by conditions (aquarium-maintained group in blue, wild group in orange, and senescent group in green).

The PCA analysis of the samples grouped by conditions (Figure 3) shows a clear difference in the segregation of the senescent condition group compared to the other conditions. On the other hand, the wild group and the aquarium-maintained group show slight differences, although these differences are reflected more slightly in the analysis. In particular, Principal Component 2 (which summarizes 23.3% of the variability) shows an important discrimination effect based on the life cycle stage mainly between the senescent condition and the other conditions.

Protein abundance statistical significance, with the magnitude of the fold-change (abundance ratio), was studied between the three condition groups using volcano plots (Supplementary Data S3). Proteins within the green area are down-regulated (FC < –1.0 and (*p* < 0.05)) and the ones in the red area are up-regulated (FC > 1.0 and (*p* < 0.05)).

**Figure 4:**
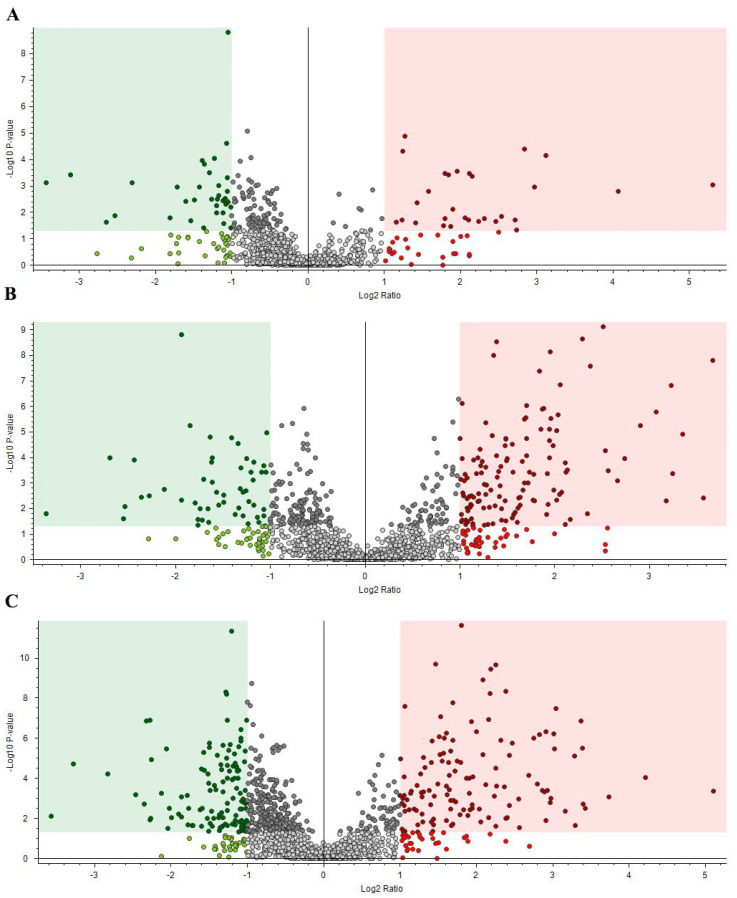
Volcano plot analysis by condition. (**A**) Wild group vs. aquarium-maintained group. (**B**) Aquarium-maintained group vs. senescent group. (**C**) Wild group vs. senescent group. The red area corresponds to up-regulated proteins and the green area to down-regulated proteins.

The volcano plot comparing the wild group to the aquarium-maintained group (Figure 4A) reveals 29 up-regulated proteins and 35 down-regulated proteins. The up-regulated proteins, which were significantly more abundant in the wild group, include mammalian ependymin-related protein, deleted in malignant brain tumors protein, SCO-spondin-like, and several uncharacterized proteins. In contrast, the down-regulated proteins, more abundant in the aquarium-maintained group, include various caspases, zinc finger protein 318, EF-hand domain-containing protein, copper transport protein, neural cell adhesion molecule, aminopeptidase W07G4.4, deoxynucleoside triphosphohydrolase SAMHD1, cathepsin protein, and actin protein.

The aquarium-maintained group vs. the senescent group volcano plot (Figure 4B) shows 131 up-regulated proteins and 52 down-regulated proteins. Up-regulated proteins included insulin-like growth factor-binding protein, von Willebrand factor C domain-containing protein, mucin 19 protein, tetraspanin proteins, myb-like protein, cadherin 23 protein, histone H1, MAM and LDL-receptor class A domain-containing protein, poly [ADP-ribose] polymerase, cytochrome P450 2B2 and 28 different uncharacterized proteins among others. Down-regulated proteins included a 70 kDa neurofilament protein, interferon-induced protein, ACTB_G1, Roadblock/LAMTOR2 domain-containing protein, golgi-associated plant pathogenesis-related protein, cystathionine beta-synthase, mucin 4 protein, and different uncharacterized proteins that were more abundant in the senescent group.

The wild group vs. the senescent group volcano plot (Figure 4C) shows 126 up-regulated proteins and 119 down-regulated proteins. Among the up-regulated proteins anoctamin, SCO-spondin, mucin 19 proteins, neurotrypsins, barrier-to-autointegration factor B, cytochrome P450 2B2, saposin B-type domain-containing protein, tetraspanin, deleted in malignant brain tumors protein, ferroxidase, allene oxide synthase-lipoxygenase protein, artichoke protein, histone H1, and numerous uncharacterized proteins were found. Down-regulated proteins, more abundant in the senescent group included switch-associated protein 70, coronin, V-type proton ATPase subunit C, calpain 11, RING-type E3 ubiquitin transferase, von Willebrand factor A domain-containing protein, caspase 3, Twinfilin-1, talin protein, 70 kDa neurofilament protein, double-stranded RNA-specific editase 1, LTD domain-containing protein, Small Nuclear Ribonucleoprotein Polypeptides B (SNRPB), titin, purple acid phosphatase, thimet oligopeptidase, universal stress protein in QAH/OAS sulfhydrylase 3′region protein, among others.

#### 2.3.2. Time–Course Specimen Analysis

Four specimens of common octopus have been analyzed over time, satisfying two of the conditions studied. The results of the relative abundance in the skin mucus proteome of the same specimen when changing conditions were obtained using volcano plots. The detailed results of this section are shown in Supplementary Data S4.

**Figure 5:**
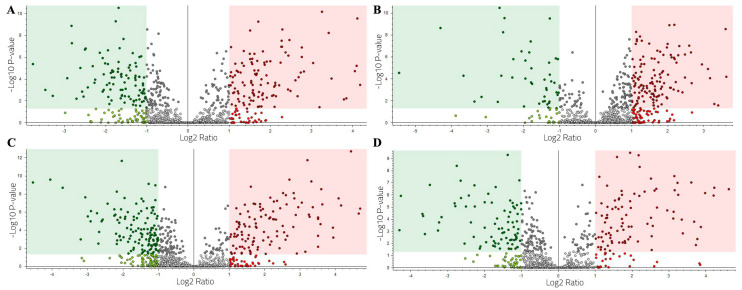
Volcano plot analysis by specimen. (**A**) Octopus specimen 1: Aquarium-maintained vs. senescent. (**B**) Octopus specimen 2: Aquarium-maintained vs. senescent. (**C**) Octopus specimen 3: Wild vs. senescent. (**D**) Octopus specimen 4: Wild vs. senescent. The red area corresponds to up-regulated proteins and the green area to down-regulated proteins.

Specimen 1 was studied after a period of 3 months of aquarium maintenance and also when it reached the senescent stage. The resulting volcano plot (Figure 5A) shows 109 up-regulated proteins and 96 down-regulated proteins. The most outstanding up-regulated proteins were MAM and LDL-receptor class A domain-containing protein, glutamine synthetase, tumor protein D52 isoform, chitinase, neural cell adhesion molecule, tetraspanin proteins, transcription factor iws-1 isoform, cytochrome c oxidase subunit, caspase 7 protein, histone H1, mucin 19, insulin-like growth factor-binding protein complex acid labile subunit isoform X1, VWFD domain-containing protein, and neuroglian isoform. The most abundant down-regulated proteins, which were more abundant in the senescent condition, include mucin 4, ACTB_G1, thyroglobulin isoform protein, 70 kDa neurofilament protein, H15 domain-containing protein, vacuolar proton pump subunit B, EF-hand domain-containing protein, V-type proton ATPase subunit E, deoxynucleoside triphosphate triphosphohydrolase SAMHD1, universal stress protein in QAH/OAS sulfhydrylase 3′region isoform X1, caspase 3, H(+)-transporting two-sector ATPase, 7.1.2.2 catalytic subunit A, omega-crystallin, titin, coronin, and golgi-associated plant pathogenesis-related protein 1-like.

Specimen 2 was studied after a period of 3 months of aquarium-maintenance and also when it reached its senescent stage. The volcano plot analysis (Figure 5B) shows 125 up-regulated proteins and 42 down-regulated proteins. The most relevant up-regulated proteins were mucin 19, caspase 3, histone H1 and H3, MAM and LDL-receptor class A domain-containing protein, fascin, 60–70 KDa neurofilament protein-like, neuroglian isoform, barrier-to-autointegration factor B, calpain-9-like, protocadherin Fat 4, neural cell adhesion molecule 2 isoform X13, CCHC-type zinc finger nucleic acid binding protein, chitinase, tetraspanin, and chaperone protein. The most abundant down-regulated proteins were hemocyanin 1, intermediate filament protein ifa-1-like, von Willebrand factor A domain-containing protein, kynurenine–oxoglutarate transaminase, actin related protein 2/3 protein complex subunit p16, succinate–CoA ligase [GDP-forming] subunit beta, mitochondrial, annexin, deoxynucleoside triphosphate triphosphohydrolase SAMHD1-like isoform X2, DUF4773 domain-containing protein, mucin 4, CuZn superoxide dismutase, transforming growth factor-beta-induced protein ig-h3-like isoform, and CD109 antigen isoform protein, among others.

Specimen 3 was studied in a wild stage (after a period of 3 days of acclimation in the aquarium) and also when it reached its senescent stage after 8 months of being maintained in the aquarium facilities. The volcano plot analysis (Figure 5C) shows a total of 113 up-regulated proteins and 125 down-regulated proteins. The most abundant up-regulated proteins in the wild stage were chitinase, mucin 19 proteins, histone H1, deleted in malignant brain tumors 1 protein-like, adhesion G-protein coupled receptor, cytochrome c oxidase copper chaperone, neutral and basic amino acid transport protein rBAT, protocadherin Fat 4, barrier-to-autointegration factor B, SCO-spondin protein, caspase 7, 60S ribosomal protein L13 and L7, protocadherin Fat 4, ras-related protein Rab-3, and MAM-LDL-receptor class A domain-containing protein. The down-regulated proteins identified included caspase 3, collagen alpha 4, LLGL scribble cell polarity complex component 2 isoform X2, transgelin, mucin 4, tropomyosin Tod p 1.0102, VWFA domain-containing protein, heat shock protein 83 isoform X1, death domain-containing protein CRADD, gastrotropin, titin, actin related protein 2/3 protein complex subunit p16, succinate-CoA ligase [GDP-forming] subunit beta, purple acid phosphatase, SH3 domain-containing protein, RING-type E3 ubiquitin transferase, calpain 9, and universal stress protein in QAH/OAS sulfhydrylase 3′region isoform X1.

Specimen 4 was also studied in its wild stage (after a period of 3 days of acclimation in the aquarium) and also when it reached its senescent stage after 2 months of being maintained in the aquarium facilities. The results of the volcano plot (Figure 5D) were represented by 88 up-regulated proteins and 79 down-regulated proteins. Up-regulated proteins included MAM and LDL-receptor class A domain-containing protein, mucin protein, chitinase proteins, SCO-spondin-like proteins, Hemocyanin A-type, Von Willebrand factor C domain-containing protein, Ras-related proteins, annexin, death domain-containing protein CRADD, deleted in malignant brain tumors 1 proteins-like, collagen alpha-3 and 4, transforming growth factor-beta-induced protein ig-h3-like, cartilage matrix proteins, serine protease 1, ferritin, neutral and basic amino acid transport protein rBAT, EGF-containing fibulin-like extracellular matrix protein, protein kinases and many uncharacterized proteins. The most relevant down-regulated proteins, which are more abundant in the senescent stage, include caspase 3, deoxynucleoside triphosphate triphosphohydrolase SAMHD1, neural cell adhesion molecule 2 isoform X13, heat shock protein, omega-crystallin, cofilin, actin-like proteins, intermediate filament protein ifa-1-like isoform X1, thioredoxin-disulfide reductase, gelsolin-like protein, Rho GDP dissociation inhibitor, golgi-associated plant pathogenesis-related protein 1-like, aminopeptidase, 3.4.11, glutamate dehydrogenase, mucin 3-like protein, ubiquitin related proteins, serine/threonine-related proteins, collagen alpha-1(I) chain isoform X1 and tubulin alpha-3 chain, among others.

#### 2.3.3. Preliminary Sex-Specific Study of Senescent Animals

**Figure 6:**
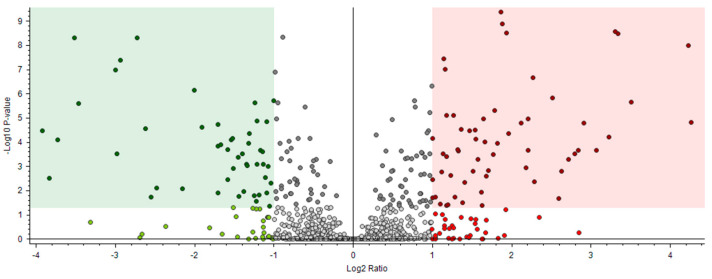
Senescent male vs. senescent female volcano plot. The red area corresponds to up-regulated proteins and the green area to down-regulated proteins.

The differences between senescent males and senescent females were studied by using a volcano plot (Figure 6) to observe significant differences in their relative abundance in protein content. These results were obtained from a preliminary study, as the limited sample size only allows an introductory approach to potential sex-associated differences in octopus senescence.

Results showed 58 up-regulated proteins and 47 down-regulated proteins. The most abundant up-regulated proteins in the senescent male were thioredoxin-dependent peroxiredoxin, mucin-19 protein, collagen, cofilin, chitinases, SCO-spondin protein, tetraspanin, regulacin, annexin, histone H3, ras-related protein Rab-3, lamin-B1 isoform, papilin, kyphoscoliosis peptidase, alpha tubulin and numerous uncharacterized proteins. The most abundant down-regulated proteins, which were more abundant in the senescent female, were deoxynucleoside triphosphate triphosphohydrolase SAMHD1, C1 family cathepsin L2, calcium-dependent protein kinase 12 isoform X2, beta-tubulin protein, EF-hand domain-containing protein, RING-type E3 ubiquitin transferase, mucin 4 protein, neural cell adhesion molecule 2 isoform, titin proteins, intermediate filament protein ifa-1-like isoform proteins, and golgi-associated plant pathogenesis-related protein 1-like.

### 2.4. Selection of Potential Protein Biomarkers

#### 2.4.1. Analysis by Condition

**Figure 7:**
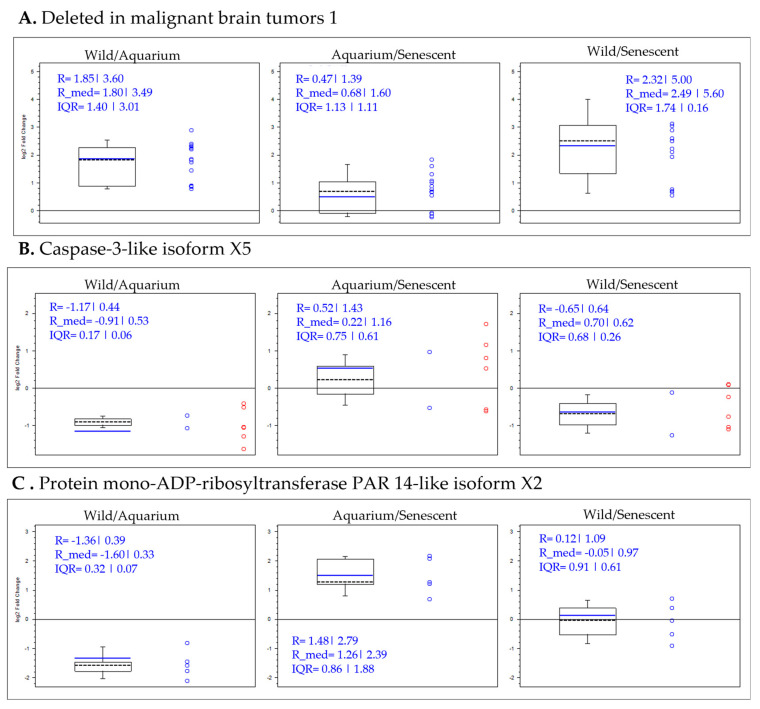
Box plot representations of the quantification ratios of selected protein biomarkers by conditions, more abundant in wild (**A**) and aquarium-maintained groups (**B**,**C**).

**Figure 8:**
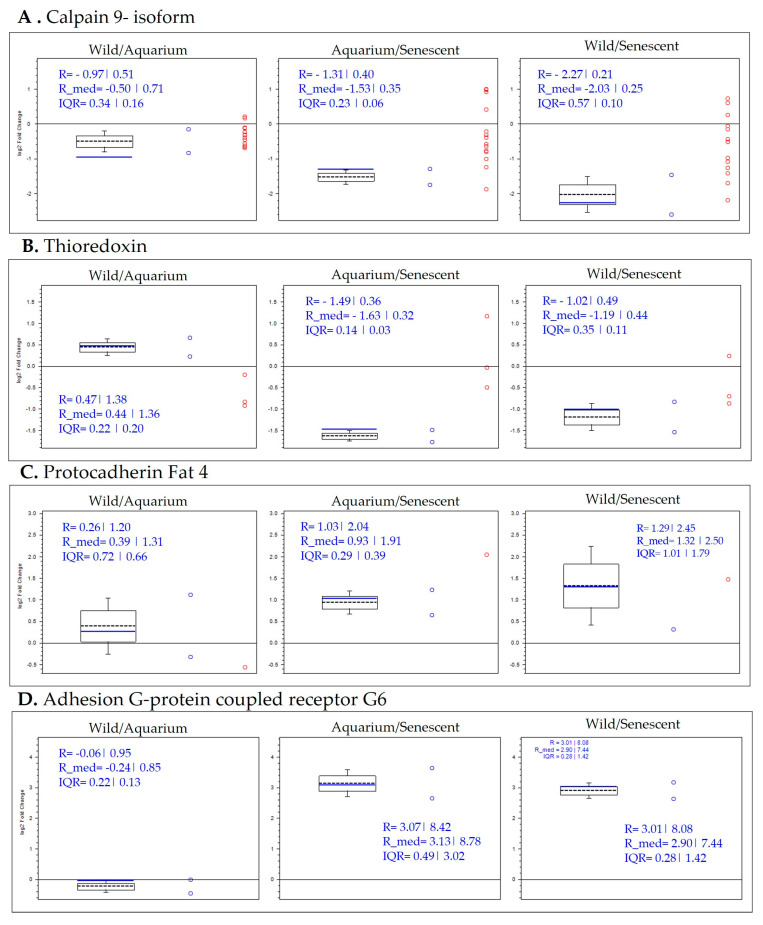
Box plot representations of the quantification ratios of selected protein biomarkers by conditions, more abundant in senescent group (**A**,**B**) and down-regulated in senescent group (**C**,**D**).

A selection of proteins was made from the data obtained in the comparative study by condition. This selection was based on proteins that were highly up-regulated or highly down-regulated under some of the conditions and could be considered potential biomarkers. For a better understanding of the variation in the abundance of selected proteins, a box plot analysis is presented for each protein according to the comparison ratios of the conditions (Supplementary Data S5 and Figure 7 and Figure 8).

Deletions in malignant tumor protein were increased in the wild group versus both the aquarium-maintained and senescent groups (Figure 7A). This result indicates that the increase in this protein is characteristic of the wild group. Similarly, proteins such as caspase3-like, protein mono-ADP-ribosyltransferase and neural cell adhesion were increased in the aquarium-maintained group versus both the wild and senescent groups (Figure 7B,C). This result indicates that the increase in these proteins is characteristic of the aquarium-maintained group, distinguishing it from the wild and senescent groups.

Moreover, calpain-9 and thioredoxin proteins were increased in the senescent group compared to both the wild and aquarium-maintained groups (Figure 8A,B). This result indicates that the increase in these proteins is characteristic of the senescent group. In contrast, adhesion-G-protein and protocadherin 4 were decreased in the senescent group compared to the wild and aquarium-maintained groups (Figure 8C,D). The decrease in these proteins is characteristic of the senescent group.

#### 2.4.2. Time–Course Specimen Analysis

**Figure 9:**
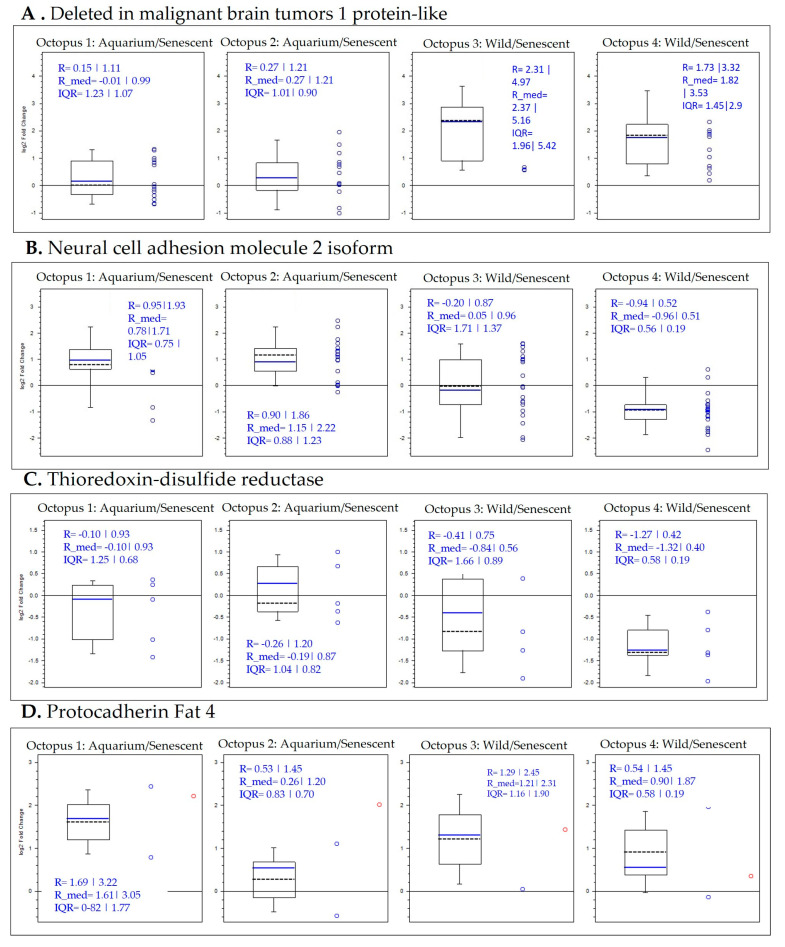
Selected box plot representations of the quantification ratios of selected protein biomarkers by time-course specimen.

The proteins selected in the study by condition have been reviewed based on the data obtained in the time–course analysis. In addition, other proteins with potential as biomarkers have been analyzed, taking into account how the abundance of proteins in the same specimen changes depending on the condition. Results are presented in Figure 9 and Supplementary Data S5.

Deletions in malignant tumor protein was increased in wild samples as can be observed in octopus 3 and 4, and close to no differential abundance in octopus 1 and 2 (Figure 9A). This result indicates that the increase in this protein is characteristic of the wild group, maintaining this pattern in all specimens. Neural cell adhesion protein was increased in aquarium-maintained samples as can be observed in octopus 1 and 2, being close to zero in octopus 3 and slightly abundant in the senescent stage of octopus 4. (Figure 9B). This result indicates that the increase in this protein is characteristic of aquarium-maintained octopus.

In addition, thioredoxin protein was increased in senescent samples as can be observed in octopus 1, 3 and 4 (Figure 9C). Octopus 2 showed slightly higher abundance in the aquarium-maintained stage, although there is close to no difference in abundance across time. This result indicates that the increase in this protein is characteristic of the senescent stage. Protocadherin Fat 4 showed lower abundance in the senescent stage of the four specimens analyzed (Figure 9D). This result indicates that the decrease in this protein is characteristic of the senescent stage.

#### 2.4.3. Preliminary Sex-Specific Study of Senescent Animals

**Figure 10:**
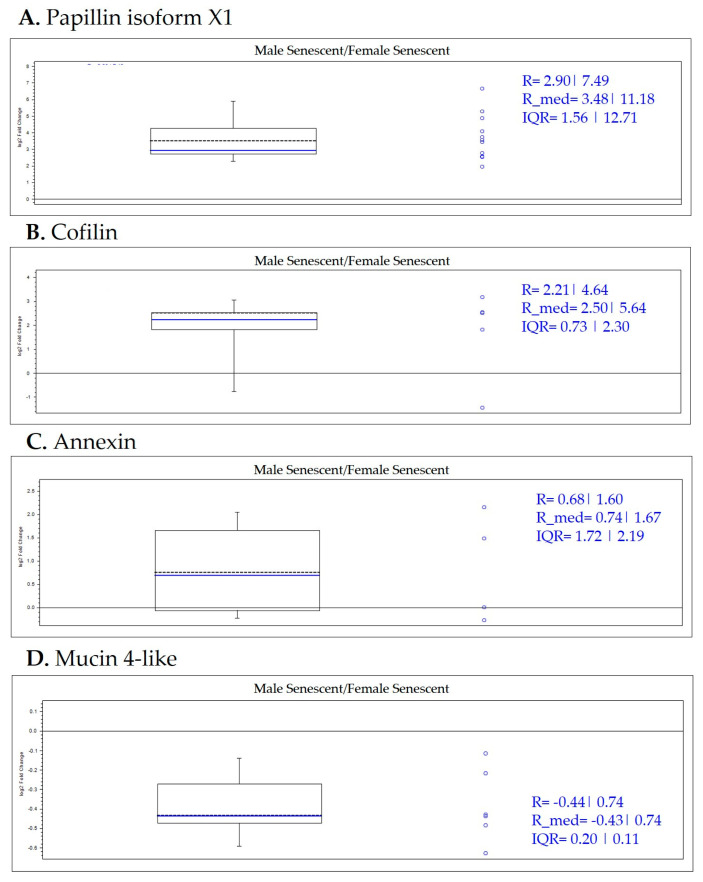
Box plot representations of the quantification ratios of selected protein biomarkers by sex-study of senescent specimens.

From the exploratory data obtained in the sex-specific study of senescent animals, the results are shown in Figure 10 and Supplementary Data S5.

Papilin protein was increased in male senescent samples as can be observed in Figure 10A). This result indicates that the increase of this protein is characteristic of the male group in contrast to female senescent specimens. Cofilin was increased in the male senescent group versus female senescent group (Figure 10B). This result indicates that the increase in this protein is characteristic of the male senescent group. Moreover, annexin was increased in male senescent group as can be observed in Figure 10C. This result preliminary indicates that the increase in this protein is a signature of male senescent octopus. On the other hand, mucin-4-like decreased in the male senescent group versus the female senescent group (Figure 10D). This result indicates that the decrease in this protein is characteristic of the female senescent group. These findings regarding to the sex-related differences need to be confirmed through an additional study that includes a sufficient number of specimens from each sex.

### 2.5. Functional Analysis, Network Analysis, and Potential Bioactive Peptides Identification

Due to the similar protein profiles observed across the studied conditions, where differences were found primarily in their relative abundance rather than in qualitative aspects, the functional analysis, network analysis, and potential bioactive peptide identification were conducted using the entire set of analyzed proteins.

#### 2.5.1. Functional Analysis: PANTHER and DAVID Analysis

**Figure 11:**
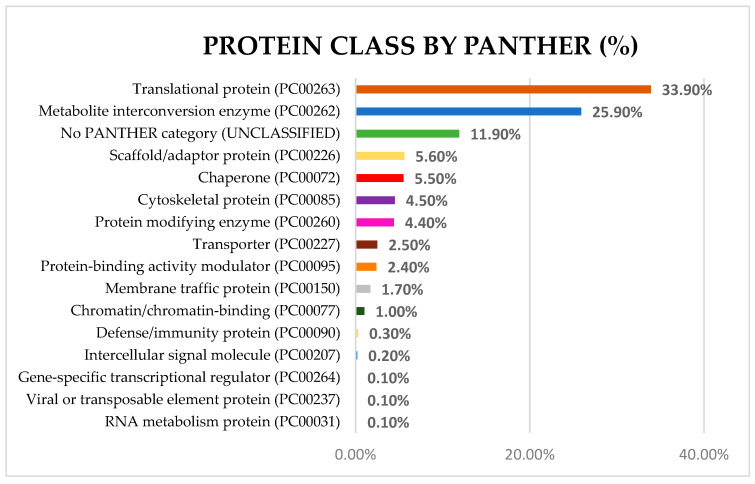
Protein Class of common octopus skin mucus proteome categorized by PANTHER using the gene ID (https://pantherdb.org/, accessed on 20 May 2024).

PANTHER analysis was performed using the gene names (considering all non-redundant proteins), which detected the presence of 16 different protein classes (Figure 11) in common octopus skin mucus proteome.

In this protein classification, the most abundant classes were translational proteins (33.90%), metabolite interconversion enzymes (25.90%), scaffold/adaptor proteins (5.60%), chaperone (5.50%), and cytoskeletal proteins (4.50%).

Within the translational proteins group, 40S ribosomal proteins, and 30S ribosomal proteins were found. Within the metabolite interconversion enzyme class, glyceraldehyde-3-phosphate dehydrogenase, thioredoxin, glutathione S-transferase, and peroxiredoxin-5 were identified. In the scaffold/adaptor protein class tyrosine 3, endophilin-B1, and beta-arrestin-2 were observed. In the chaperone class chaperone protein DnaK, heat shock protein 70, peptidyl-prolyl cis-trans isomerase, and heat shock protein 90 were found. Within the cytoskeletal protein class actin-related protein 2, different tubulin proteins, and putative actin-related proteins were identified.

The defense/immunity protein category was observed in a small percentage (0.30%), but it is important in the function of skin mucus. Within this category, peptidoglycan-recognition protein SC2, and metallothionein-1A protein were included, among others.

The KEGG results (Table 1) showed proteins involved in the amino peptide synthesis (ribosome), proteasome, and endocytosis pathways.

The study of the functional domains by InterPro (Supplementary Data S6) highlighted the functional domains that corresponded to the Glycoside hydrolase domain, Lamin tail domain, If rod domain, NAD(P)-bd domain, VWF domain, and calycin domain. Other important domains were observed with a lower *p*-value, such as the Heat shock protein 70, Hsp 20 like chaperones proteins domain, Immunoglobulin E-set and Immunoglobulin-like domain. Table 2 summarizes the most relevant results from Supplementary Data S6.

#### 2.5.2. Network Analysis

**Figure 12:**
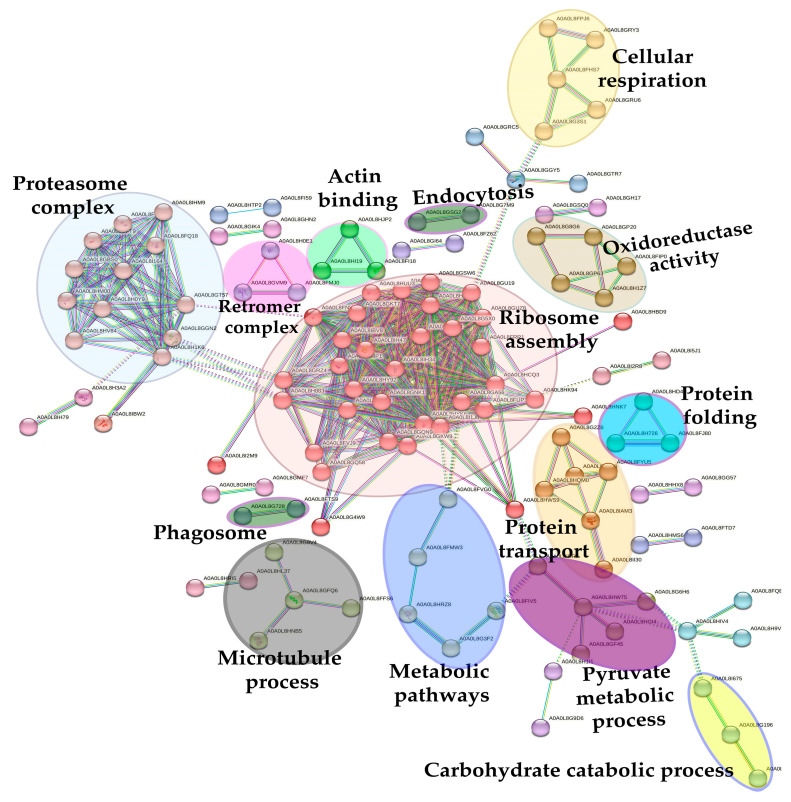
Protein network of common octopus skin mucus proteome by STRING software (v.12.0) (accessed on 22 May 2024). Nodes depict the proteins and the interactions between proteins are represented with continuous lines (physical, direct interactions) and dotted lines (functional, indirect interactions).

A network analysis was performed using the STRING software (v.12.0) (https://string-db.org/, accessed on 23 May 2024) by analyzing all the proteins identified in this study and comparing them with the genome of *Octopus* spp., as it was considered the genetically closest group. The results obtained with an MCL = 2 inflation clustering are shown in Figure 12, where 243 nodes (proteins) and 510 interactions (edges) are displayed. The cluster analysis revealed 27 significant clusters of interactions between nodes, with the most interesting ones highlighted in the figure below.

The most relevant sub-networks are involved in ribosome assembly (in red, 35 nodes), proteasome complex (in grayish blue, 13 nodes), protein transport (in orange, 6 nodes), cellular respiration (in cream, 5 nodes), oxidoreductase activity (in brown, 5 nodes), metabolic pathways (in blue, 5 nodes), pyruvate metabolism process (in violet, 5 nodes), in microtubule process (in gray, 4 nodes), actin binding (in green, 3 nodes), protein folding (in sky blue, 3 nodes), retromer complex (in pink, 3 nodes), carbohydrate catabolic process (in yellow, 3 nodes) and phagosome and endocytosis activity (in dark green, 2 nodes each).

#### 2.5.3. Potential Bioactive Peptides Identification

The prediction of bioactive peptides was performed by in silico enzymatic digestion (pepsin and trypsin) with a minimum of six residues per peptide and no missed cleavages. This process released a total of 39,606 peptides with pepsin digestion (6–44 amino acid residues) and a total of 65,063 peptides from trypsin digestion (6–44 amino acid residues) (Supplementary Data S7). These results were classified by the software PeptideRanker (http://distilldeep.ucd.ie/PeptideRanker/, accessed on 23 May 2024) using the N-to-1 neural network probability [30] to predict peptides that may be more bioactive, and peptides with a score greater than 0.9 were chosen as potential bioactive peptides (Supplementary Data S7), resulting in the selection of a total of 78 pepsin peptides (6–27 amino acid residues) and a total of 620 trypsin peptides (6–38 amino acid residues) (Supplementary Data S8).

The pepsin-derived potential bioactive peptides were primarily associated with mucins (4 and 19), collagen proteins, titins, MAM and LDL-receptors, serine proteases, universal stress protein in QAH/OAS sulfhydrylase 3′region, tyrosinase copper-binding domains, heat shock cognate 71 kDa protein, vasodilator-stimulated phosphoprotein, serine/threonine-proteins, hemocyanins, caspases, neurexin, SCO-spondin, VWFA domain, metalloendopeptidases, papilin, and different uncharacterized proteins.

The trypsin-derived potential bioactive peptides included mucins, MAM and LDL-receptor class A domain-containing proteins, hemocyanins, neurobeachin proteins, Ras-related protein Rab-7, CD109 antigens, collagen proteins, neural-cadherins, von Willebrand factor A domain-containing protein, tetraspanins, mucins, SCO-spondin, tubulin beta chains, deleted in malignant brain tumors 1 protein-like, GDP-fucose protein O-fucosyltransferase, protocadherins and cadherins, chitinase, transforming growth factor-beta-induced protein ig-h3-like isoform and different uncharacterized proteins.

## 3. Discussion

Non-invasive diagnostic techniques are essential tools for monitoring and studying different aspects of the condition of animals, minimizing the stress and damage generated by their manipulation. These techniques acquire great importance when applied to the use and management of animals maintained in aquariums or aquaculture. Among these techniques, the study of skin mucus plays a key role because it serves as the first line of defense for some animals, as it is in direct contact with the environment, providing valuable information about the health of the animals and their interactions with their environment. Due to the importance of skin mucus in the relationship between the common octopus and its environment, the search and identification of biomarkers of welfare and health through the study of its proteomic profile becomes a powerful and non-invasive tool that can provide information on the state of health of the animals in different conditions. This tool is of particular interest for the detection and monitoring of changes that may occur during their maintenance in aquariums or when the animals reach their state of senescence.

For this reason, a comparative analysis of the proteomes of common octopus skin mucus under different conditions—wild octopuses, aquarium-maintained octopuses, and senescent octopuses—is presented for the first time. This is the largest repository of proteins identified to our knowledge for *O. vulgaris* skin mucus for specific maintained conditions (wild, aquarium-maintained, and senescent). The first comprehensive dataset for proteins and peptides contained in the common octopus skin mucus was obtained in 2023, comprising 510 non-redundant proteins, 5937 PSMs and 2038 different peptides [11]. The results presented in this work (1496 non-redundant protein groups) represent an almost three-fold increase in the number of non-redundant proteins previously identified alongside a significant rise in a higher number of PSMs and distinct peptides. This considerable improvement is attributed to the optimization of the protocols and conditions used in the LC-MS/MS analysis utilizing a new instrument (Orbitrap Eclipse Tribrid) and specifically adapted to the study of skin mucus. Additionally, the inclusion of a third database corresponding to the recently published *O. vulgaris* genome [31] on top of those used in the first study of the octopus skin mucus for the processing of the Mass Spectrometry Data and the improvement of the information for the genus *Octopus* in the tools and databases used for the analyses included in this study have allowed for a broader, deeper and more comprehensive understanding of the skin mucus composition to date. Within the obtained protein profiles, a similar qualitative protein composition was observed between condition samples, with highly similar proteins across all profiles, including a large number of mucins, collagen, and proteins with structural functions. In all cases, a smaller number of proteins with defensive and heat shock functions were observed compared to other functions. These results are in line with expectations since the basic functions of mucus require these structural function proteins and are necessary under the different conditions analyzed.

Despite exhibiting similar protein profiles, comparative analysis of the relative abundance of these proteins under each condition revealed significant differences. Depending on the condition of the animal, certain proteins appear up-regulated or down-regulated. A summary of the most relevant proteins in this comparative analysis, which are discussed below, is shown in Supplementary Data S9.

A high abundance of deleted in malignant brain tumors 1 protein-like (DMBT1) was observed in wild octopuses. This protein is involved in mucosal innate immunity by binding to various pathogens and host molecules [32] as well as in epithelial differentiation, and in the function of chromatophores [33]. The up-regulation of this protein in wild octopuses provides useful information about the higher interaction with the environment and the stronger action against pathogens carried out by octopuses in a natural environment. Based on the functionalities of the protein described here and the analysis of the results obtained in the study of the individual proteins by box plot, DMBT1 is considered one of the most interesting proteins, making its up regulated condition a potential bioindicator of welfare and health in wild octopus.

In aquarium-maintained octopuses, mono-ADP-ribosyltransferase PARP14-like isoform X2 protein, caspase-3-like protein, and transcription factor iws-1 isoform X2 were selected among the proteins with biomarker potential due to their higher abundance in this condition following the volcano plot analysis. Caspase-3 acts as an effector enzyme in apoptosis activation [34], serving two critical functions in apoptosis: transmitting the death signal and cleaving various cellular proteins. This cleavage activates or inactivates other proteins, leading to the biochemical and morphological hallmarks of programmed cell death [35]. Apoptosis is an important cellular process in hemocytes, enabling the adequate clearance of damaged, senescent, and infected cells without inflammation [36,37]. Caspase 3 has been described in the common octopus as an effector in apoptosis processes [38]. Transcription factor iws is a transcription elongation factor and ADP-ribosyltransferase mediates mono-ADP-ribosylation of glutamate residues on target proteins. Moreover, neural cell adhesion molecule 2 isoform X13 is more abundant in aquarium-maintained samples compared to wild samples. This protein is involved in cell–cell interactions at synaptic contacts, and mediate binding between cells expressing it, including muscle and glial cells as well as neurons [39,40]. The high abundance of these proteins in aquarium-maintained octopuses suggests a robust response against external agents. This finding implies that aquarium-maintenance practices, as performed in our experiments, likely do not compromise the octopus’s ability to respond to external threats or cause a decline in their defense mechanisms and environmental interactions. Once the functionalities of these potential biomarker proteins for octopus in aquarium-maintained conditions have been analyzed and compared with the results obtained in the box plot analysis, these proteins can be used as specific biomarkers for health and welfare in aquarium-maintenance and could be monitored using Targeted Proteomics approaches. A more in-depth analysis of these proteins would increase and improve the selection to generate a useful health and welfare biomarkers panel.

In the analysis of the proteins selected as potential biomarkers of health two of them were up-regulated in the aquarium-maintained group, but showed a large decrease in the senescent group compared to wild octopuses and aquarium-maintained octopuses. These proteins are beta-N-acetylhexosaminidase and adhesion G-protein coupled receptor G6 proteins. These proteins assist in the passage of materials through a tract. G protein-coupled receptors are essential for organisms to perceive and respond to their environments. They also play a critical role in maintaining homeostasis through endocrine and neuronal functions [41]. A decrease of these proteins in the senescent stage may lead to a deterioration of endocrine and neuronal functions in octopuses. Furthermore, in senescent octopuses, a significant increase in the abundance of numerous proteins compared to specimens maintained in aquariums or wild specimens was observed. Among the up-regulated proteins, the calpain-9 isoform X8 protein and the thioredoxin protein were identified. These proteins are closely related to oxidative stress, and their abundance increases when oxidative stress is elevated.

Some proteins are more abundant in aquarium-mantained and wild samples when these profiles are compared to the senescent samples, indicating that these proteins are expressed in lower abundance in the senescent stage. These proteins include Mucin-like protein, Mucin-19-like, Protocadherin Fat 4, Tetraspanin, and Tetraspanin D107. Mucins are important in mediating the interactions between the cell and their external environment [11,42], which effectively protects the body from infection, dehydration, and physical or chemical injury, as well as helping the passage of materials through a tract. A decrease in the abundance of mucins in senescent octopuses could result in a loss of the natural consistency of the skin mucus, which might impair its efficiency in protecting against external agents and physico-chemical alterations in the environment.

Protocadherins, although thought to be an evolutionary event exclusive to vertebrates, play an important role in the cephalopod nervous system, representing a striking case of convergent evolution [43]. In the common octopus, it is probable that they would act as immune related proteins and also cell-adhesion molecules, although nothing is known regarding their adhesion specificity [44]. In the brain of adult octopuses, protocadherins are involved in neuronal plasticity, but the presence of these proteins in non-neural tissues has been investigated in the arm tip of the common octopus, where a continuous growth and rewiring of newly developing sensory systems is required. A lower abundance of these proteins in the skin mucus of senescent octopuses may indicate a decrease in the regenerative function of the animal and slower development of the sensory system as well as the reduction in the immune response capability. Tetraspanin and Tetraspanin D107 have been found in common octopus ink, but their role is not yet well understood [45]. These proteins are involved in cell-cell adhesion at cellular junctions and are considered molecular facilitators or organisers at the plasma membrane [46]. The reduced abundance of these proteins in the skin mucus of the common octopus in the senescent stage provides important information on the state of degradation associated with this stage and may provide a useful biomarker of health status to identify senescence in octopuses.

The time–course individual-level analysis for two conditions (Aquarium-maintained vs. Senescent and Wild vs. Senescent) revealed trends similar to the condition-level results. The high abundance of deleted in malignant tumor in wild animals and the high abundance of proteins such as neural cell adhesion in aquarium-maintained animals support the results obtained in the study by condition. In addition, in proteins related to senescent stages, in both, proteins with increased abundance (thioredoxin) and proteins with decreased abundance (protocadherins and tetraspanin) in this stage, a similar pattern to the results obtained by condition is also observed. The time–course individual-level analysis for two conditions (Aquarium-maintained vs. Senescent and Wild vs. Senescent) revealed similar trends. All these proteins are proposed to be used as biomarkers for for early detection of senescence in animals kept in aquarium conditions or aquaculture systems.

The onset of the senescent stage is an important event in the life cycle of the octopus, as it is associated with major physical and behavioral changes. Apart from general changes that occur in all individuals, senescence differs markedly between males and females. A preliminary study was conducted to examine the potential effects of these gender differences on the protein profiles, despite the relatively small sample size, given the great importance of these gender differences and the potential value of the obtained results in laying the foundation for a new and significant research line. In this preliminary analysis, the results obtained from comparing the proteomic profile in senescent sex-specific specimens suggest the presence of many differential proteins, which could explain some of the sex-based differences observed in this species. In male octopus the results of the present article show that papilin protein levels are increased compared to female senescent animals. Papilin is involved in the development and maintenance of reproductive tissues [47]. Therefore, papilin protein could be considered a biomarker for the functionality of reproductive success in male octopuses. In addition, cofilin protein levels are increased in male senescent octopus. Confilin plays a role in regulating the actin filaments within the hectocotylus, ensuring efficient and effective transfer of spermatophores. Furthermore, cofilin has the remarkable ability to regenerate lost arms. This process involves extensive cellular remodeling and growth, where actin dynamics play a crucial role. Cofilin, by regulating actin filament disassembly and assembly, may be vital in the wound healing and regeneration processes in male octopuses. During senescence, significant changes occur in the cytoskeletal architecture of cells. Cofilin-mediated actin dynamics play a crucial role in these changes, influencing cell shape, motility, and division. In senescent cells, dysregulation of actin dynamics can lead to impaired cellular functions and increased susceptibility to apoptosis [48]. Annexin also showed increased abundance in male senescent octopus compared to female senescent octopus. This protein plays significant roles in cellular processes that are crucial during the senescence of male octopuses. Its involvement in membrane repair, signal transduction, cytoskeletal interactions, and apoptosis highlights its potential impact on the physiological changes observed during this lifecycle stage [49]. The rapid degeneration of tissues, including muscles and neurons, underscores the importance of annexins in maintaining cellular integrity and function despite the overall decline. Further research into the specific roles and regulatory mechanisms of annexins during octopus senescence could provide deeper insights into the molecular underpinnings of aging and cellular dynamics in these fascinating cephalopods. In contrast, mucin-4-like protein showed increased abundance in female senescent octopus compared to male senescent octopus. Mucin-4-like protein likely plays significant roles in the senescence of female octopuses through its involvement in cellular protection, immune response, apoptosis regulation, and tissue integrity [50]. The rapid degeneration of tissues, including muscles and epithelial surfaces, underscores the importance of mucin-4-like proteins in maintaining cellular integrity and function during the post-reproductive decline. Further research into the specific roles and regulatory mechanisms of mucin4-like proteins in octopus senescence could provide deeper insights into the molecular underpinnings of aging and cellular dynamics in cephalopods.

Although all these potential biomarkers need to be validated further, for instance, through targeted proteomics experiments, understanding the mechanisms behind these protein signatures not only enriches our knowledge of octopus biology but also offers broader implications for studying aging and cellular protection in other organisms. Therefore, more in-depth studies are needed to definitively confirm these findings.

Since the protein profiles are qualitatively very similar across the different conditions, for the Functional Analysis, Network Analysis, and Potential Bioactive Peptides Identification a general list of all the proteins found in the analyzed skin mucus has been used, without making distinctions by conditions. When comparing the results obtained in the Functional Analysis, Network Analysis, and Potential Bioactive Peptides Identification in this work with those from previous studies [11], it was observed that the analyses conducted using PANTHER and DAVID yielded very similar outcomes. This similarity is attributed to the fact that, despite the considerable increase in the number of identified proteins, all of them belong to protein classes similar to those found in previous studies and are involved in the same pathways or similar processes. However, differences were noted in the results obtained from the network analysis using STRING when compared to the initial characterization of the skin mucus proteome. The primary reason for these differences lies in the choice of reference organisms used for the analysis. In the previous study by Pérez-Polo et al. 2023 [11], both the genus *Octopus* and the species *Homo sapiens* were included as references, while in the present study the comparative analysis exclusively considered the genus *Octopus*. This decision was made due to the increased number of proteins obtained and the improvements in the databases, which allowed for more accurate comparative results reflecting the interactions specific to this species. Despite the differences observed in the clusters presented, the results are still closely associated with activities related to the formation of proteins, their interaction with each other and with DNA, processes of formation and destruction of cellular components, and in pathways related to defense and recognition of external factors.

The results from the Potential Bioactive Peptides Identification study significantly expand the number of proteins harboring Peptides with antimicrobial activity. Consistent with the observations of Pérez-Polo et al. 2023 [11], the results reveal that mucins, collagen proteins, titins, hemocyanins, MAM and LDL-receptors, SCO-spondin, and heat shock cognate 71 kDa parent proteins exhibit the highest potential for antimicrobial activity. Notably, the study also identifies novel proteins with significant potential, including transforming growth factor-beta-induced protein ig-h3-like isoform, neurobeachin proteins, Ras-related protein Rab-7, CD109 antigens, protocadherins, and cadherins and serine proteases. These proteins play crucial roles in both cell adhesion and defense against external agents [42,51]. Antimicrobial activity represents an added value to the functionality of these proteins, as their breakdown due to external or chemical stress can release the peptides and contribute to organismal defense.

Finally, in all the analyses carried out in the present study, a multitude of uncharacterized proteins were found. These proteins also show high potential for bioactive peptides and antimicrobial activity, among others. One of the reasons for this absence of information is that, even though the genome of the common octopus has been recently published [31] the protein reference databases still require improvement in the annotations and the information contained within them. The lack of information on these proteins highlights the need for further research to better characterise the skin mucus proteome, to better understand the proteins involved, and to improve our understanding of its functionality in the octopus. Furthermore, this will enable better knowledge of the significant applications that this non-invasive analysis method can provide for pharmacology as well as for control and recognition of the octopus health status.

## 4. Materials and Methods

### 4.1. Animal Capture and Maintenance

A total of 6 specimens of *O. vulgaris* with an average weight of 1 kg (980–1400 g) were collected using fishing cages by professional certificated fishermen at the Ría de Vigo, Spain (2414.090 N, 847.180 W) and were transported in containers to the Experimental Culture Facilities of IIM-CSIC, an institution registered as a “User and breeding center on animal experimentation” ES360570202001. Procedures for transportation and maintenance were carried out following the principles of animal welfare and the European Directive 2010/63/EU. Special attention was paid to the 3Rs strategy (Reduce, Refine, Reuse), reducing the number of animals used in the experimental assay until the essential for achieving statistical significance. For this reason, the usage of a reduced number of specimens (six) was decided in order to grant biological replications of the results without the need to use a larger number of specimens, maintaining the compromises of good procedures with experimental animals. The experiments were also approved by the Ethic Committee of the Competent Authority (ES360570202001/24/EDUC. FORM07/CGM01). During the experiment, specimens were maintained individually in tanks of 500 L of filtered aerated seawater with a continuous re-circulating flow. The water temperature was set at around 15 ± 1 °C and the photoperiod was 12 h light:12 h dark. Food, consisting of frozen fish and mussels, was also supplied daily, and cleaning and water parameters were checked daily.

Of the six specimens studied, two of them (Octopus 3 and 4) were sampled after 3 days of acclimatization in the aquarium, forming the group of wild octopuses, together with Octopus 5 which was sampled upon arrival at the facilities, without acclimatization period (3W, 4W, 5W). The remaining two octopus (Octopus 1 and 2) were sampled for the first time when they had been kept in aquariums for three months, forming the group of aquarium-maintained octopuses (1A and 2A). Octopuses 1, 2, 3 and 4 were sampled again after several months, when they reached the senescent stage and, together with octopus 6, which was only sampled in its senescent stage, form the group of senescent octopuses (1S, 2S, 3S, 4S, 6S).

### 4.2. Skin Mucus Collection and Protein Extraction

Skin mucus samples were collected from octopus specimens using the method of Vizcaino et al. (2023) [12]. In this method, the skin mucus was collected from each animal after 10 min of arms exposure to the air, out of the seawater, by gently squeezing and scraping the skin surface of each arm using a cell scraper manually. The samples were vigorously shaken and stored at −80 °C until use. Sampling procedures were performed under anesthetic conditions using a mix of ethanol C_2_H_5_OH (1%) and MgCl_2_ (0.011 g mL⁻¹) dissolved in seawater to reduce distress [52].

Proteins were extracted from 1 mL of *O. vulgaris* skin mucus per sample using 3 mL of lysis buffer (10 mM Tris-HCl pH 7.2 with 5 mM of phenylmethylsulfonyl fluoride (PMSF)). Each sample was homogenized on ice using a vortex and was centrifuged at 40,000 g for 20 min at 4 °C in a centrifuge (Avanti JXN-26, Beckman Coulter, Palo Alto, CA, USA). The proteins, present in the supernatant, were quantified using the bicinchoninic acid protein assay (Sigma Chemical Co., St. Louis, MO, US). The supernatant proteins were recovered and stored at −80 °C until used.

### 4.3. SDS-Polyacrylamide Gel Electrophoresis (SDS-PAGE)

Skin mucus proteins were separated on 12% (*v*/*v*) polyacrylamide gels (acrylamide/N, N0-ethylene-bis-acrylamide, 200:1). A total of 20 µg of proteins in Laemmli buffer was boiled for 5 min at 95 °C and centrifuged at 10,000× *g* for 2 min at 4 °C. A PageRuler unstained protein ladder was also used as the molecular weight (MW) indicator (Thermo Fisher Scientific, San Jose, CA, USA). The samples were separated per well in a Mini-PROTEAN 3 cell (Bio-Rad, Hercules, CA, USA) and running conditions were 80 V for the first 20 min and then 150 V until the end of the electrophoresis. Gels obtained were stained overnight with Coomassie Brilliant Blue R-250 (VWR International, Radnor, PA, USA), unstained by using a solution composed of 25% ethanol and 8% acetic acid, washed with 50% methanol (*v*/*v*), and scanned at 200 dpi.

### 4.4. In-Solution Protein Digestion with Trypsin and TMT Differential Labeling

A total of 100 µg of skin mucus protein extract were digested with trypsin as described by Carrera et al. (2013) [53]. Samples were denatured in 8 M urea with 25 mM ammonium bicarbonate pH 8 and reduced with 9 mM DTT (DL-dithiothreitol) 98% (HPLC) (Roche, Mannheim, Germany, EU) for 45 min at 56 °C. The samples were alkylated with 50 mM iodoacetamide (Thermo Fisher Scientific) for 60 min at room temperature in the dark. After alkylation samples were diluted 4-fold with 25 mM ammonium bicarbonate pH 8.00 to decrease the urea concentration. Proteins were finally digested with trypsin (Promega, Madison, WI, USA) (1:100 protease-to-protein ratio) overnight at 37 °C and the reaction was stopped with 5% formic acid (FA) until pH 2.00.

For a quantification approach of the protein profile of the mucus, TMT 10-plex labeling was used (Thermo Fisher Scientific), adapting the method described by Stryiński et al. (2019) [54]. Digested samples were lyophilized and resuspended with 100 μL of 100 mM TEAB buffer and 41 μL of reconstituted TMT labeling reagent were added to each sample and incubated for 1 h at room temperature. Each sample was labeled specifically with a different TMT tag or label (Table 3). To quench the reaction, 8 μL of 5% hydroxylamine were added to each sample and incubated for 15 min. Samples were combined in a new tube in equal amounts. TMT-labeled peptide concentration was measured using the Pierce Quantitative Colorimetric Peptide Assay (Thermo Fisher Scientific) and purified for MS analysis using C18 MicroSpin™ columns (The Nest Group, South-borough, MA, USA).

### 4.5. LC-MS/MS Analysis

Samples were acidified with formic acid (5%) and analyzed by liquid chromato–raphy–tandem mass spectrometry (LC-MS/MS) using a Vanquish™ Neo UHPLC system (Thermo Fisher Scientific, San Jose, CA, USA) coupled to an Orbitrap Eclipse Tribrid mass spectrometer (Thermo Fisher Scientific) equipped with a field asymmetric ion mobility spectrometry (FAIMS) Pro unit. Peptides (900 ng) were separated by reversed-phase chromatography using a C18 column (EASY-Spray column, 25 cm, 75 µm ID, PepMap C18, 2 µm particles, 100 Å pore size, Thermo Fisher Scientific) with a 10-mm pre-column (Accucore XL C18, Thermo Fisher Scientific) using 0.1% formic acid (mobile phase A) and 80% acetonitrile (80% ACN) with 0.1% formic acid (mobile phase B). A 120 min linear gradient from 1 to 45% B, at a flow rate of 300 nL min⁻¹ was used. A spray voltage of 1.95 kV and a capillary temperature of 230 °C were used for ionization. The peptides were analyzed in positive mode (1 scan; 400–1600 amu), followed by 10 data-dependent higher energy collision dissociation (HCD) MS/MS scans (1 μscan), using a normalized collision energy of 38% and an isolation width of 3 amu. FAIMS compensation voltages (C.V.) alternated between −40, −60, and −80 V within a single run, with a 1 s cycle for each experiment. Dynamic exclusion for 60 s after the second fragmentation event was applied and unassigned charged ions were excluded from the analysis.

### 4.6. Processing of the Mass Spectrometry Data

The mass spectra obtained in the Orbitrap Eclipse Tribrid instrument were compared and validated with theoretical mass spectra by using the *O. vulgaris* longest peptide database containing 29,899 peptide sequence entries and the *O. vulgaris* peptide containing 23,272 peptide sequence entries (15 April 2024) obtained from the newly published *O. vulgaris* genome [31] which offers a high percentage of annotated proteins. In addition, to extend the protein annotation, we used the UniGene transcriptome database of *O. vulgaris* paralarvae [55] containing 77,838 protein sequence entries, and the Cephalopoda protein dataset from the UniProtKB database (including canonical and isoformic sequences) containing 230,546 protein sequence entries. The software used for the analysis was SEQUEST-HT (Protein Discoverer 2.4, Thermo Fisher Scientific), with the following restrictions: 10 ppm tolerance for original ions, 0.06 Da for MS/MS fragment ions, and tryptic cleavage with up to 2 possible missed cleavage sites and tolerances of 10 ppm for parent ions and 0.06 Da for MS/MS fragment ions. TMT labeling (+229.163 Da on N-termini and lysine residues) and carbamidomethylation of cysteine (+57.021 Da) were set as fixed modifications. The permissible variable modifications were methionine oxidation (+15.994 Da), acetylation (+42.011 Da) of the N terminus of the protein, and deamidation (+0.984 Da) of asparagine and glutamine. Results were subjected to statistical analysis with the Percolator algorithm to keep the false discovery rate (FDR) below 1%, which represents the proportion of false peptide spectrum matches (PSMs) among all accepted PSMs. To reduce protein redundancy in the protein list from multiple database searches, protein groups generated directly from the Protein Discoverer 2.4 analysis were selected. Additionally, master proteins were identified, and entries with zero unique peptides were removed.

### 4.7. Statistical Analysis: Euclidean Hierarchical Clustering, PCA Analysis, Volcano Plot and Box Plot Analysis

Relative quantification was performed using the Quantification Node and normalization against the total peptide amount (Proteome Discoverer 2.4 package, Thermo Fisher Scientific). After relative quantification, several filters were applied to obtain the final list of differentially regulated proteins (DRPs): (a) proteins classified as master proteins, (b) at least a 1-fold change (FC ≥ 1.0) in normalized ratios, (c) 1-way ANOVA on ranks and a (*p*-value ≤ 0.05). The function heatmap.2 of the statistical package R version (v) 4.4.0 (https://www.r-project.org/, accessed on 15 April 2024) was used to achieve the Euclidean hierarchical clustering of the data. The ggplot2 v.4.1.1 package, the Euclidean distance metric plus the complete linkage for the agglomeration method were performed as constraints. PCA analysis was determined using normalized abundances of the different group conditions by the Proteome Discoverer 2.4 package (Thermo Fisher Scientific). Volcano plots defined the significant protein abundance differences by up and down regulating proteins. For saying that a protein is up or down-regulated, some parameters have to be accomplished. For up-regulated proteins, fold-change (FC) > 1.0 and (*p* < 0.05) parameters are needed, and for down-regulated proteins FC < −1.0 and (*p* < 0.05) parameters are also needed. Fold-regulated parameters correspond to the Log2 Ratio (x-axis) and the *p*-value corresponds to −Log10 *p*-value (y-axis). The final list of modulated proteins was compared by box plot analysis according to the ratios of the conditions and taking the log2 Fold Change as the analysis value using the Proteome Discoverer 2.4 package (Thermo Fisher Scientific).

### 4.8. Functional Gene Ontologies and Pathways Analysis

For the classification of proteins, the final list of non-redundant protein Gene IDs was submitted to the PANTHER program version 18.0 (http://www.pantherdb.org, accessed on 20 April 2024). The classification of proteins was based on Protein Class. The KEGG pathway analysis was performed by comparing the input data with the background of the different species (*Octopus vulgaris*, *O. bimaculoides*, *O. sinensis*, *Lottia gigantea*, and *Crassotrea gigas*) genome by DAVID (https://david.ncifcrf.gov/home.jsp, accessed on 22 May 2024) [56]. The functional domains by InterPro Motifs were obtained by comparing the input data with the background of the *O. vulgaris*, *O. bimaculoides*, *O. sinensis*, *Lottia gigantea*, and *Crassostrea gigas* genomes using DAVID software.

### 4.9. Network Analysis

Network analysis was performed with the STRING (Search Tool for the Retrieval of Interacting Genes) software (v.12.0) (https://string-db.org/, accessed on 22 May 2024) [57]. To minimize both false positives and false negatives, all interactions were selected for analysis as “high confidence” (<0.7) in the STRING software (v.12.0). Cluster networks were created using the MCL (Markov Cluster Algorithm) inflation algorithm, which is included in the STRING website and a value of 2 was selected for all the analyses.

### 4.10. Bioactive Peptides Prediction

For the study of bioactive peptides encoded in the common octopus skin mucus proteome, the MS-Digest software (https://prospector.ucsf.edu/prospector/cgi-bin/msform.cgi?form=msdigest, accessed on 23 May 2024), which is included in the ProteinProspector v.6.5.0 website, was used (https://prospector.ucsf.edu/prospector/mshome.htm, accessed on 23 May 2024). In-silico protein hydrolysates using pepsin and trypsin enzymes were analyzed and all peptides were ranked using the PeptideRanker software (https://prospector.ucsf.edu/prospector/mshome.htm, accessed on 23 May 2024) using the N-to-1 neural network probability to predict the potential bioactivity of peptides [31]. Selected peptides with a score of >0.9 in PeptideRanker were compared with the BIOPEP-UWM database (http://www.uwm.edu.pl/biochemia/index.php/pl/biopep/, accessed on 25 May 2024) [58] and CAMP database (http://www.bicnirrh.res.in/antimicrobial/, accessed on 25 April 2024) [59] applying the DAC score (Discriminate Analysis Classifier score) to predict all potential bioactive peptides.

## 5. Conclusions

The results obtained revealed significant variations in the relative abundance of specific proteins across three conditions in the common octopus (wild, aquarium-maintained, and senescent groups). Several proteins emerged as potential biomarkers of welfare and health to be used in aquarium maintained or aquaculture systems. Notably, despite observed differences in the protein profile of aquarium-maintained octopuses compared to wild octopuses, their response to external threats and the role of mucus in environmental interactions appear unaffected. Conversely, elevated levels of proteins associated with potential health decline (calpain-9, thioredoxin) were observed in senescent octopuses. This study further expands knowledge regarding potential bioactive peptides within the common octopus’s skin mucus, where uncharacterized proteins remain a significant portion. Among the activities identified, antimicrobial activities were highlighted.

## Figures and Tables

**Table 1:** KEGG pathway analysis of the common octopus skin mucus proteome by DAVID (https://david.ncifcrf.gov/home.jsp, accessed on 22 May 2024).

KEGG Pathway	*p*-Value
Proteasome	8.40 × 10^−2^
Ribosome	6.80 × 10^−2^
Endocytosis	3.54 × 10^−2^

**Table 2:** Functional InterPro motifs analysis of the common octopus skin mucus proteome by DAVID (https://david.ncifcrf.gov/home.jsp, accessed on 22 May 2024).

InterPro Motifs	Count	%	*p*-Value
Glycoside hydrolase	20	1.5	2.74 × $10^{-9}$
Lamin tail domain	9	0.7	1.31 × $10^{-8}$
NAD(P)-bd domain	30	2.3	2.53 × $10^{-8}$
Lamin tail domain	9	0.7	3.25 × $10^{-8}$
VWF domain	13	1.1	1.43 × $10^{-7}$
Calycin	9	0.7	5.15 × $10^{-7}$
IF rod domain	9	0.78	8.88 × $10^{-7}$
Heat shock protein 70	4	0.3	2.83 × $10^{-2}$
HSP20-like chaperone	5	0.4	5.10 × $10^{-2}$
Immunoglobulin E-set	8	0.6	6.53 × $10^{-2}$
Immunoglobulin like domain	11	0.8	9.27 × $10^{-2}$

**Table 3:** Mucus samples, study groups, and TMT labeling. The numbers on the sample designate the octopus specimen. Samples with the same number correspond to the same specimen. W: wild, S: senescent, A: aquarium-maintained.

Sample	TMT Label	Study Group	Sex
5W	126	Wild	Female
3W	127N	Wild	Female
3S	127C	Senescent	Female
4W	128N	Wild	Female
4S	128C	Senescent	Female
1A	129N	Aquarium-maintained	Female
1S	129C	Senescent	Female
2A	130N	Aquarium-maintained	Female
2S	130C	Senescent	Female
6S	131	Senescent	Male

## Data Availability

Data available in a publicly accessible repository. The data presented in this study are openly available in [ProteomeXchange database, (https://www.proteomexchange.org/, accessed on 7 June 2024)] reference number [PXD052915]. The original data presented in the study are also included in the article/Supplementary Material; further inquiries can be directed to the corresponding authors.

## Supplementary Materials

The following supporting information can be downloaded at: https://www.mdpi.com/article/10.3390/ijms25189953/s1.
