# Supplementary material for: Decoding Octopus Skin Mucus: Impact of Aquarium-Maintenance and Senescence on the Proteome Profile of the Common Octopus (Octopus vulgaris)
Source: Int J Mol Sci. 2024 Sep 15;25(18):9953. doi: 10.3390/ijms25189953 (PMC11431876; doi:10.3390/ijms25189953)

Supplementary Data S5

Box plot analysis of the proteins selected as potential biomarkers of health in the study by condition.

Universal stress protein in QAHOAS sulhydrate 3 region isoform X2

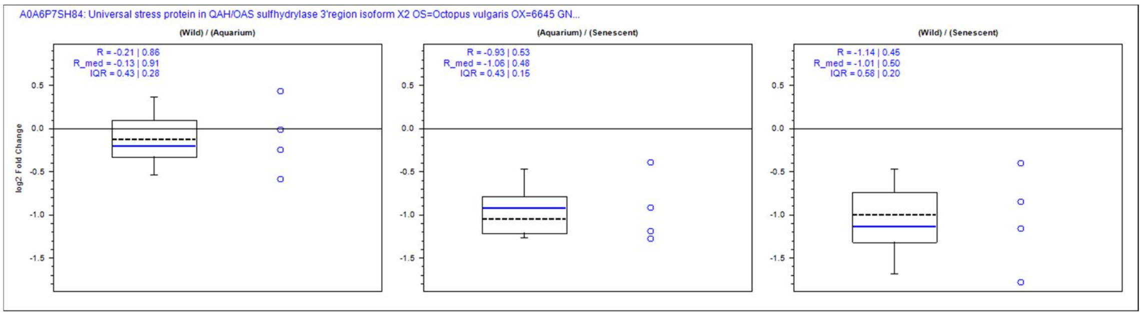

Acidic mammalian chitinase

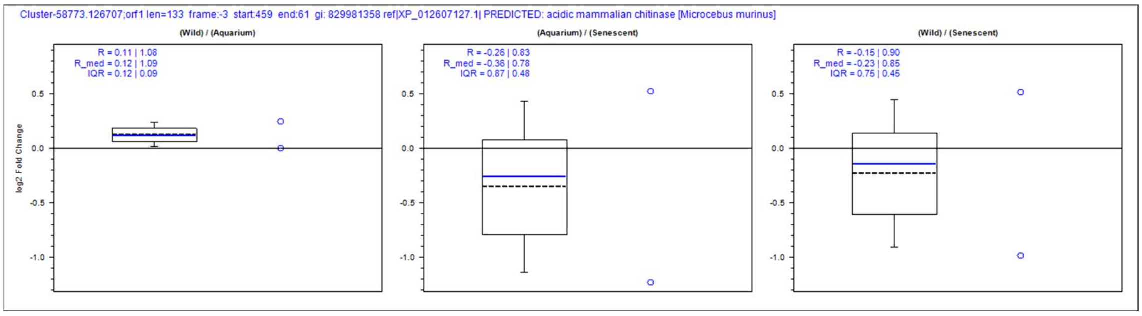

ACTB_G1

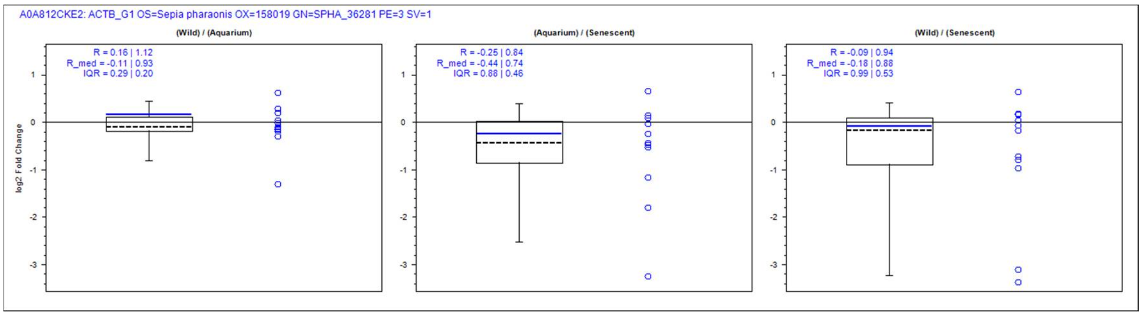

Adhesion G-protein coupled receptor G6

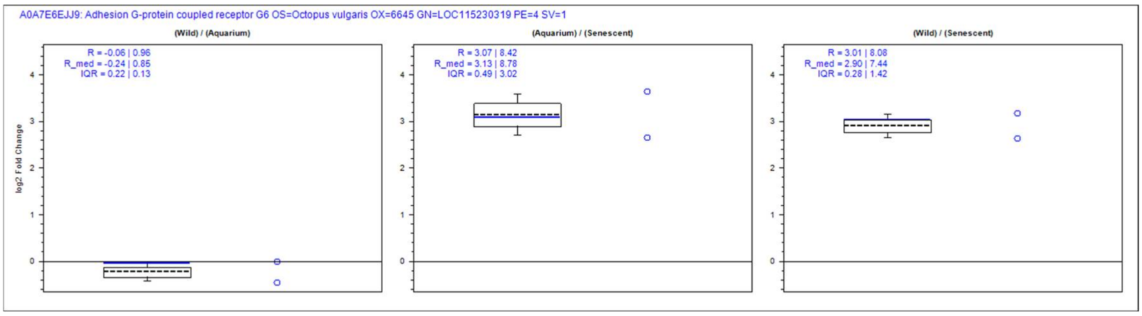

Beta-N- acetylhexosaminidase

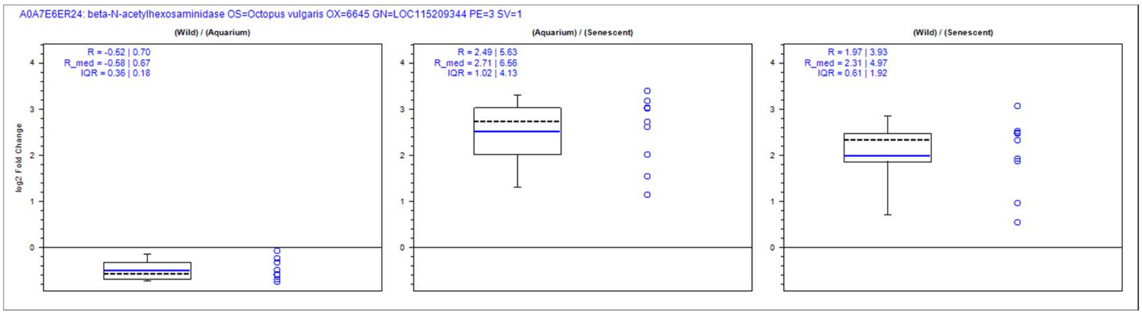

Calpain-9 isoform x8

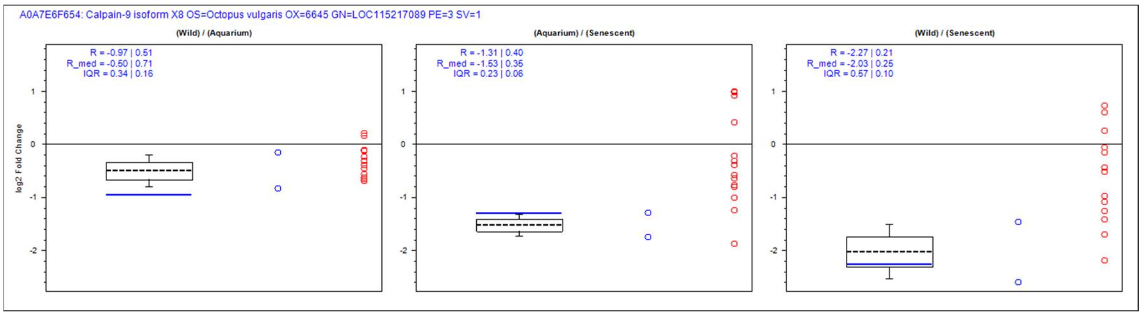

Caspase-3-like isoform X5

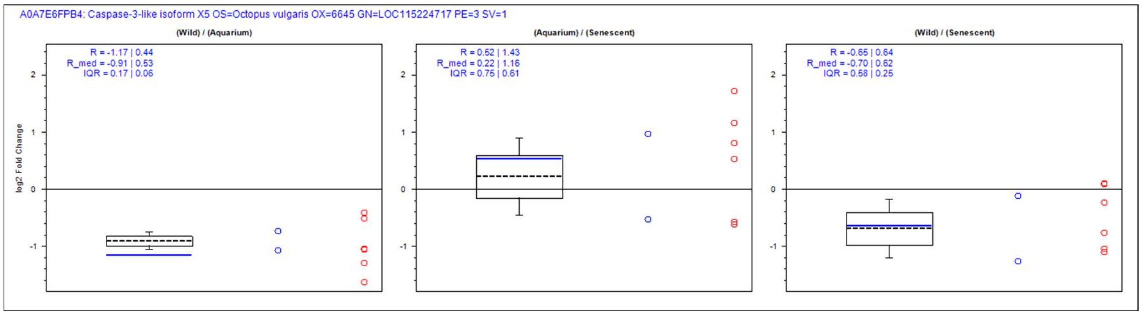

Caspase-7-like

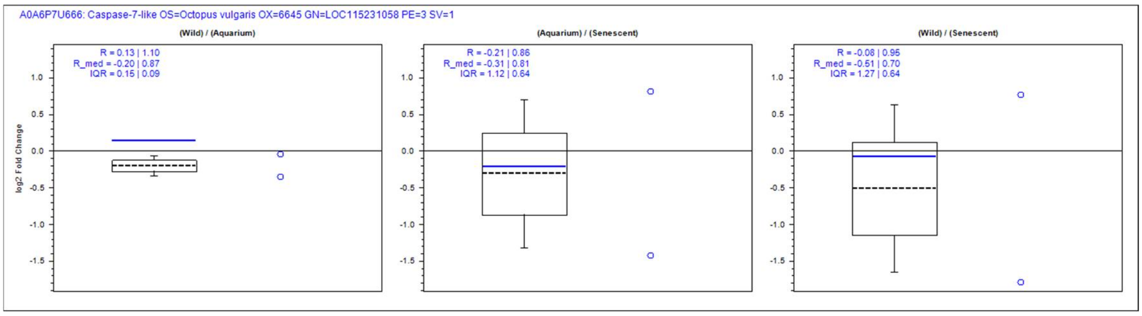

Deleted in malignant tumor

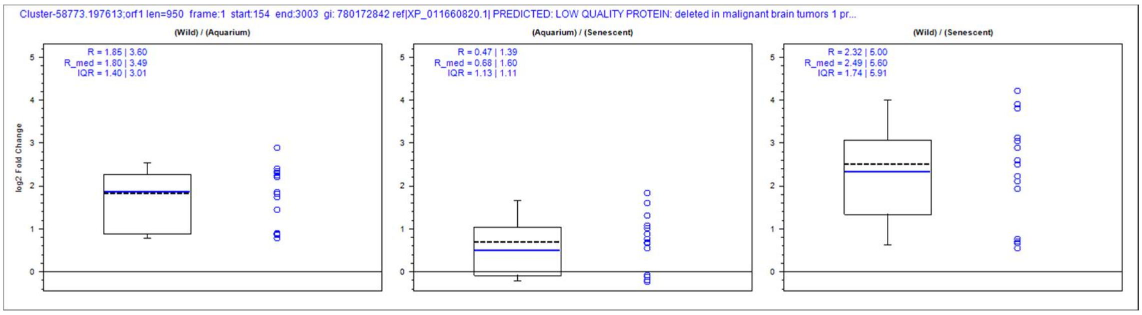

Golgi- associated plant pathogenesis-related protein 1-like

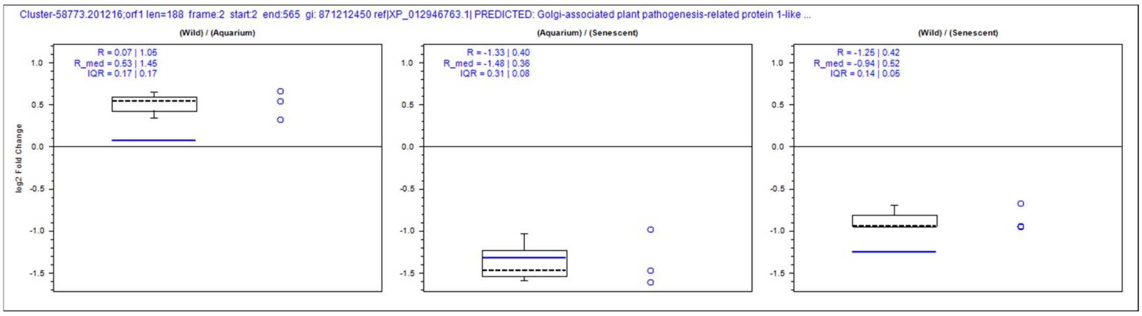

Histone H-1 delta

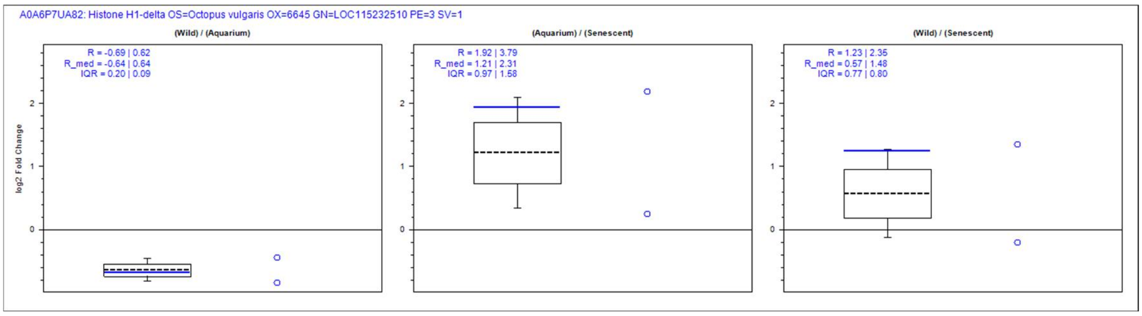

Ldt-domain-containing protein

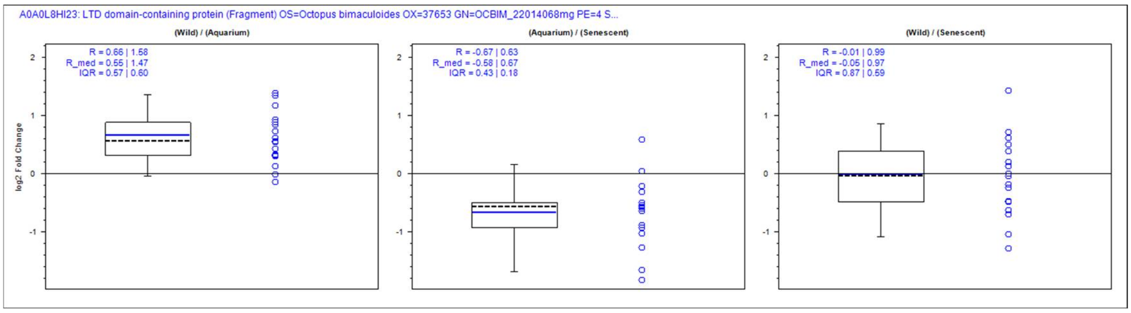

MAM and LDL-receptor class A domain-containing protein 2-like

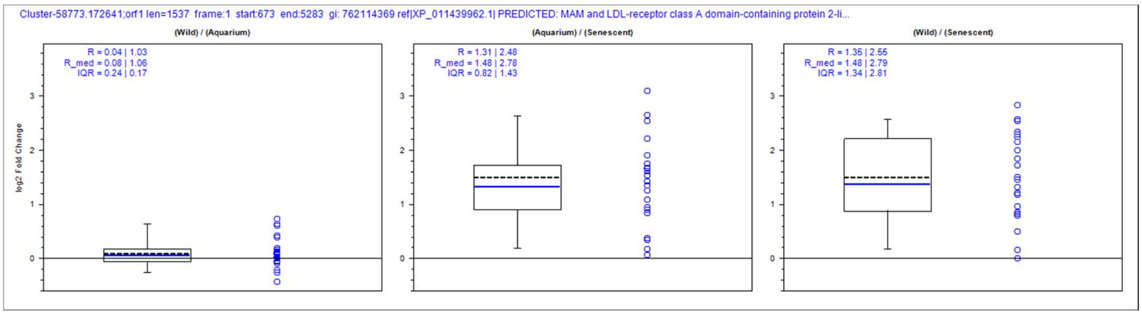

Protein mono-ADP-ribosyltransferase PARP14-like isoform X2

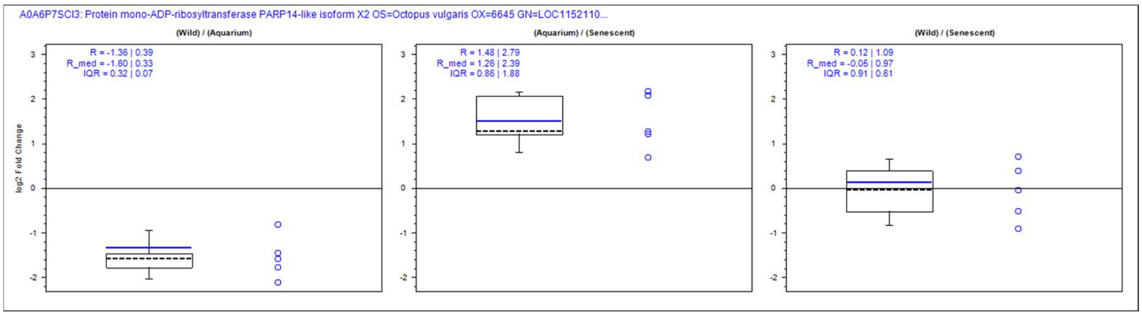

Mucin-4-like

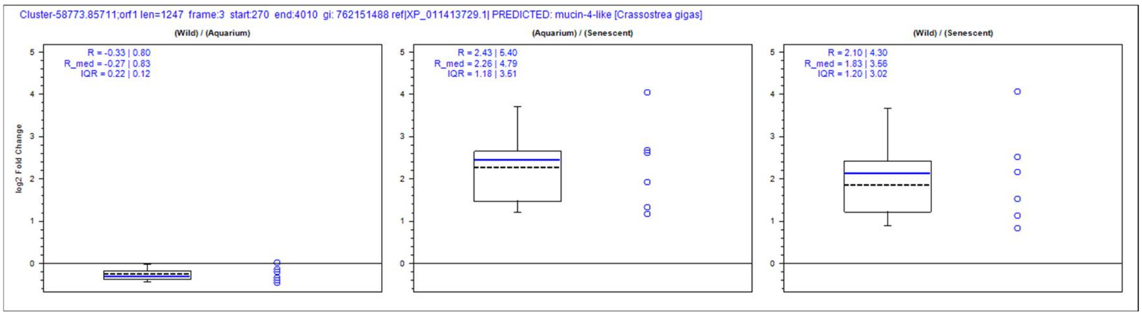

Mucin-19-like

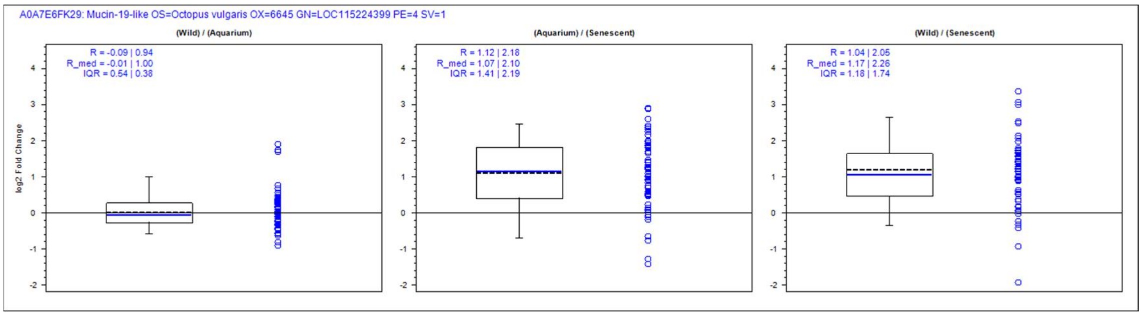

Mucin-like-protein

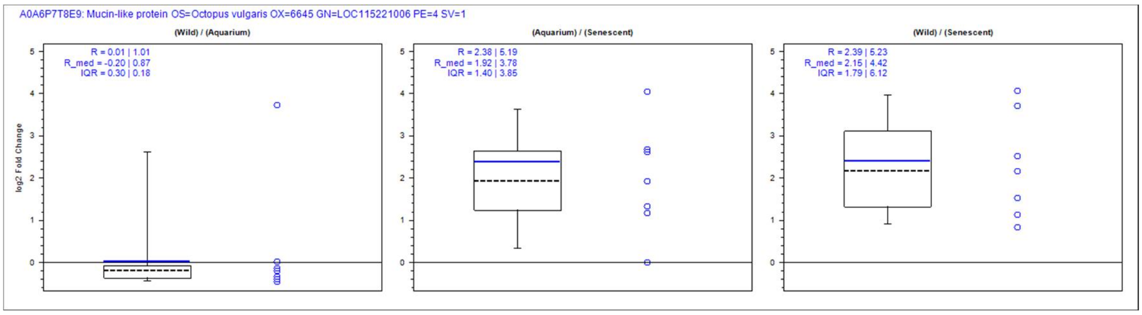

Neural cell adhesion molecule 2 isoform x13

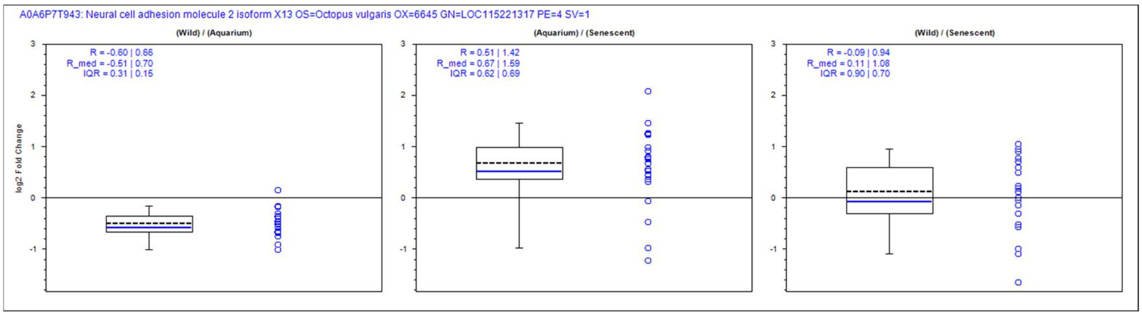

Neuroglian isoform X2

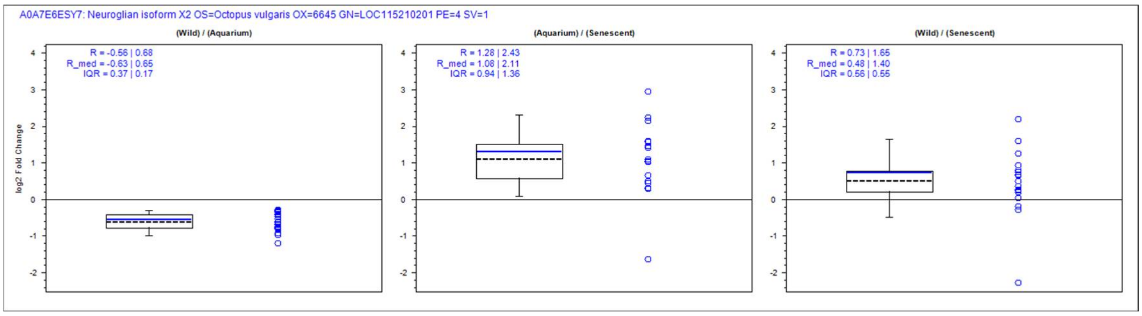

Protocadherin Fat 4

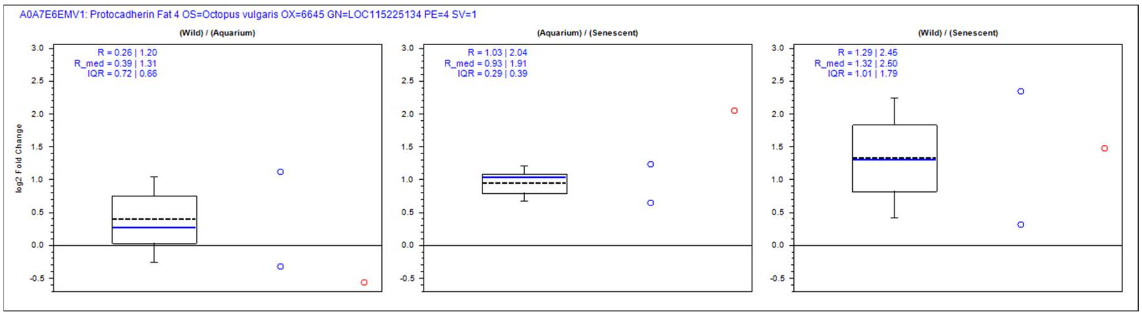

SCO-sponding

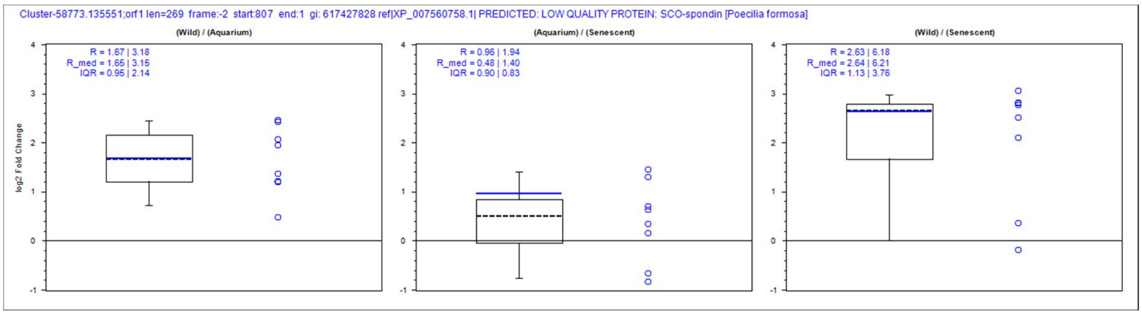

Soma ferritin

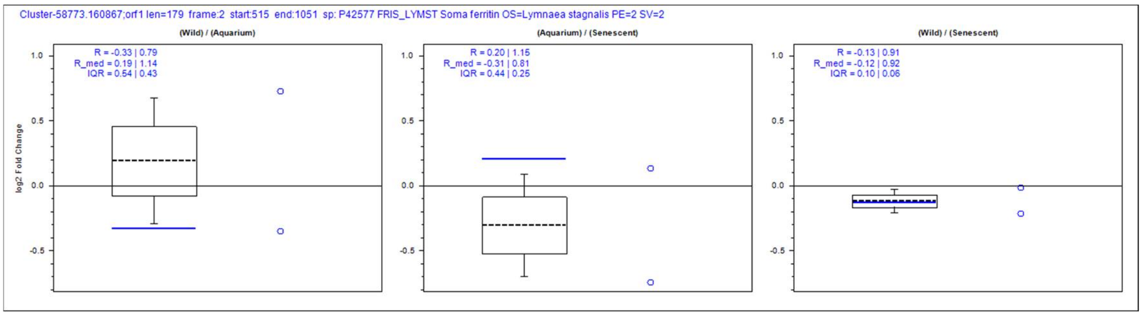

Tetraspanin

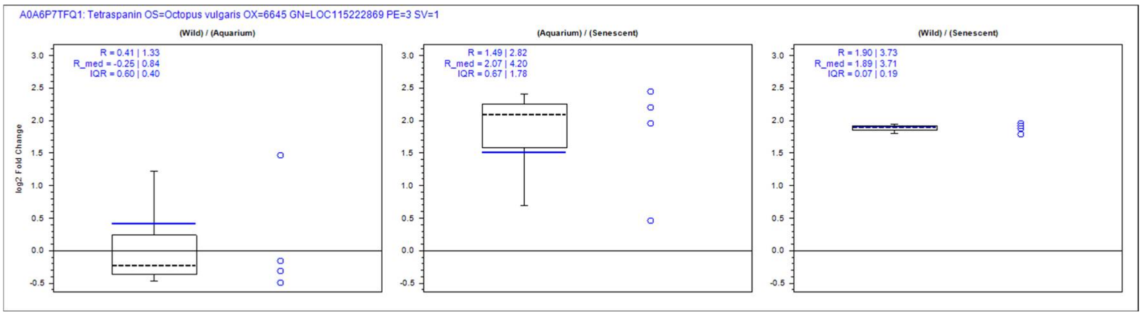

Thioredoxin

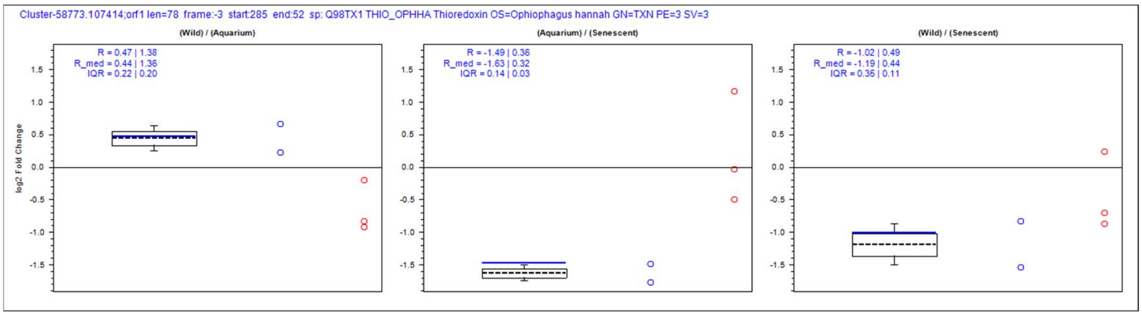

Titin isoform X13

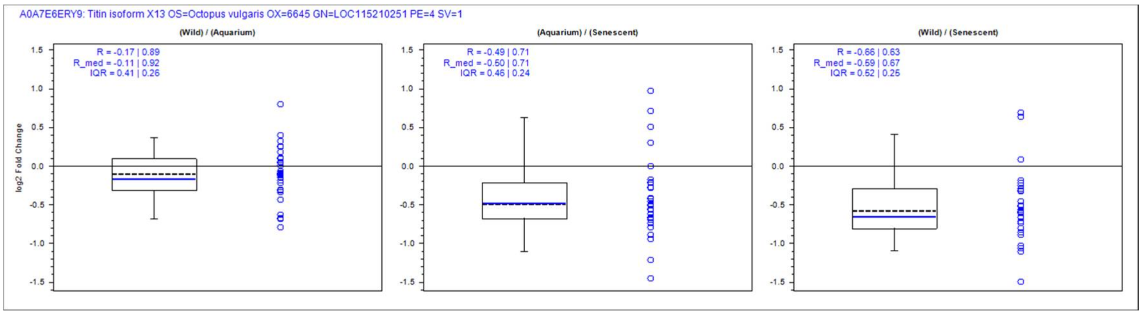

Transcription factor iws-1 isoform X1

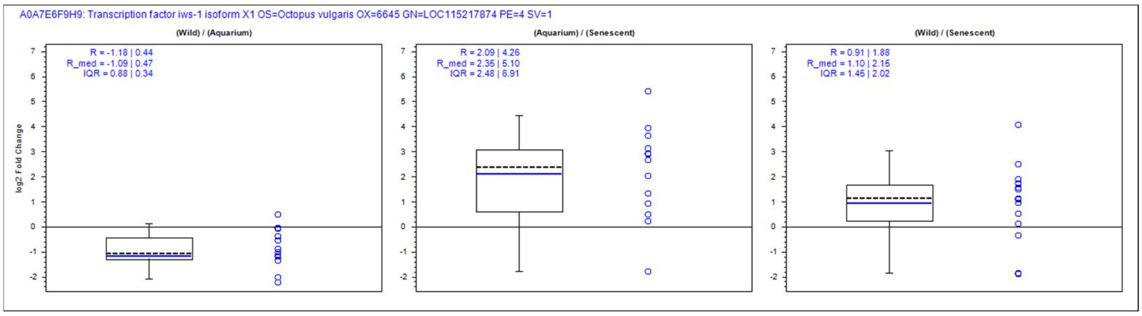

Tubulin aplha chain

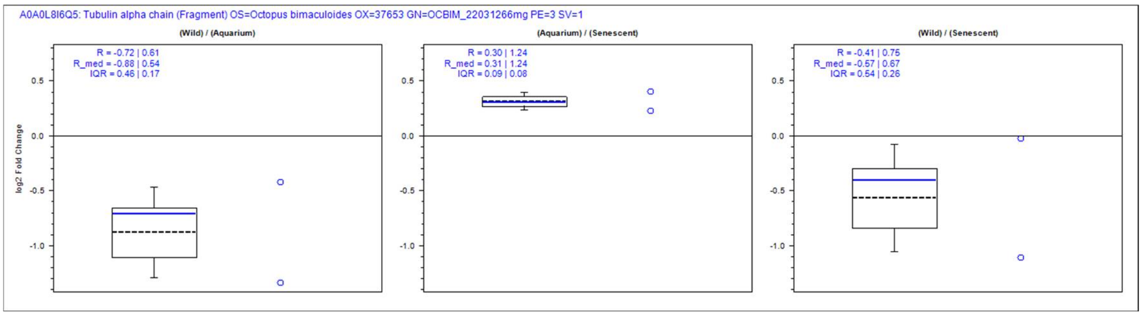

Box plot analysis of the proteins selected as potential biomarkers of health in the time-course study by specimens.

VWFA domain-containing protein

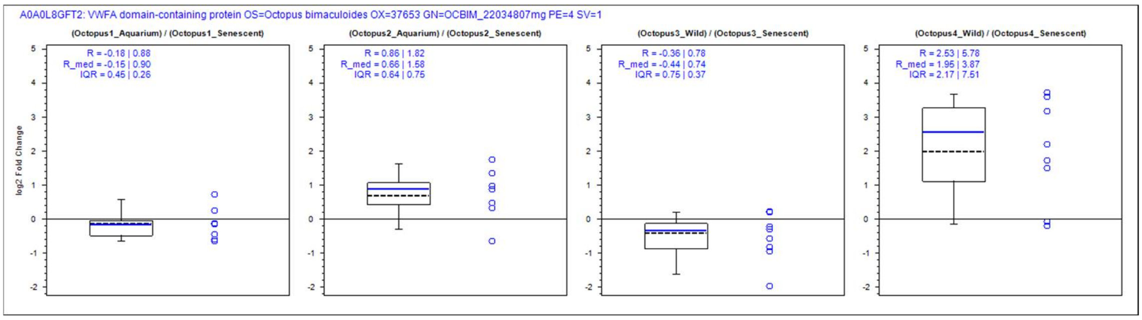

V-type proton ATPase subunit E

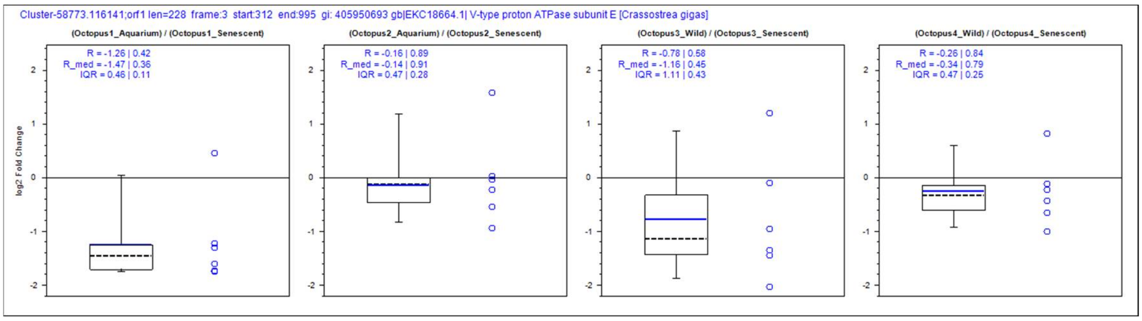

Von Willebrand factor C domain-containing protein 2-like

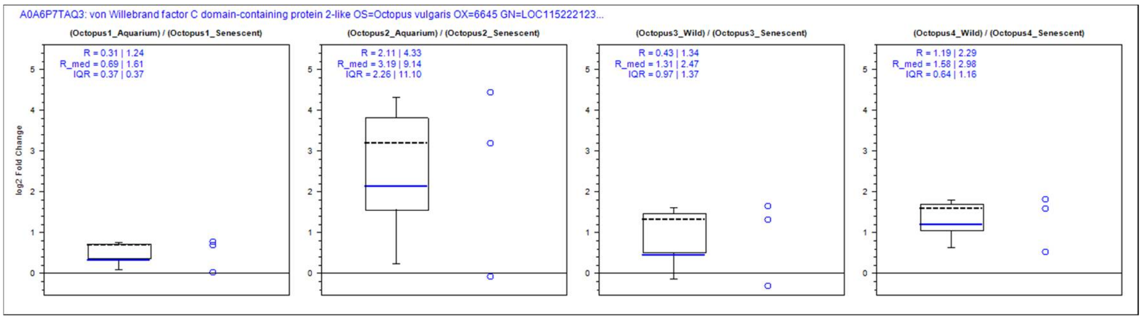

Von Willebrand factor A domain contaning protein 5A-like

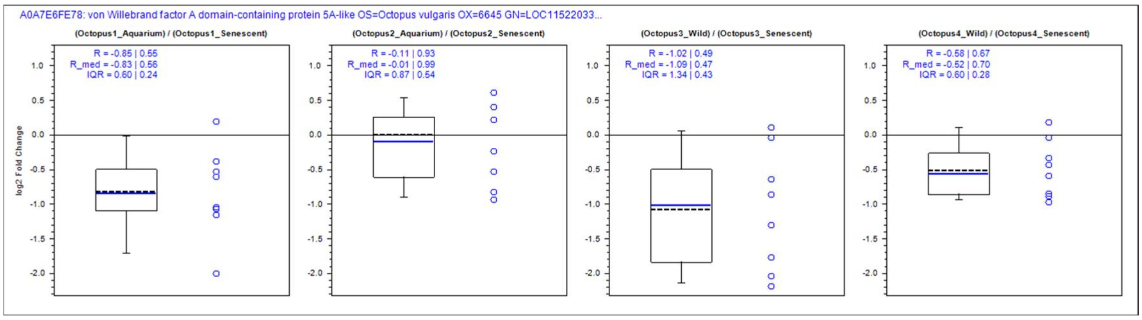

Universal stress protein in QAH/OAS sulfhydrate 3 region isoform X1

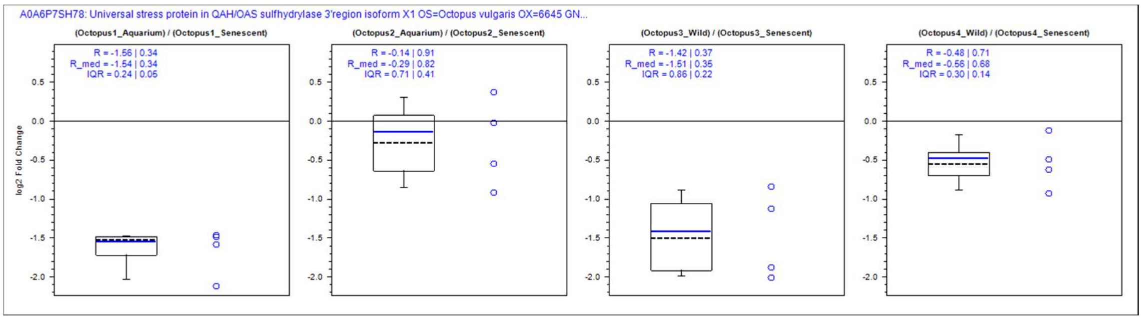

Tumor protein D52 isoform X13

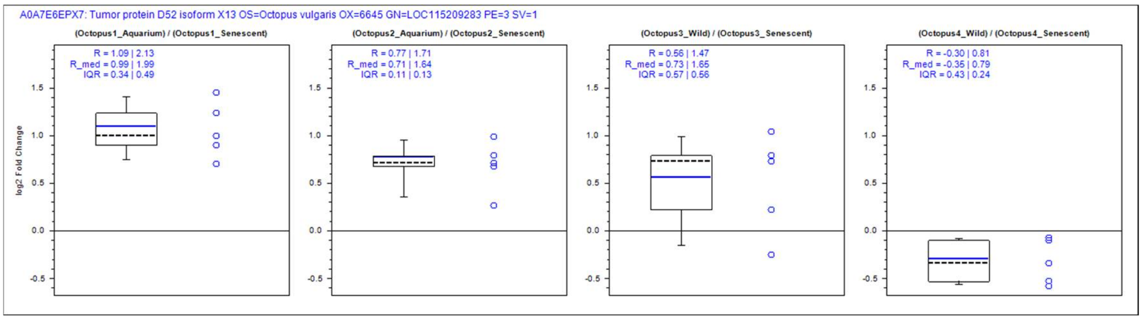

Tropomyosin Tod p.102

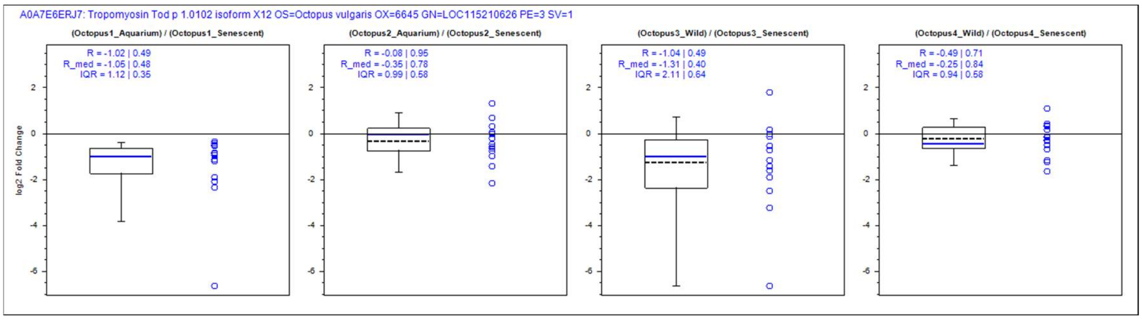

Transgelin

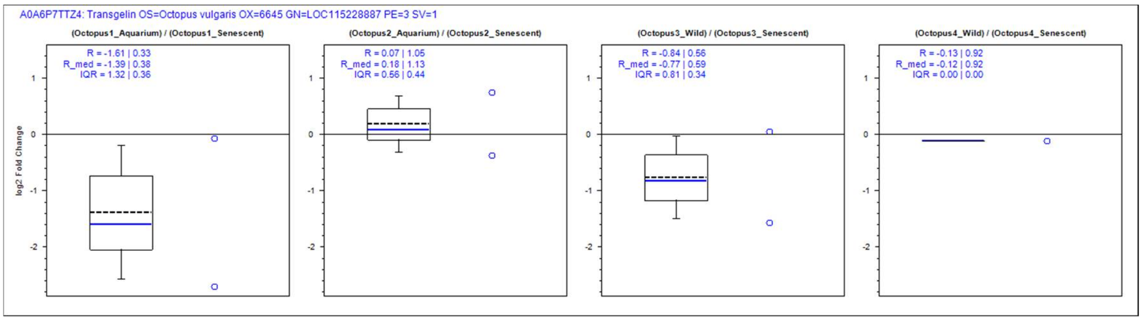

Transcription factor iws-1 isoform X1

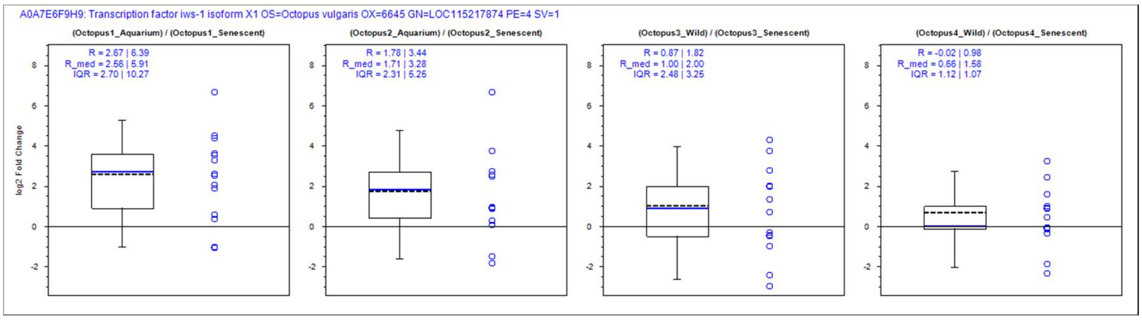

Titin

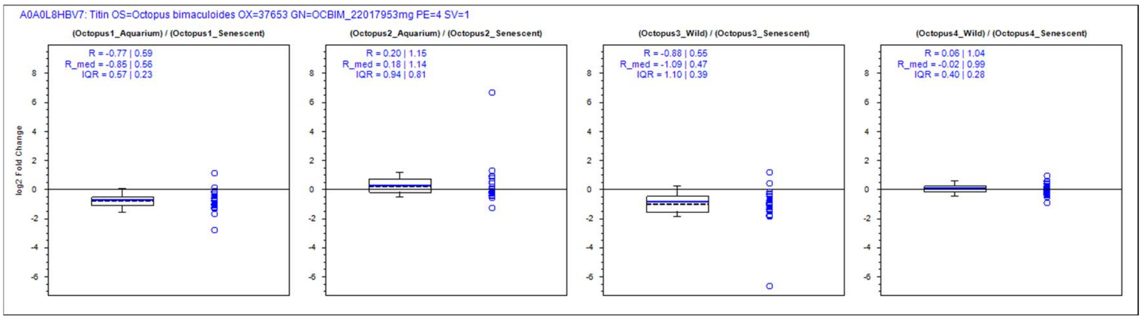

Titin isoform X13

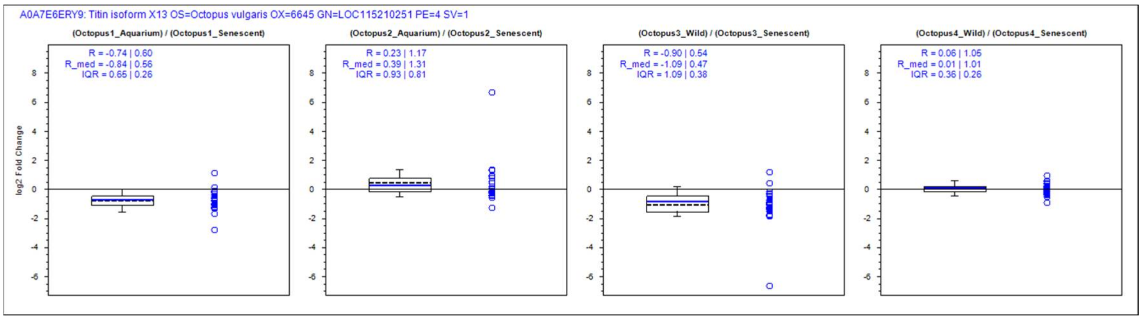

Thioredoxin-disulfite reductase

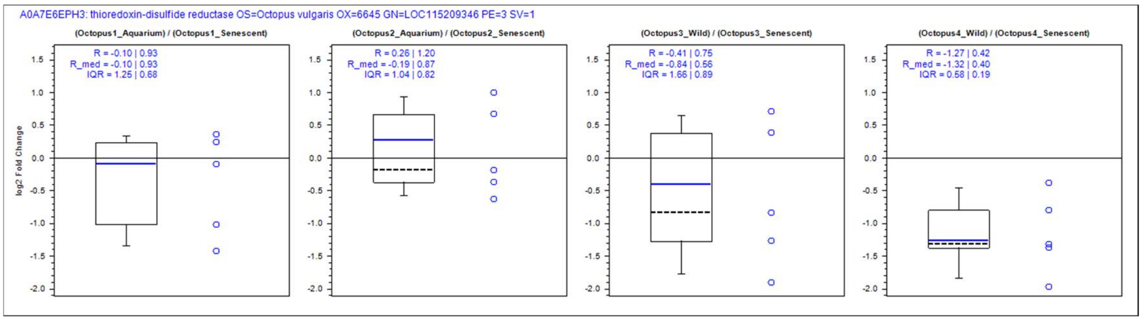

Tetraspanin

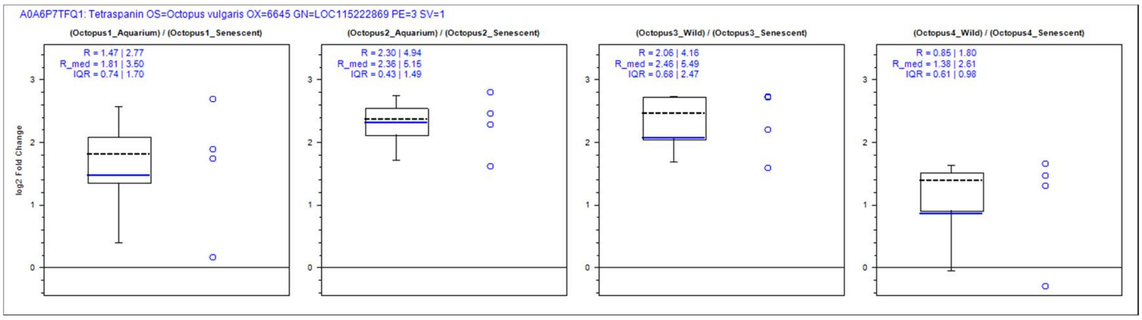

Succinate-CoA ligase [GDP-forming] subunit beta, mitochondrial

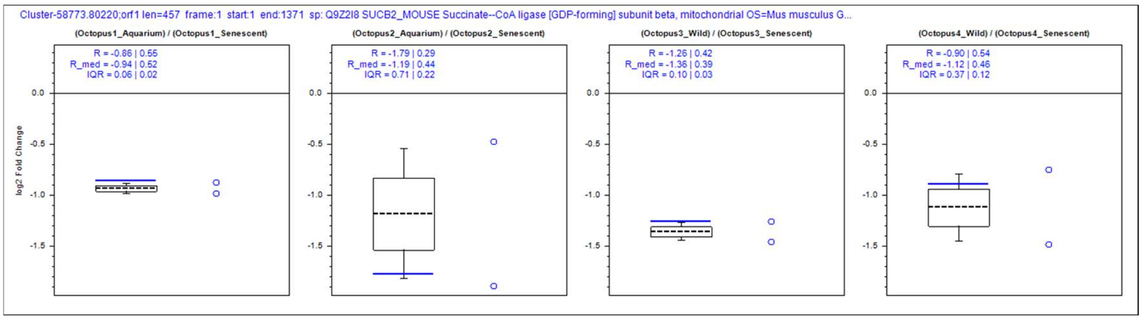

SH3 domain-containing protein (Fragment)

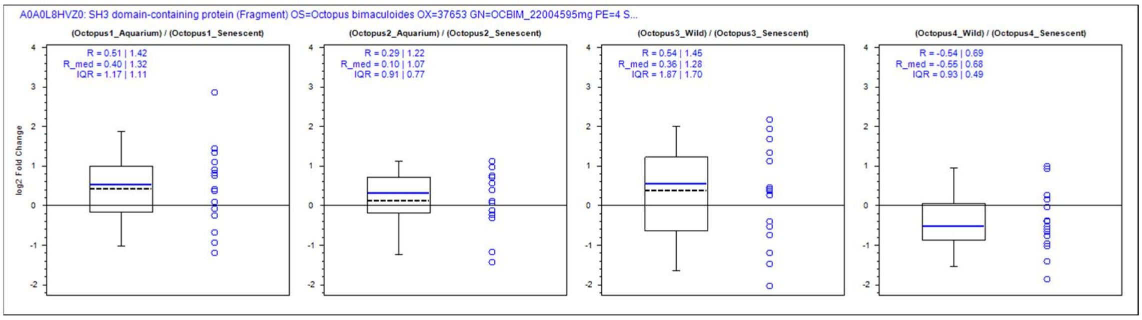

SCO-spondin

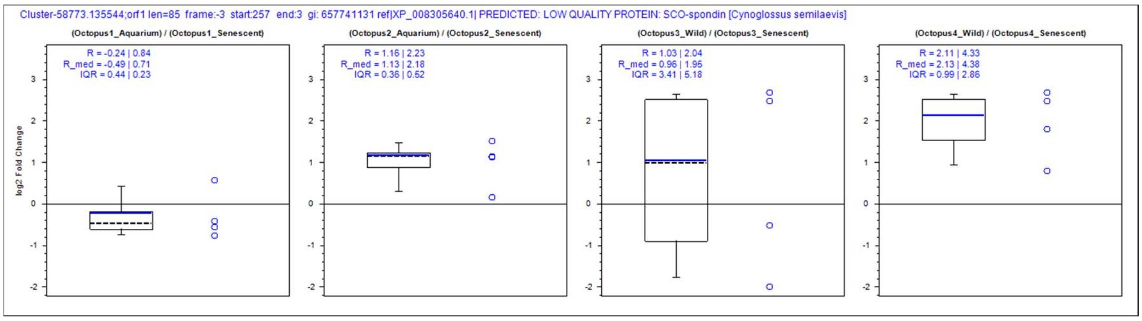

RING-type E3 ubiquitin transferase

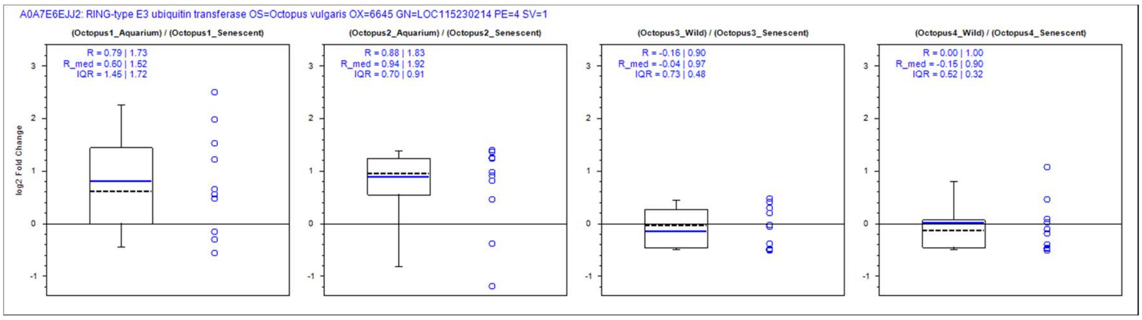

Rho GDP dissociation inhibitor

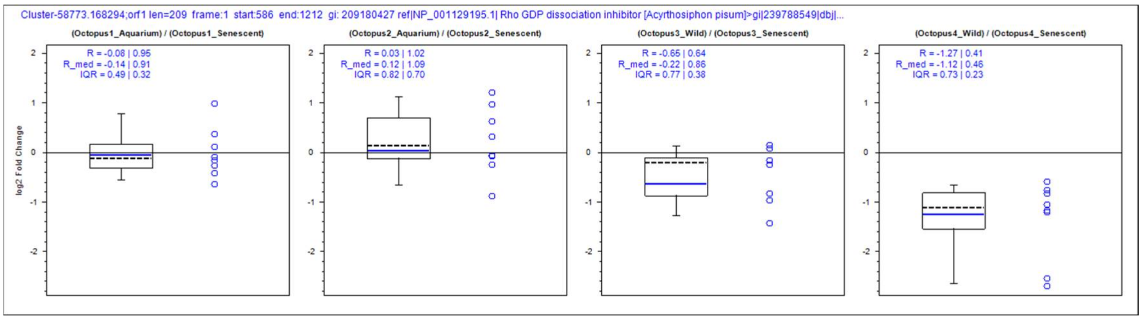

Ras-related protein Rab-2

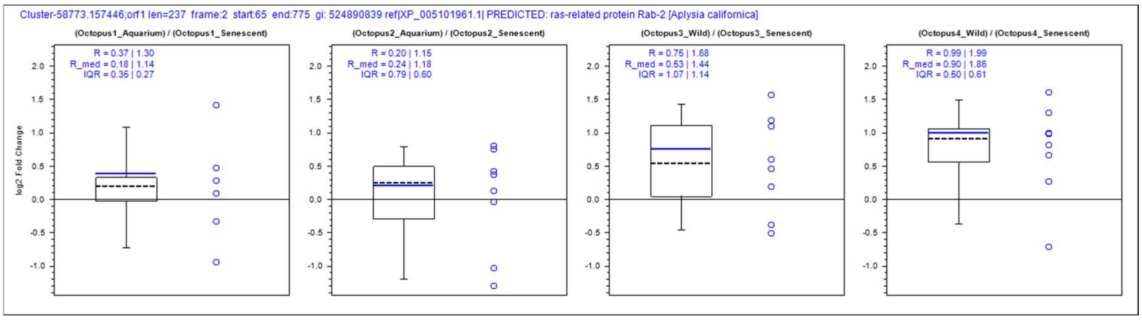

Protocadherin Fat 4

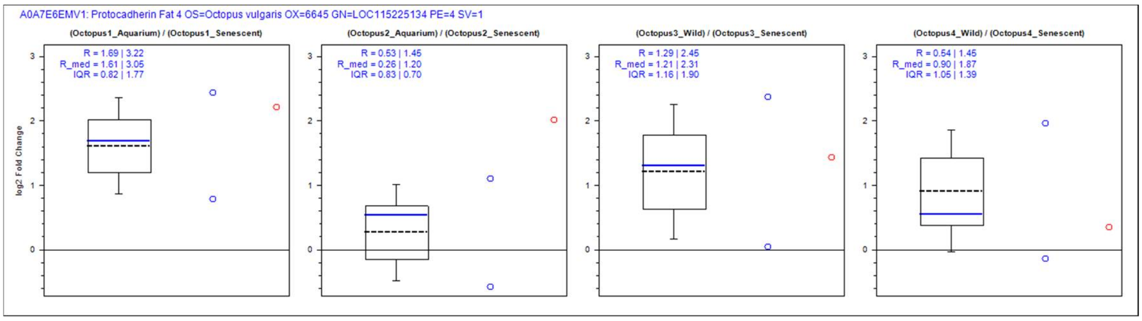

Omega-crystallin

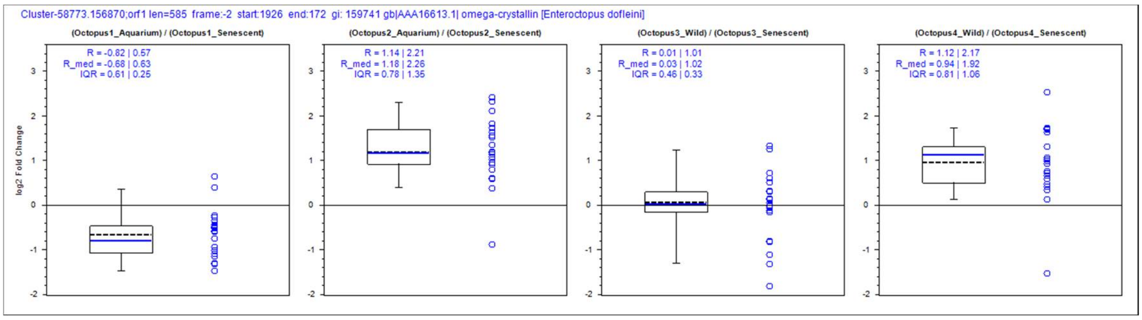

Neuroglian isoform X1

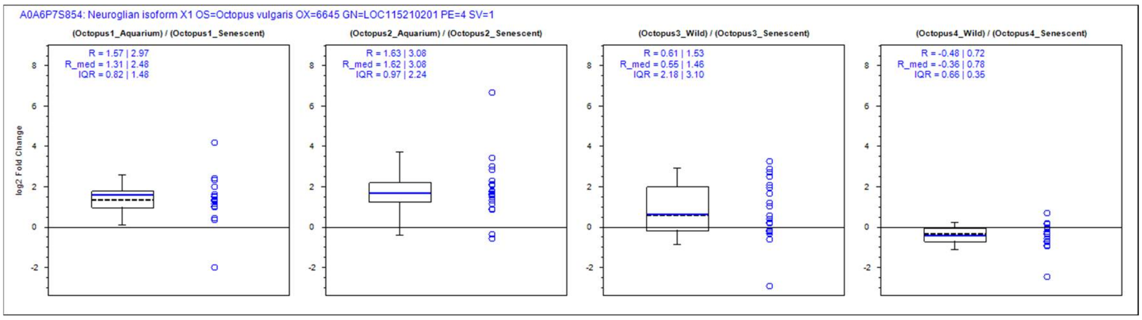

Neural cell adhesion molecule 2 isoform X13

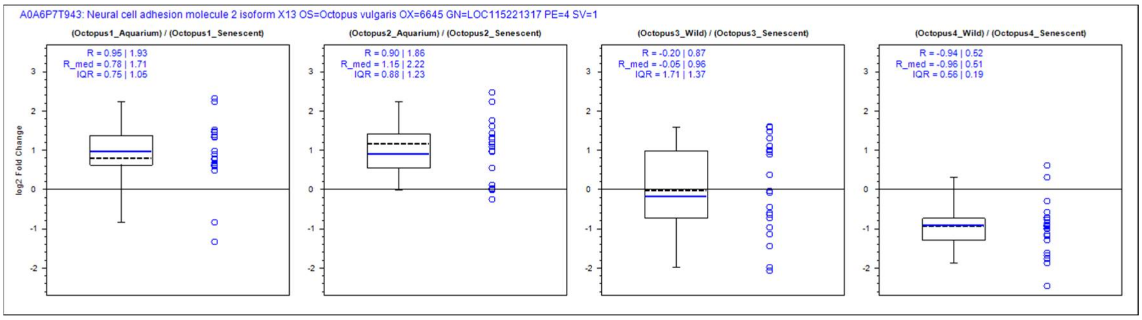

Mucin-4-like

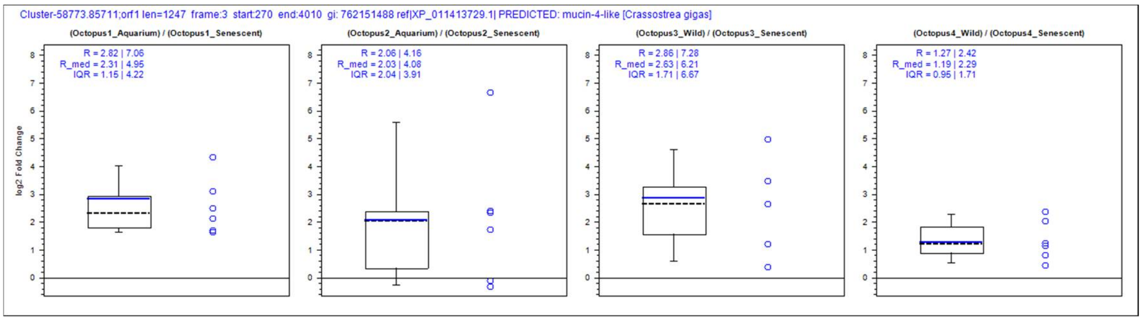

Mucin-19-like

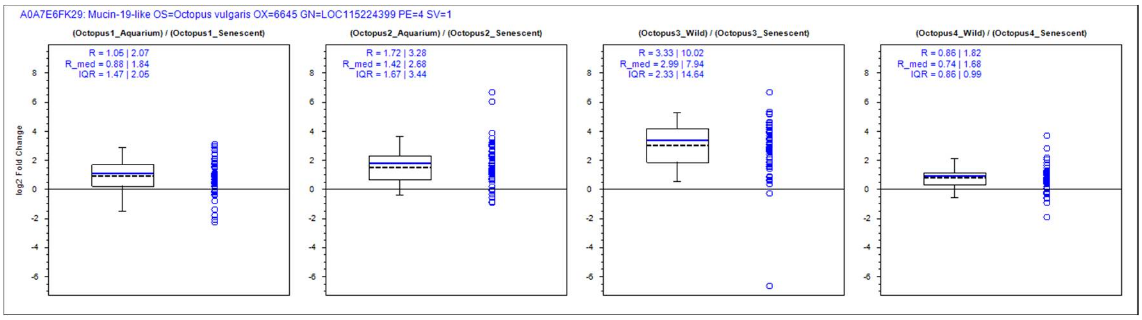

MAM and LDL-receptor class A domain-containing protein 2-like

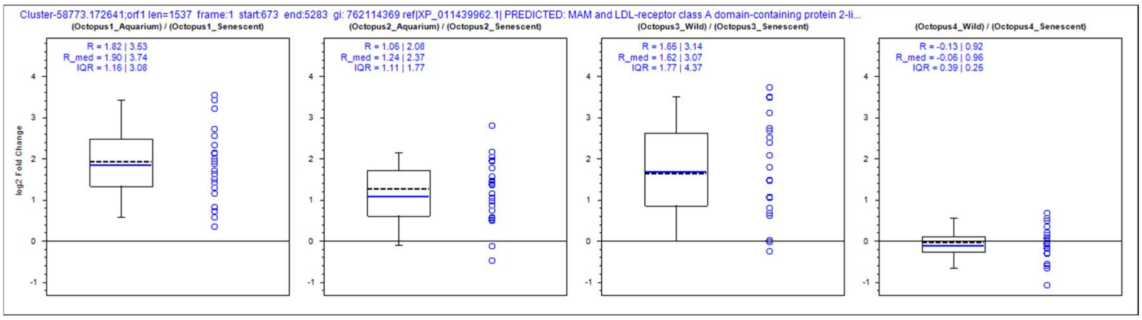

LLGL scribble cell polarity complex component 2 isoform X2

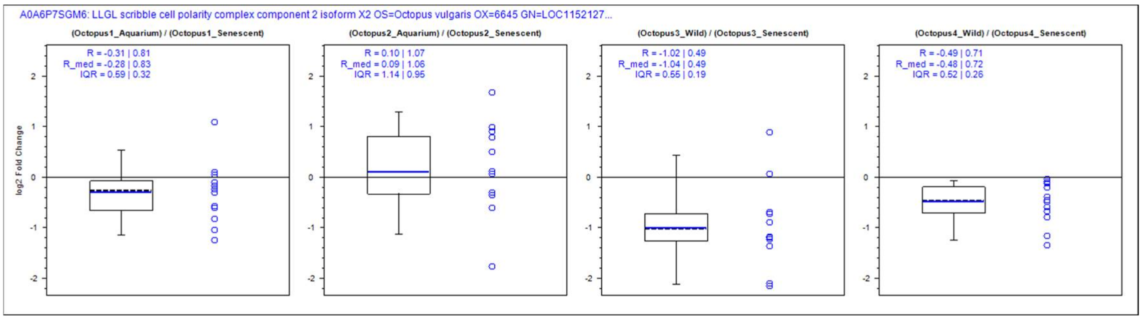

Kynureline—oxoglutarate transaminase

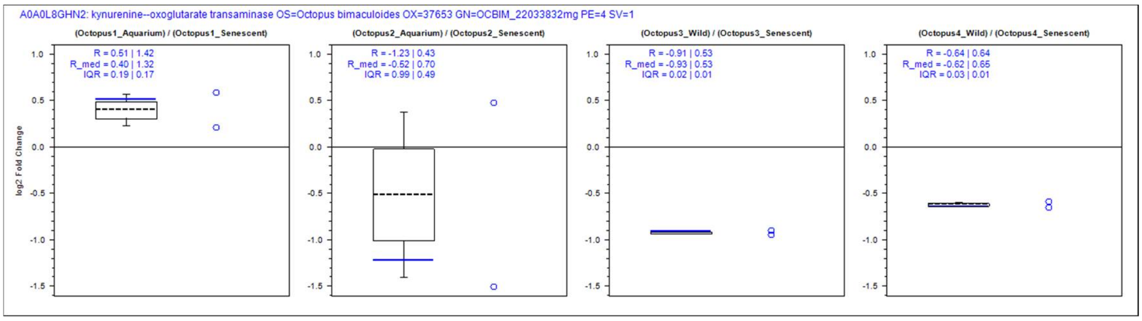

Insulin-like growth factor-binding protein complex acid labile subunit isoform X1

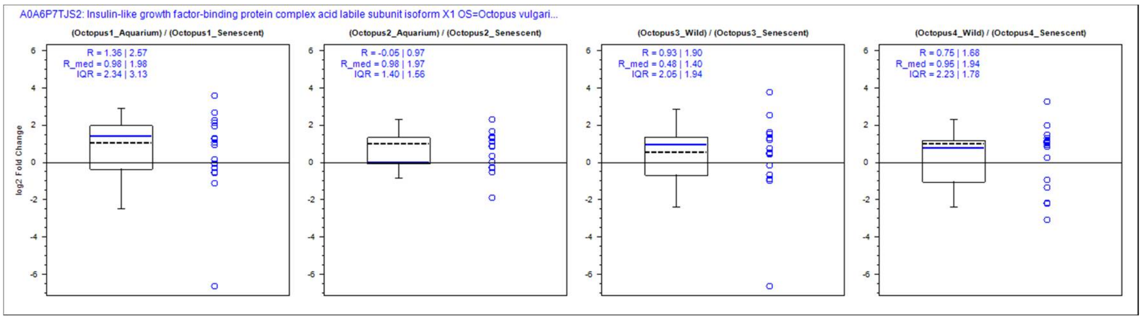

Histone H3 v1 isoform X2

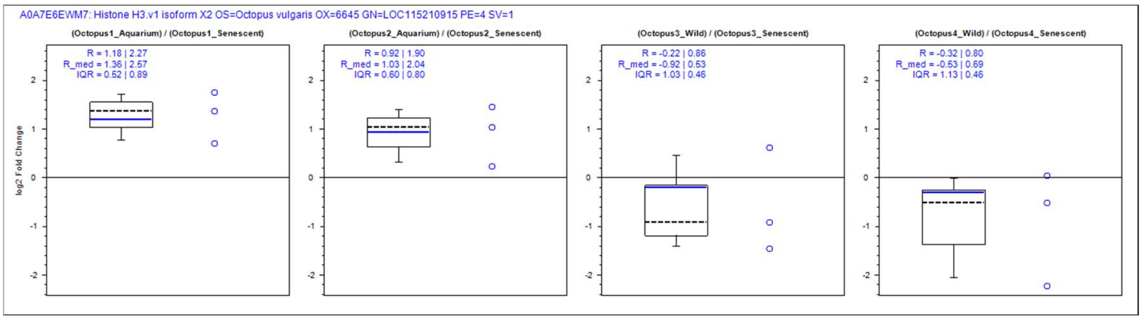

Histone H1-like

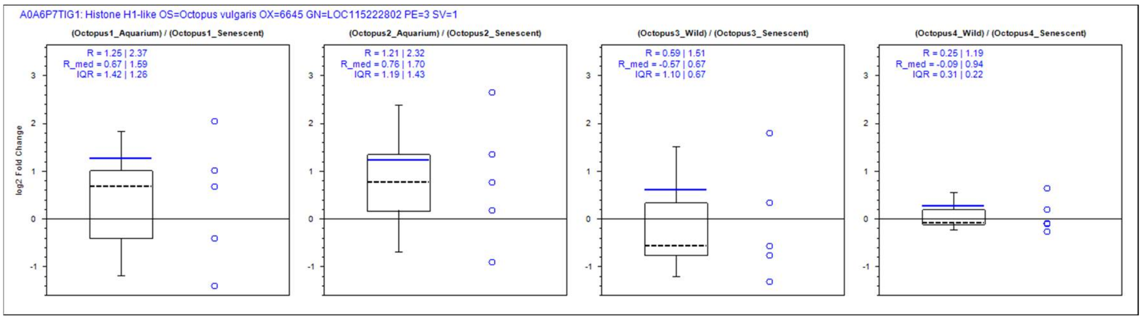

Hemocyanin A-type

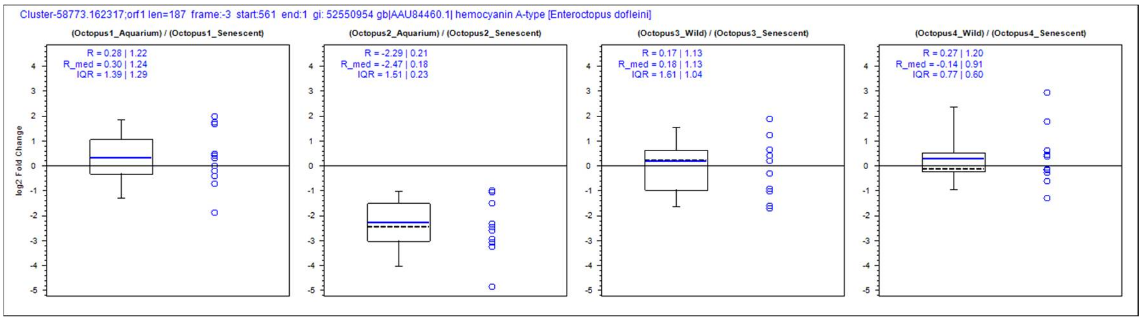

Hemocyanin 1-like

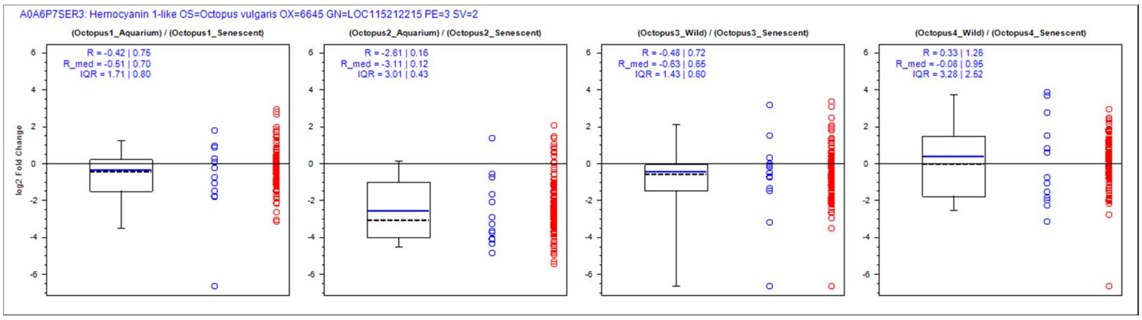

Heat shock protein 83 isoform X1

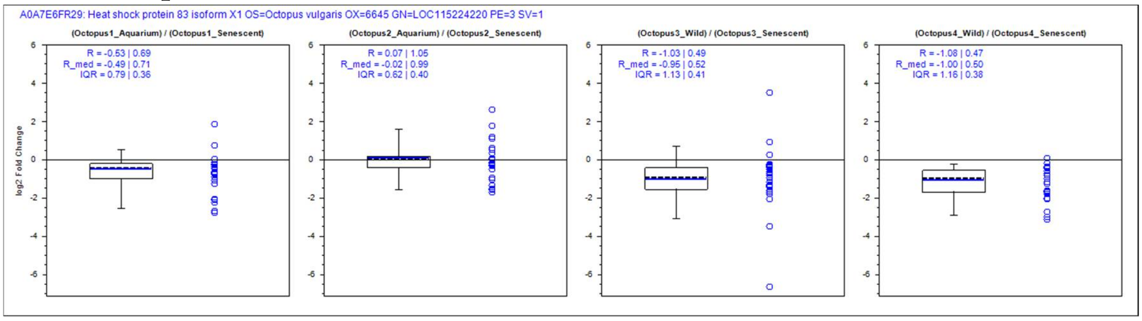

H+ transporting two sector ATPase

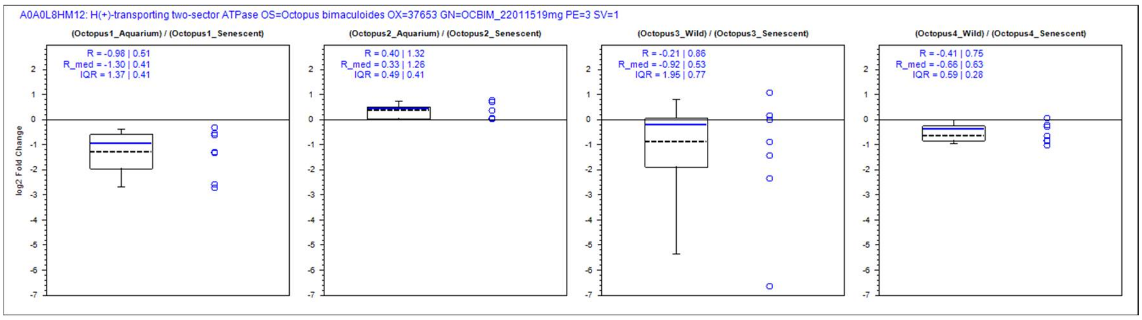

Golgi-associated plant pathogenesis-related protein 1-like

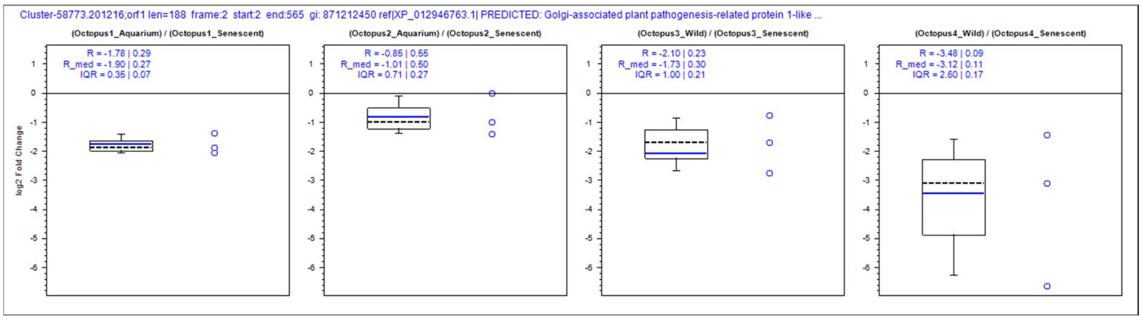

Glutamine synthetase

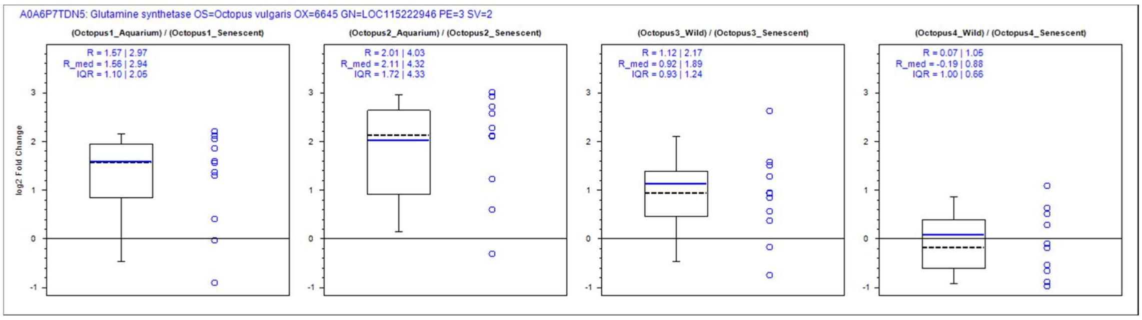

Glutamate dehydrogenase NAD(p)(+)

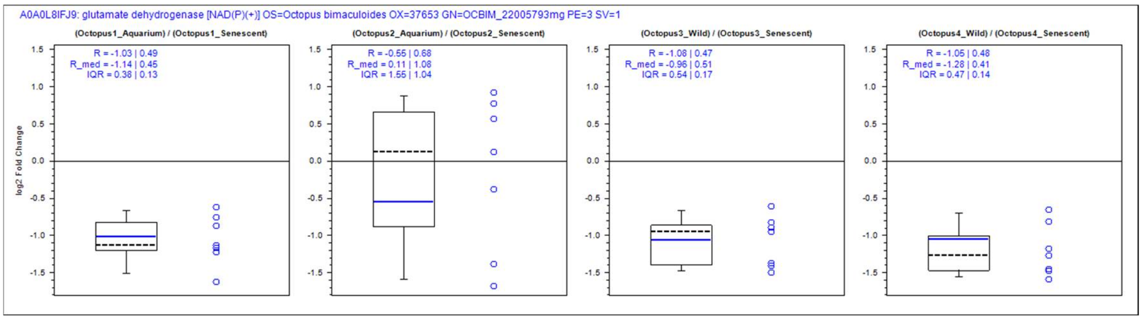

Gelsolin-like protein

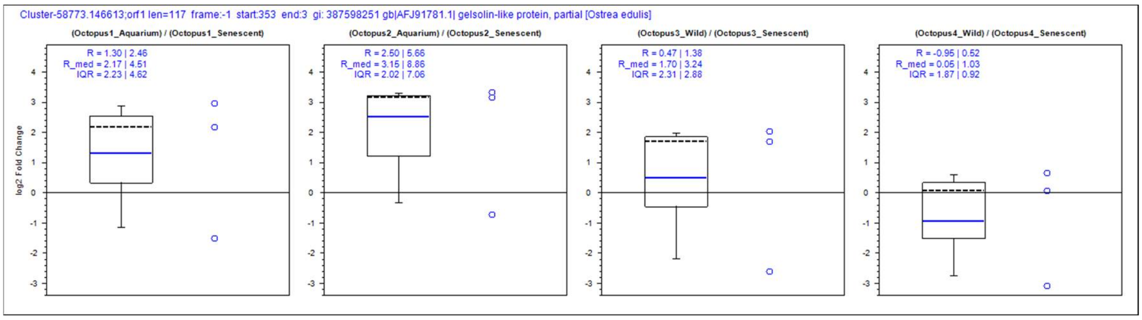

Fascin

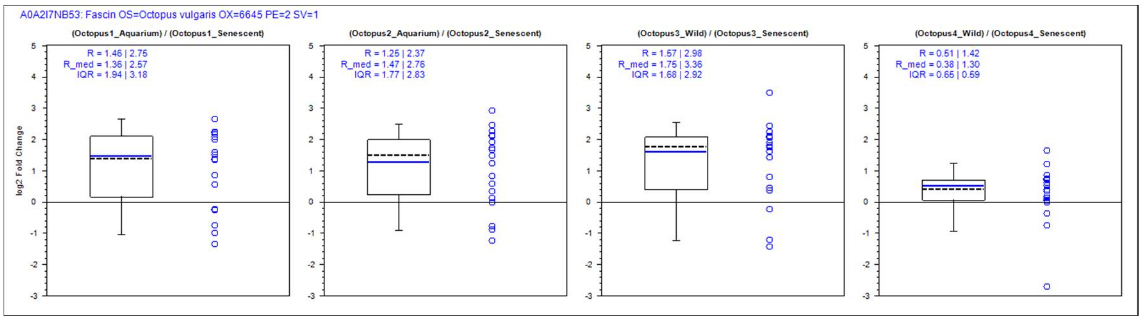

EF-hand domain-containing protein

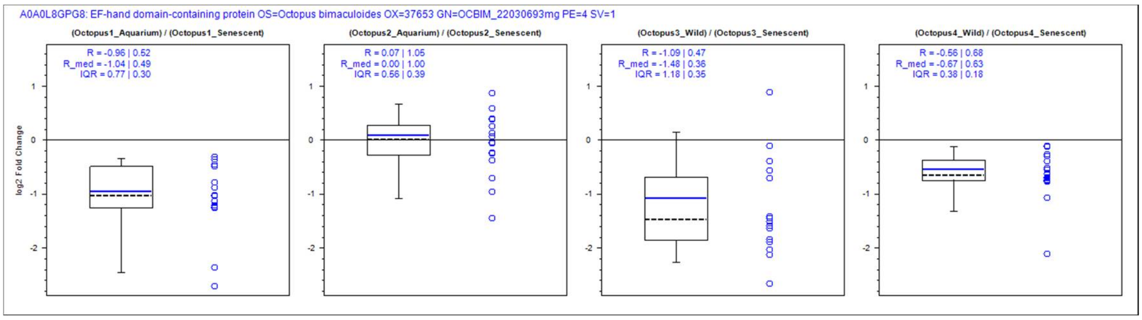

Deoxynucleoside triphosphate SAMHD-1-like isoform X2

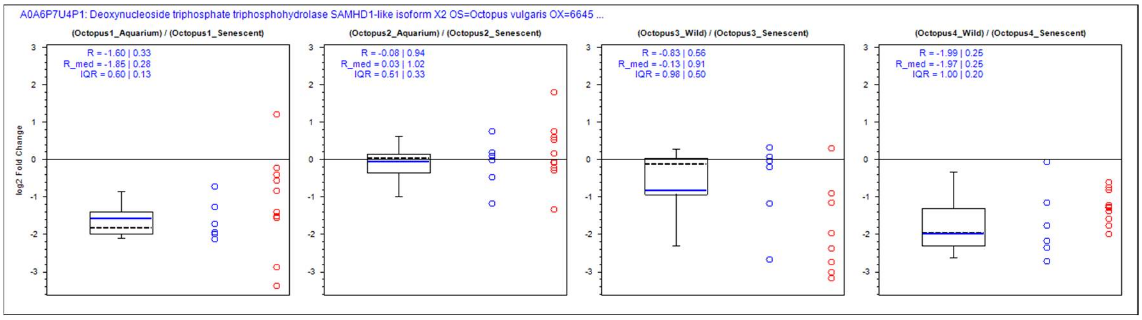

Deleted in malignant tumor 1 protein-like

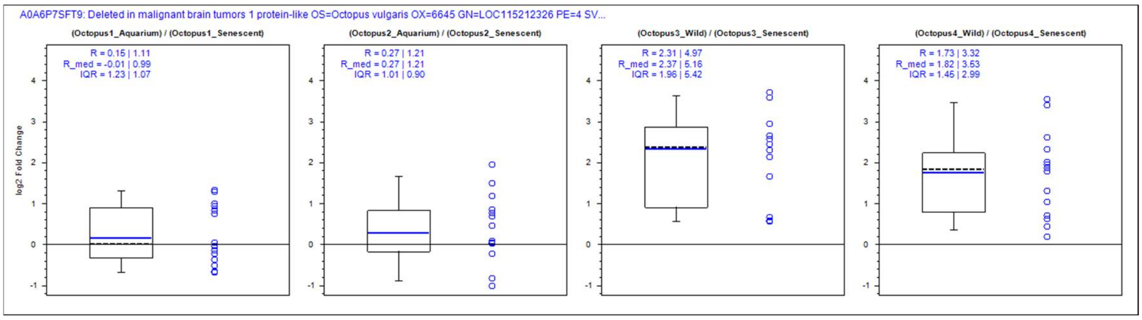

Cytochrome c oxidase copper chaperone

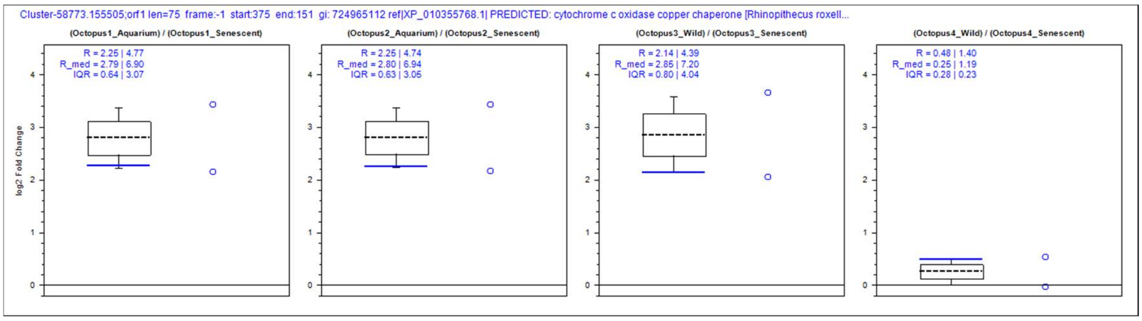

CuZn superoxide dismutase

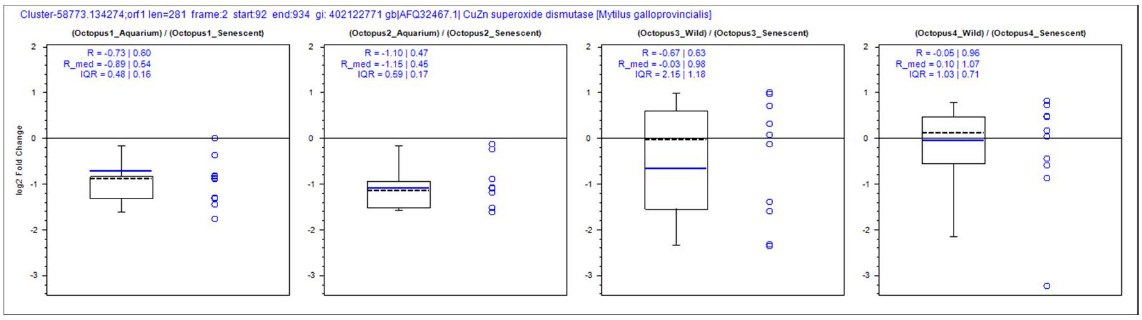

Coronin

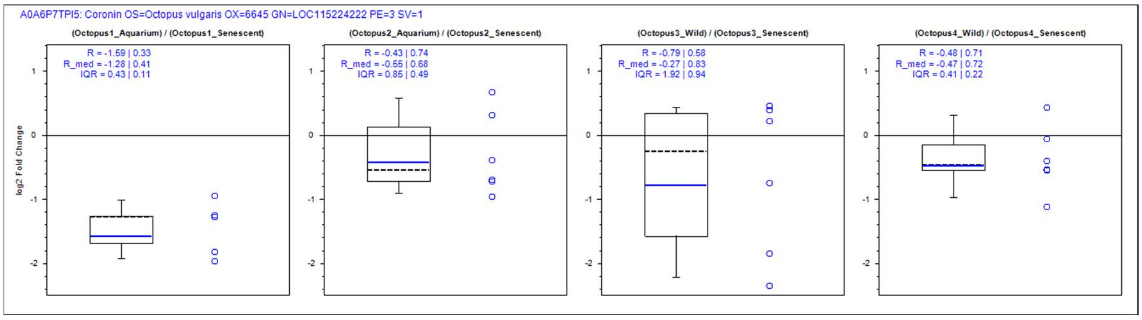

Collagen alpha-4(VI) chain-like isoform X1

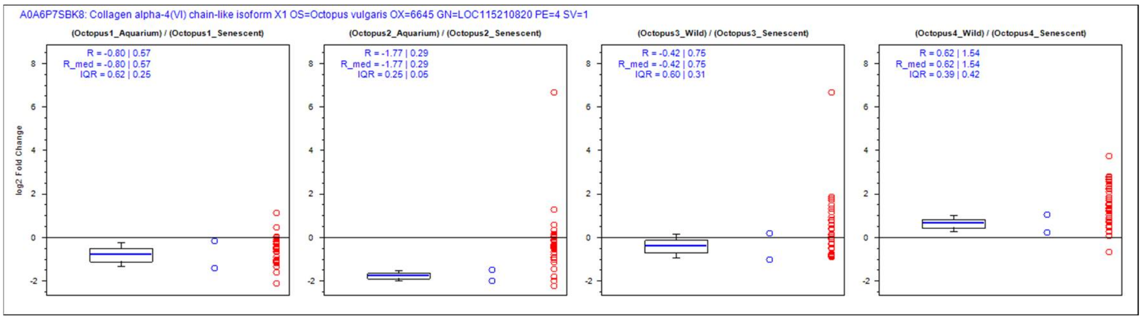

Collagen alpha-3(VI) chain

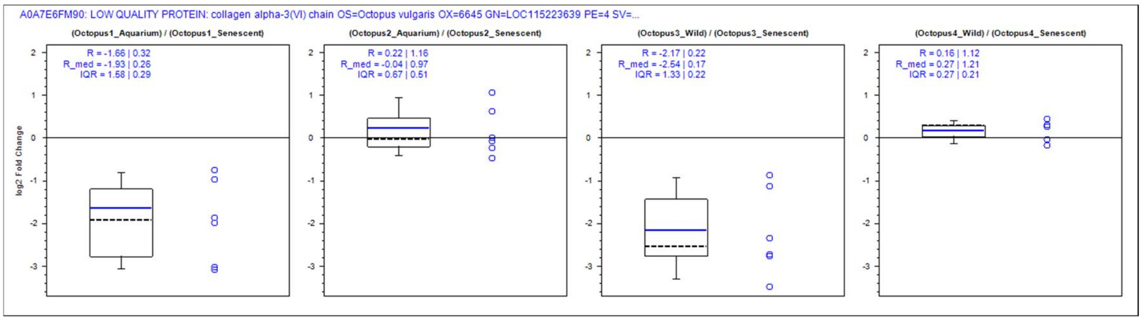

Collagen alpha-4(VI) chain

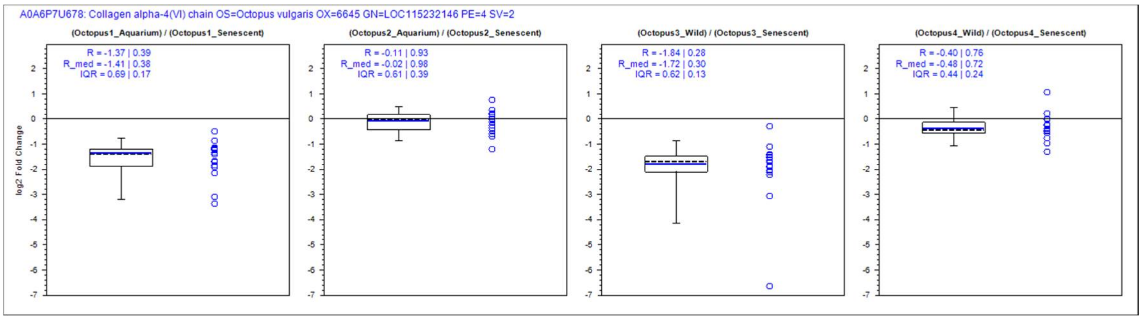

Cofilin

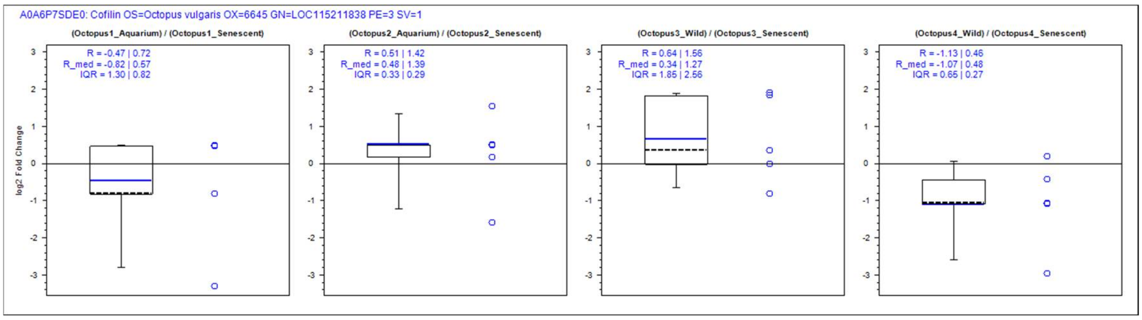

Co-chaperone protein daf-41

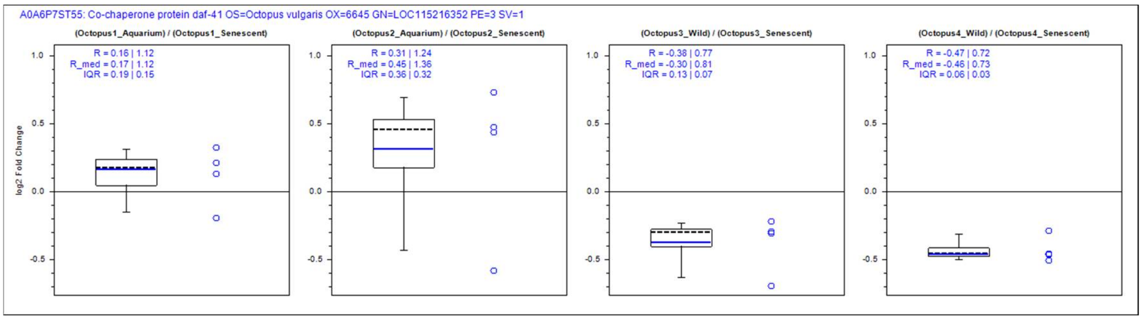

CD109 antigen isoform X1

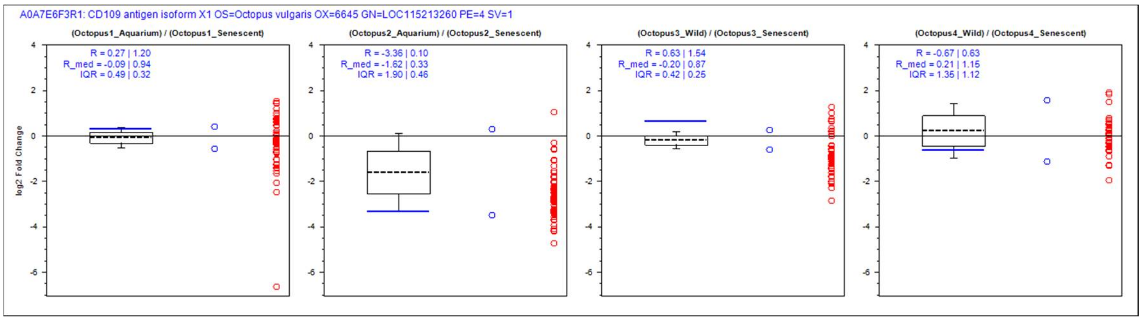

CCHC-type zinc finger nucleic acid binding protein

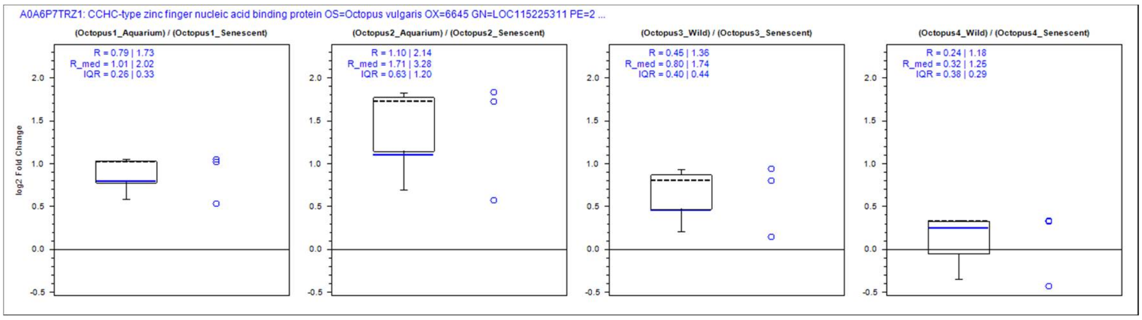

Caspase-7-like

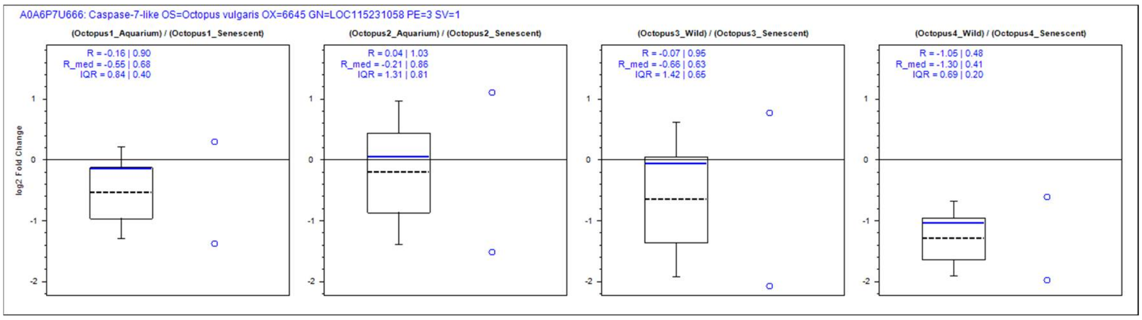

Cartilage matrix protein

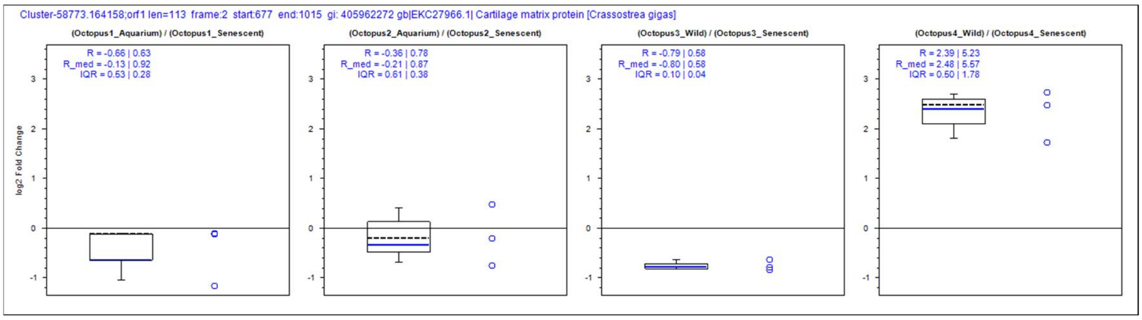

Calpain-9-like

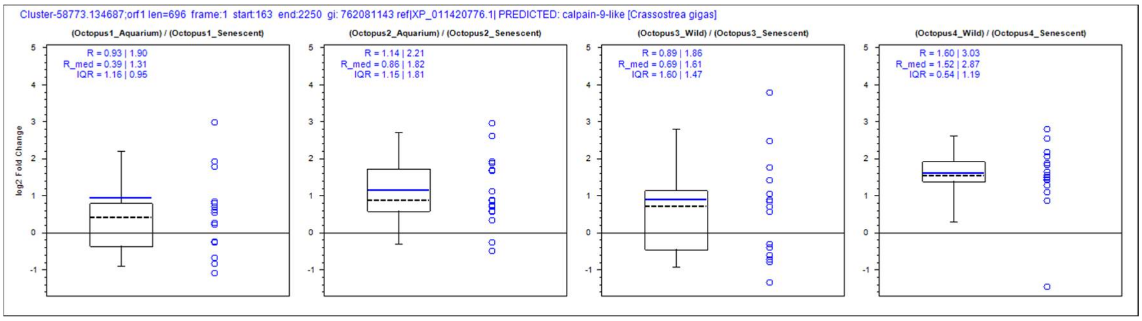

Arginosuccinate lyase

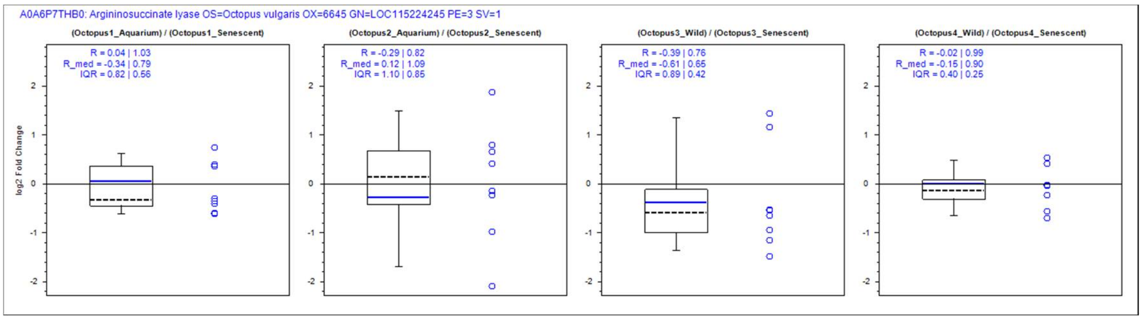

Annexin

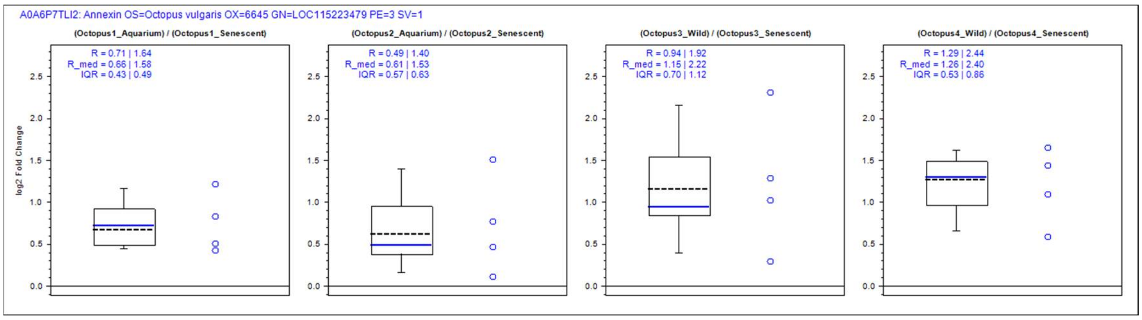

Aminopeptidase

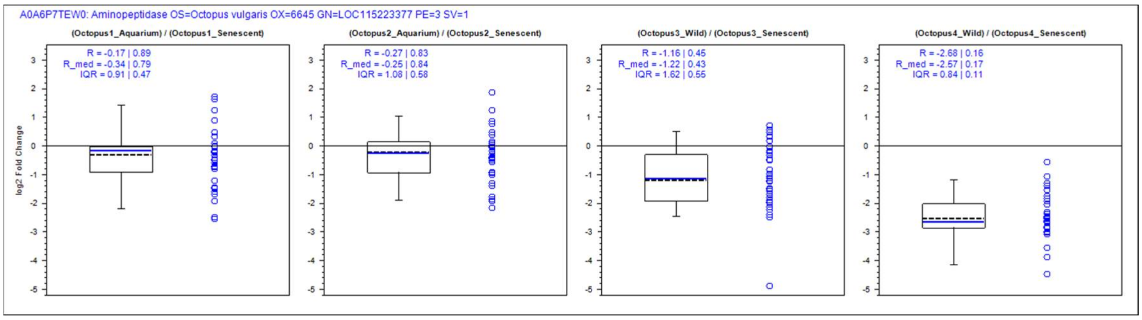

Adhesion G-protein coupled receptor G6

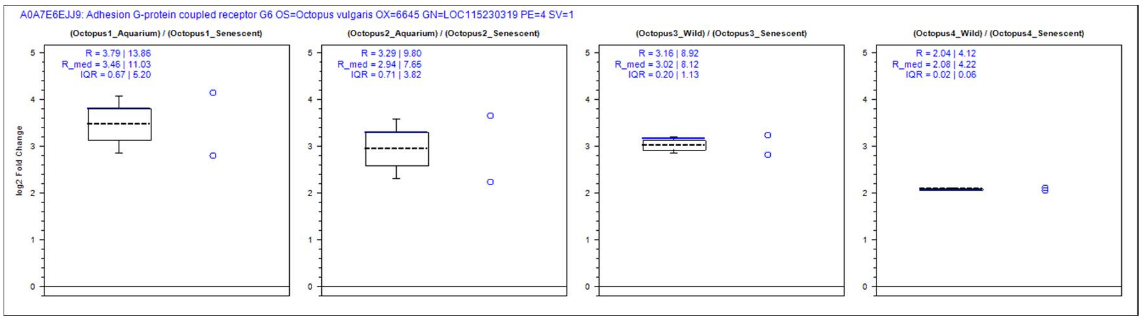

Actin-related protein 2/3 protein complex

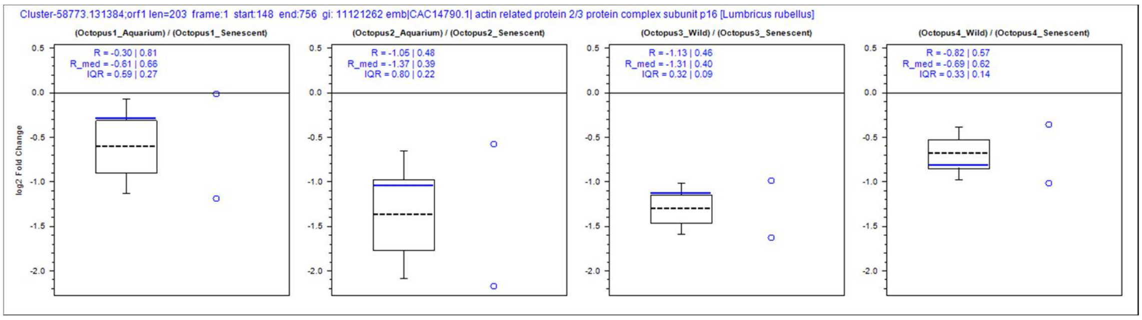

ACTB_G1

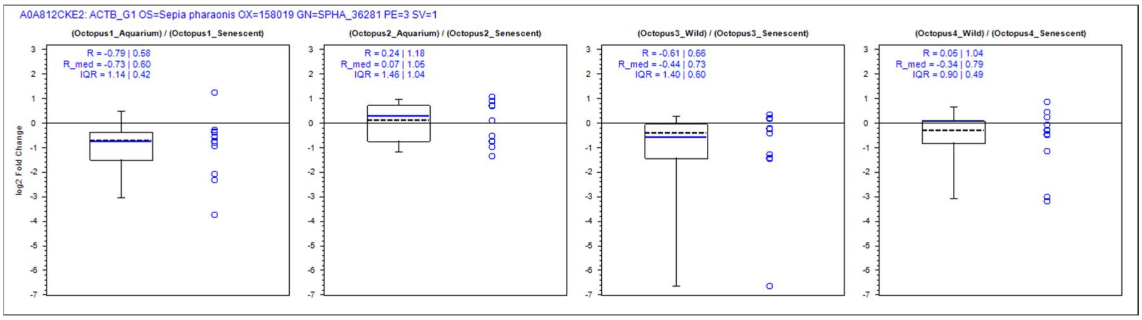

Acid mammalian chitinase

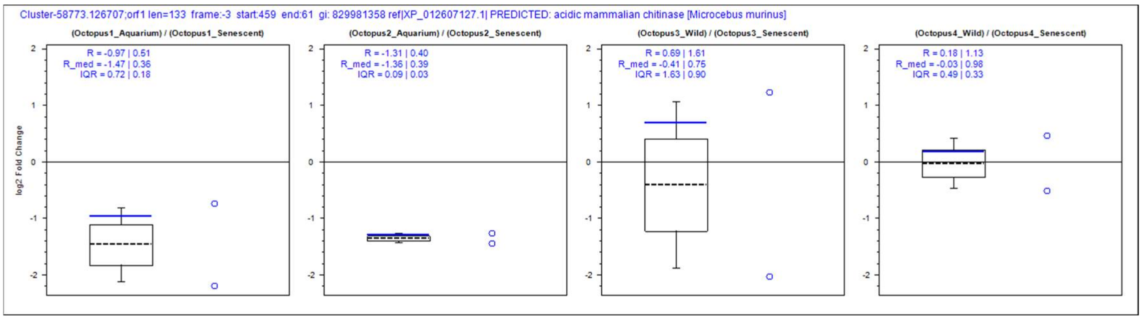

70 kDa neurofilament protein isoform X1

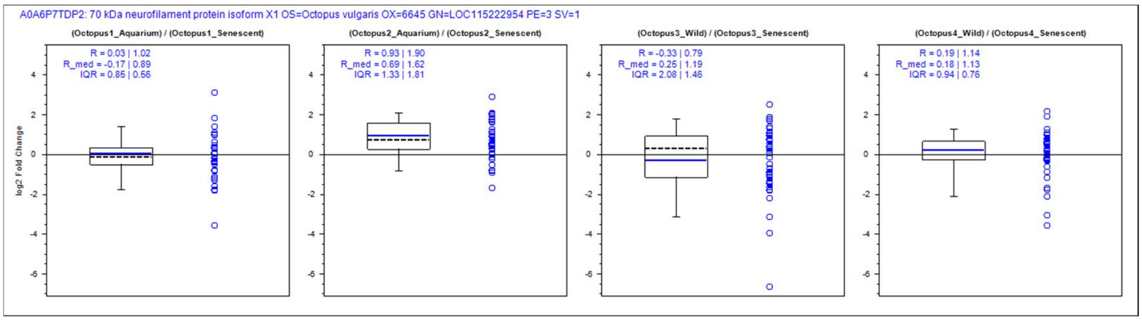

70 kDa neurofilament protein

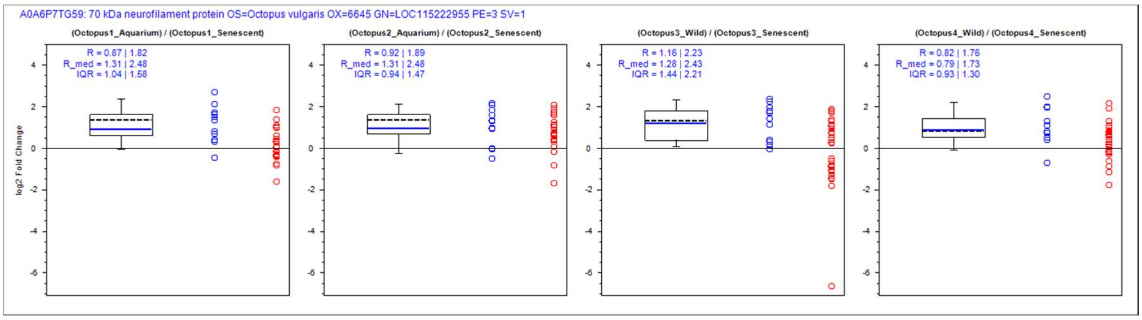

60S ribosomal protein L13

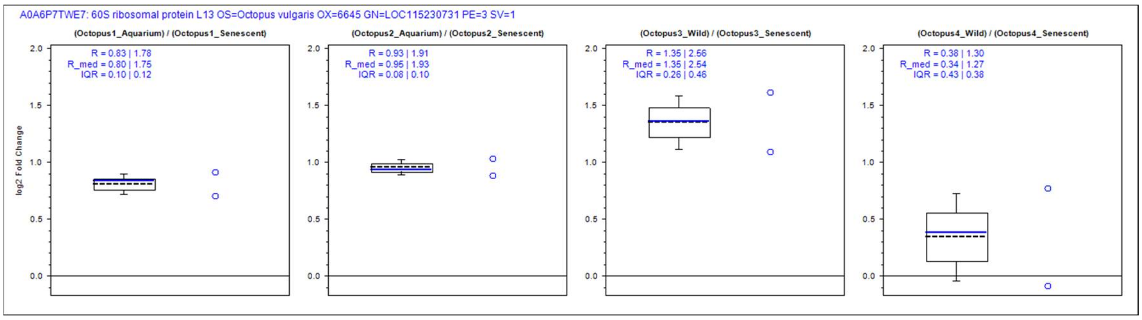

VWFD domain-containing protein

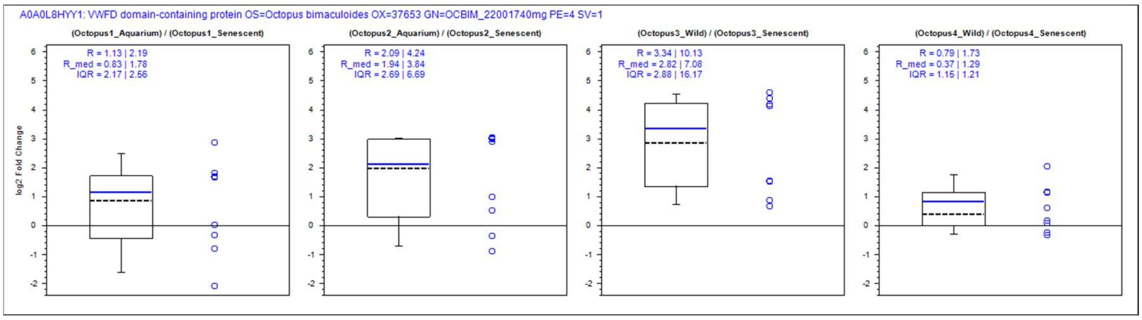

Box plot analysis of the proteins selected as potential biomarkers of sex-specific study of senescent animals.

Thioredoxin-dependent peroxiredoxin

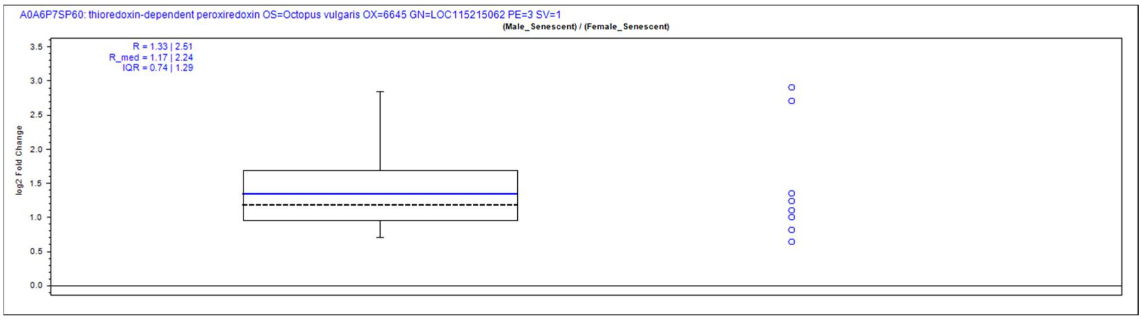

Mucin-19 protein

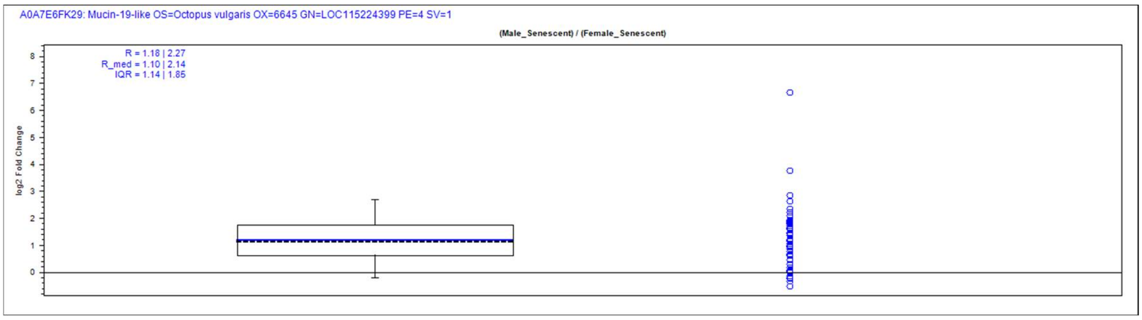

Collagen

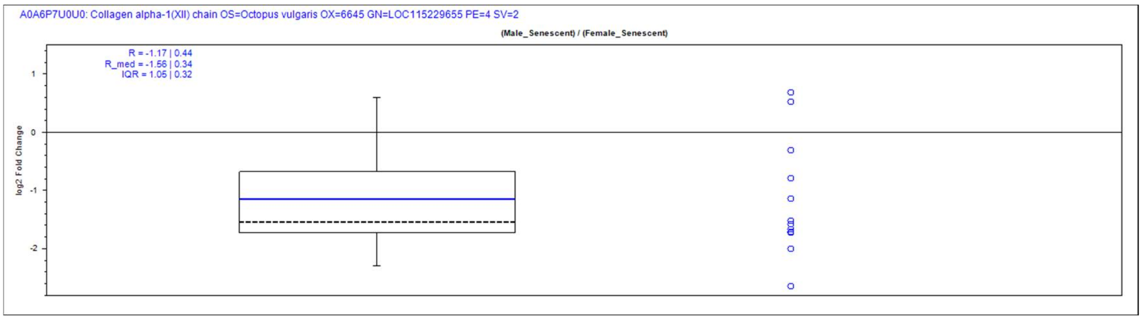

Cofilin

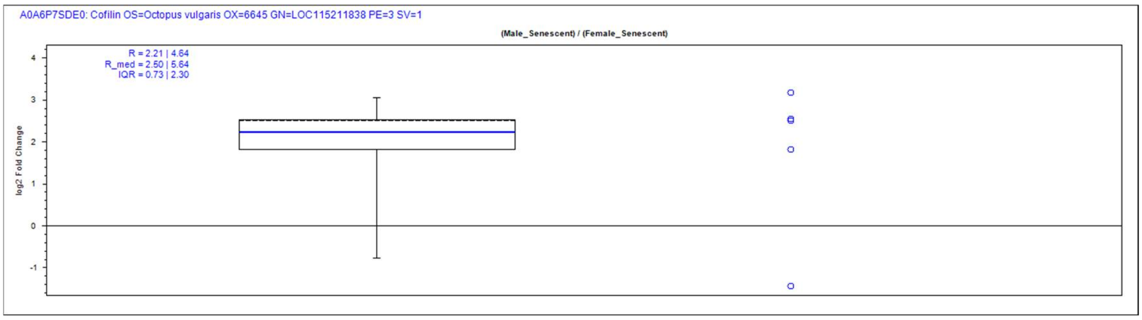

Chitinase

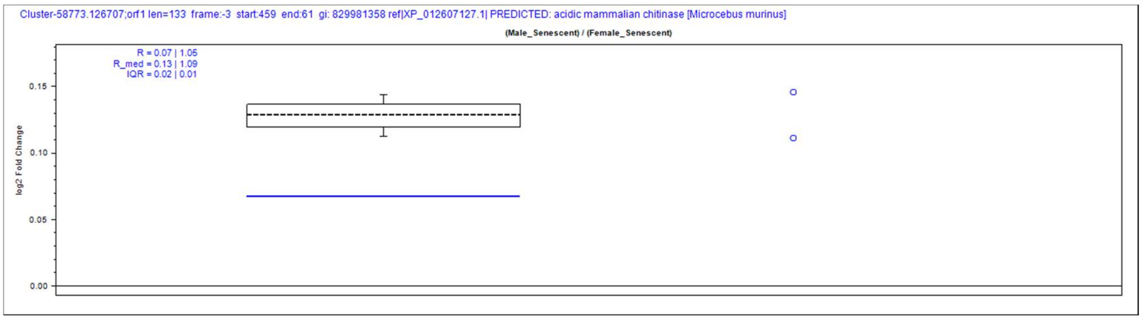

SCO-spondin protein

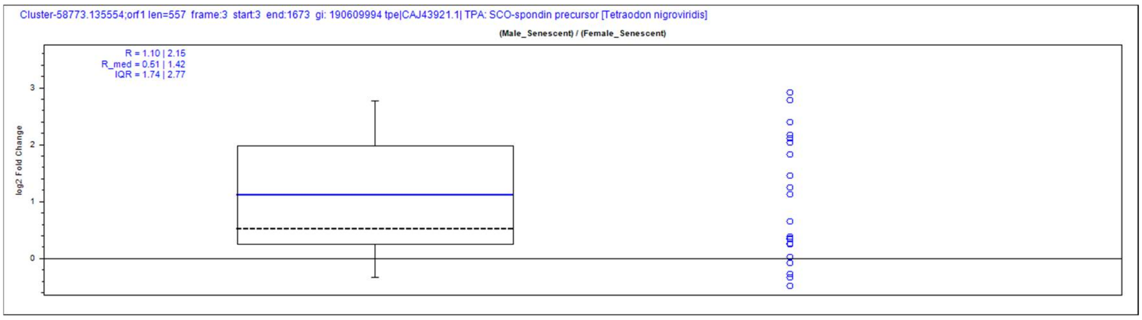

Tetraspanin

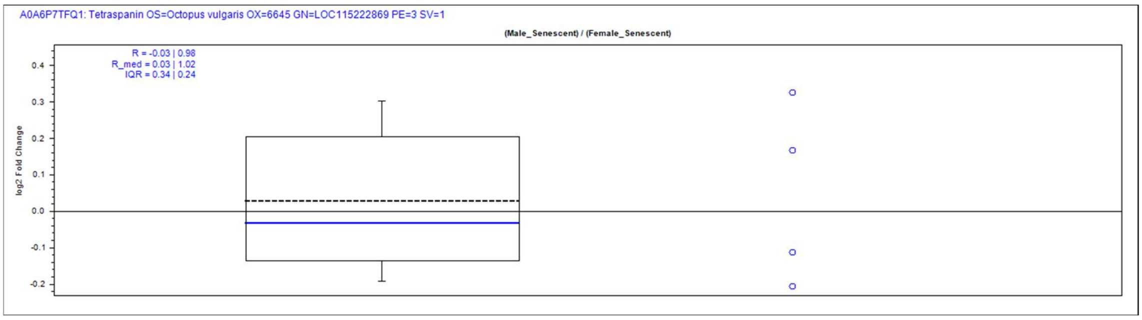

Annexin

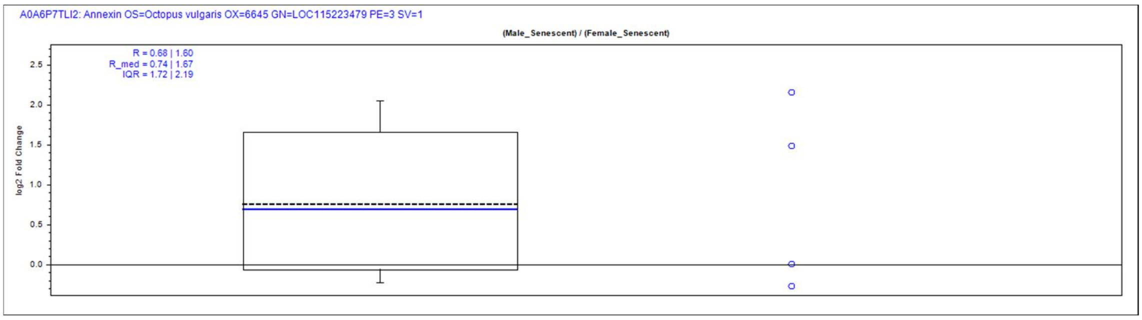

Histone H3

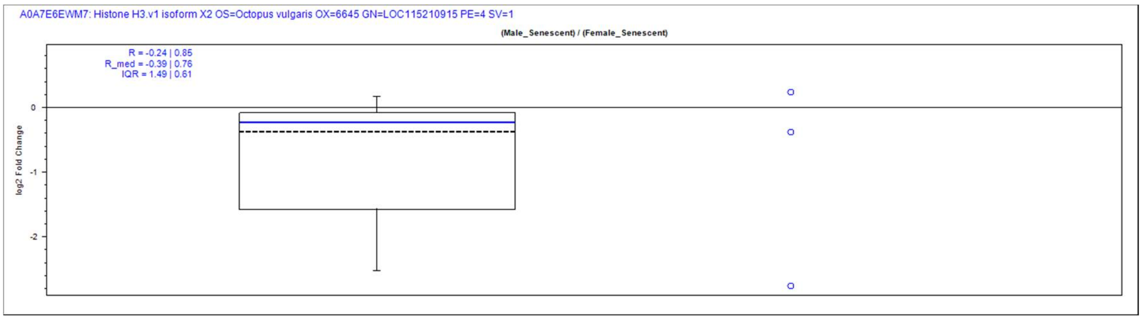

Lamin-B1 isoform

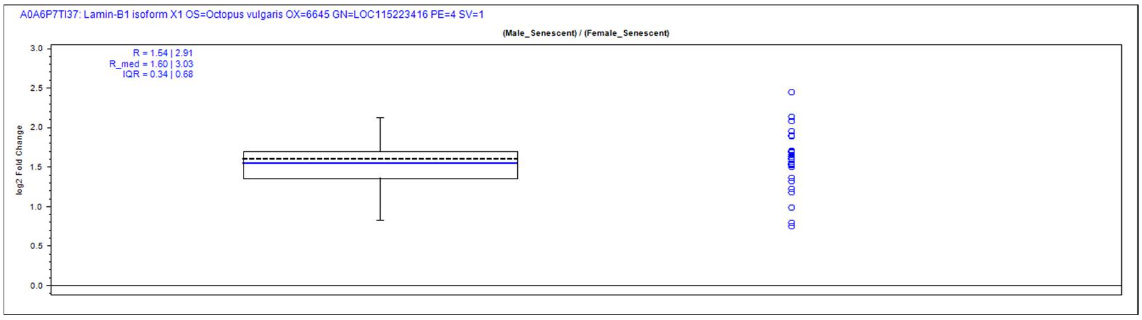

Papilin

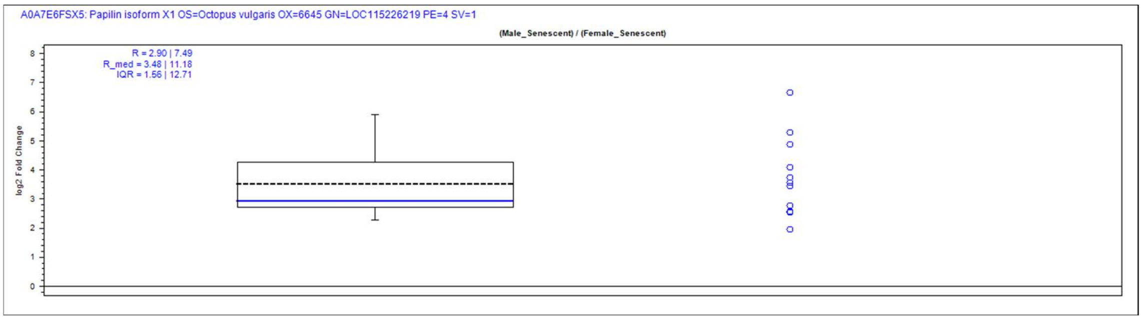

Kyphoscoliosis peptidase

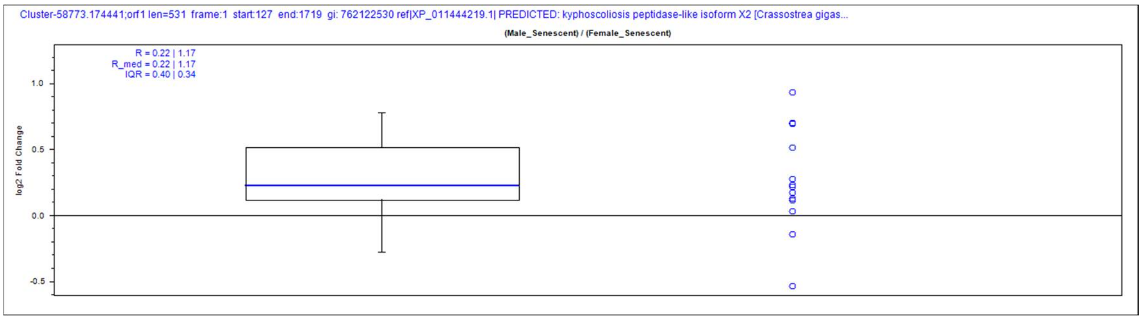

Alpha tubulin

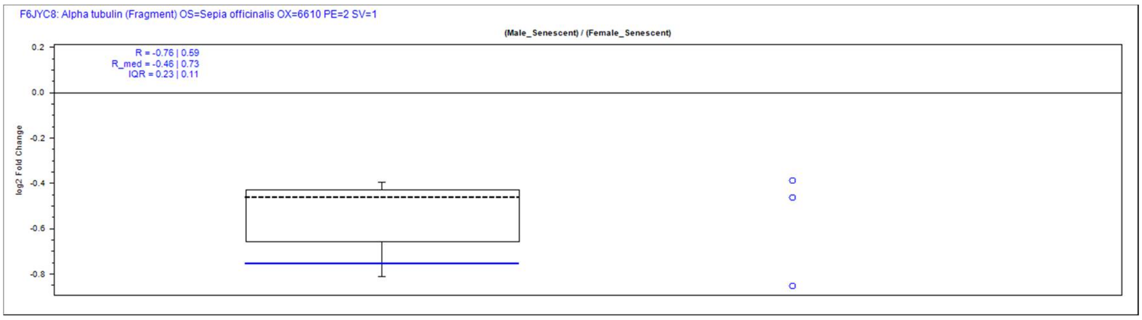

Deoxynucleoside triphosphate triphosphohydrolase SAMHD1

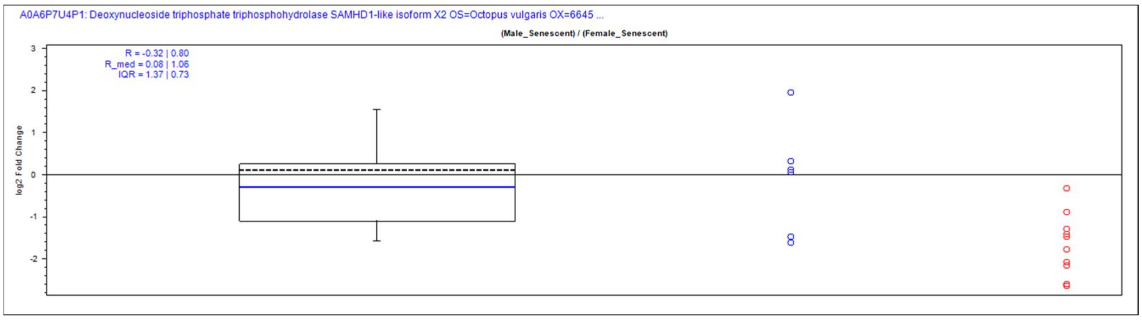

C1 family cathepsin L2

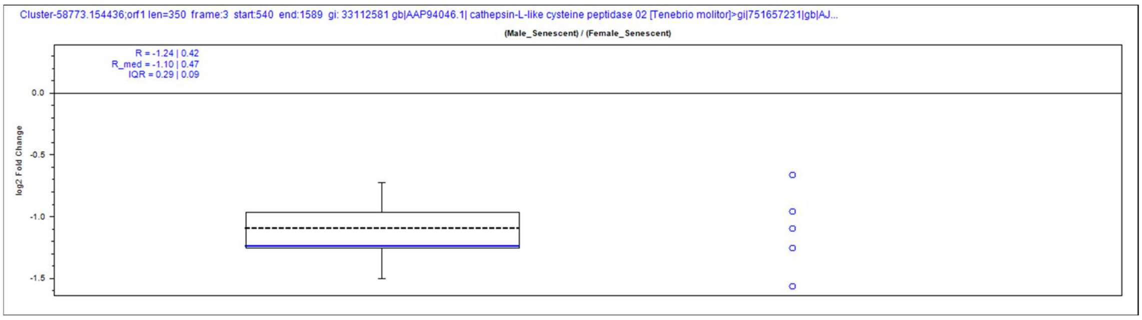

Calcium-dependent protein kinase 12 isoform X2

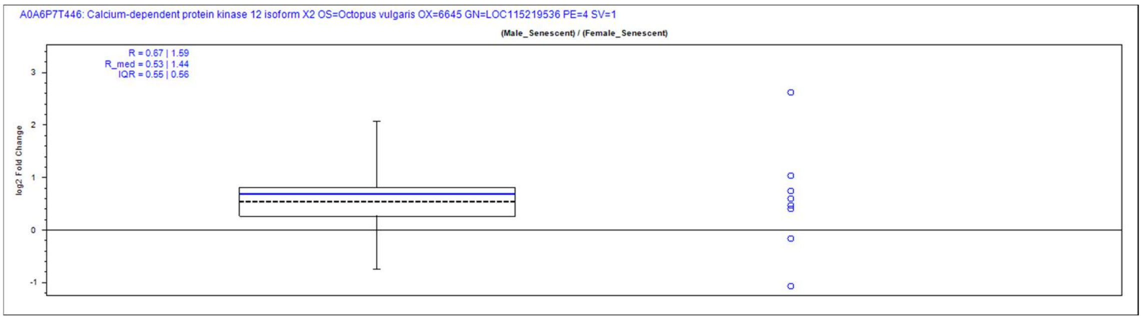

Beta-tubulin protein

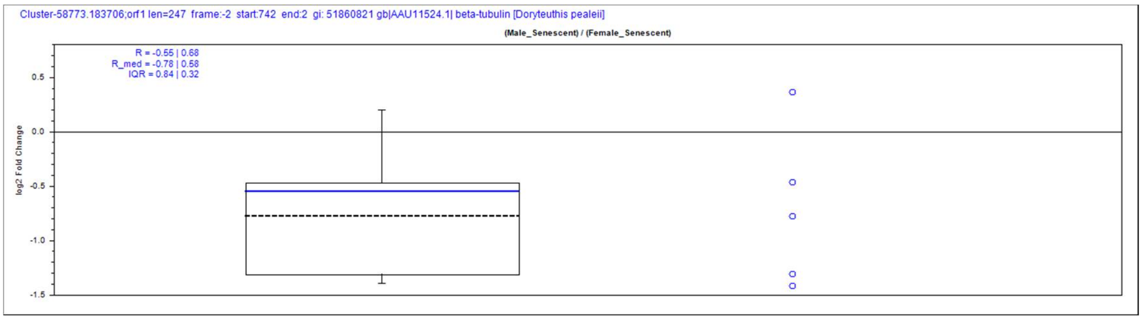

EF-hand domain-containing protein

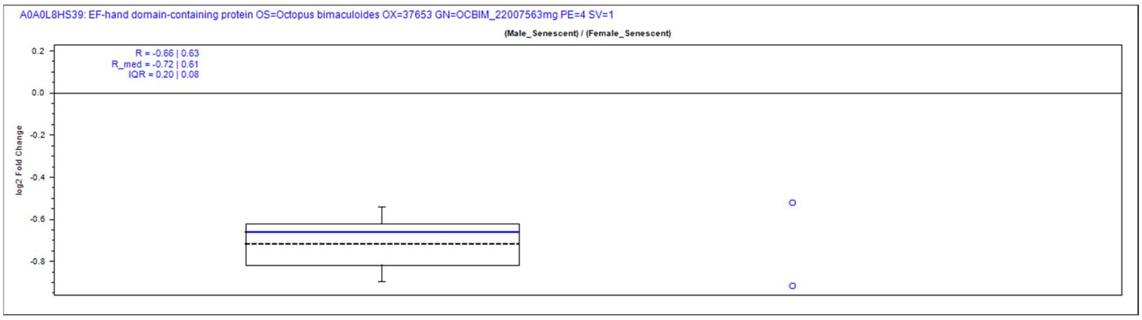

RING-type E3 ubiquitin transferase

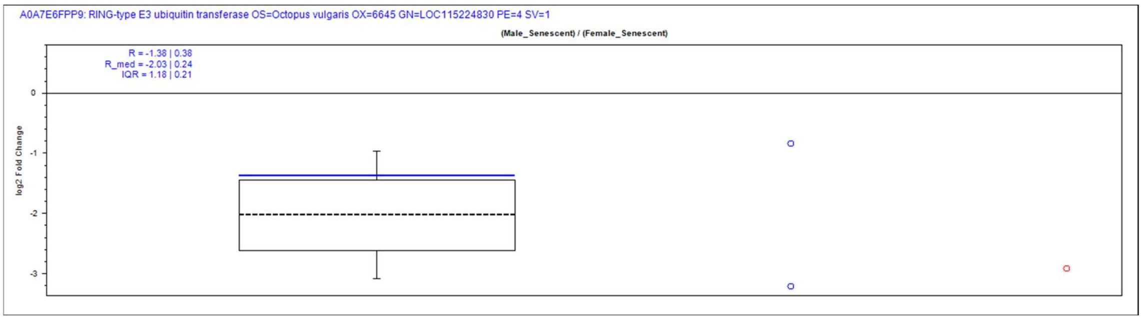

Mucin 4 protein

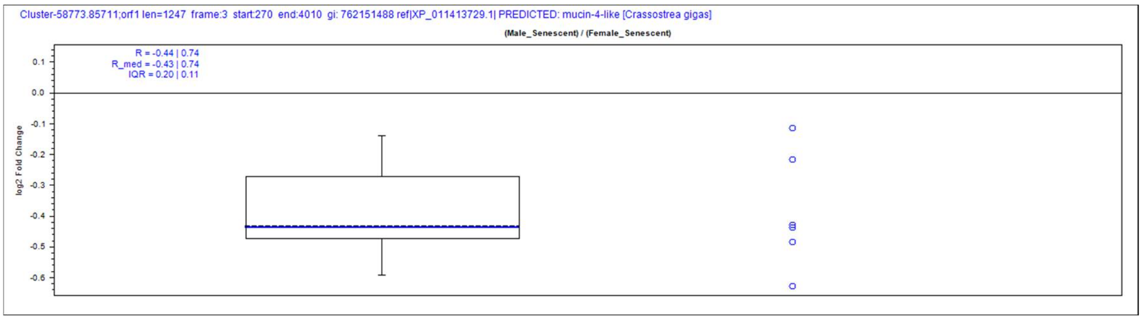

Neural cell adhesion molecule 2 isoform

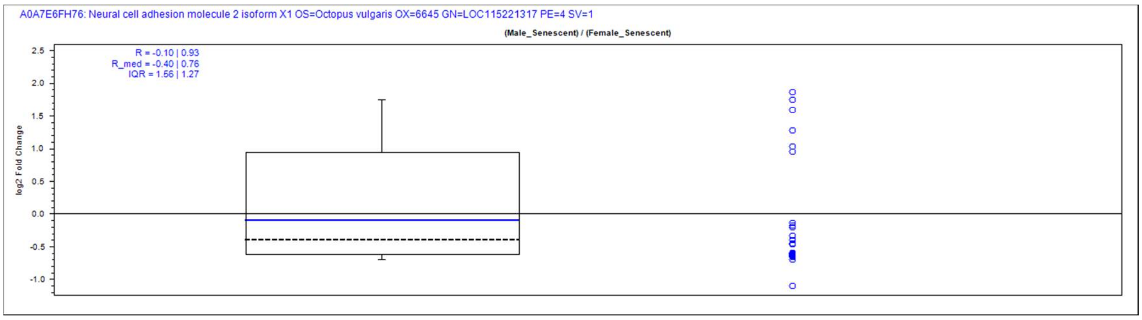

Titin

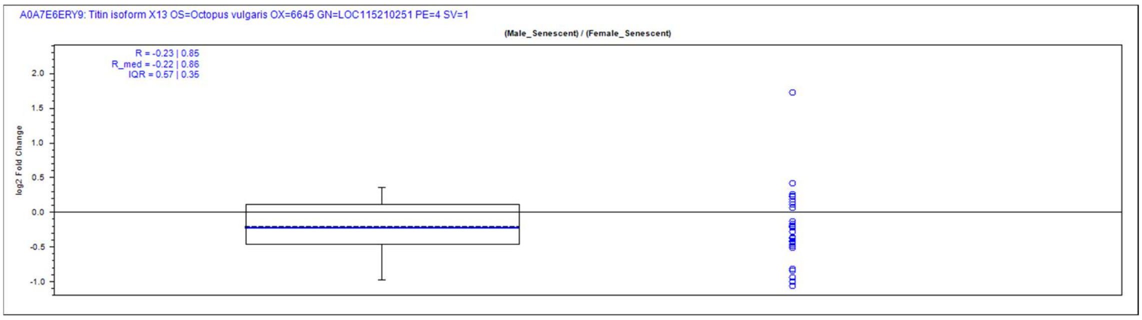

Golgi-associated plant pathogenesis-related protein 1-like

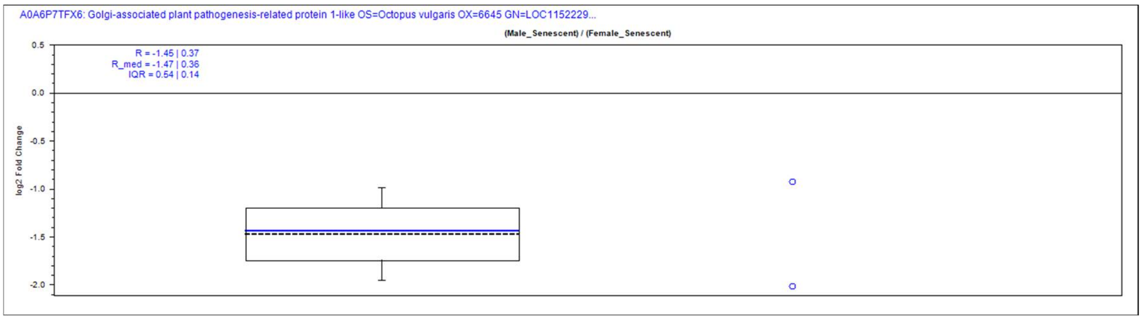

Supplement: Supplementary file 1 [file ijms-25-09953-s001.zip › Supplementary Data S5. Box plot analysis.pdf]
